# Supplementary material for: Neurological manifestations of coronavirus infections – a systematic review
Source: Ann Clin Transl Neurol. 2020 Aug 27;7(10):2057–71. doi: 10.1002/acn3.51166 (PMC7461163; doi:10.1002/acn3.51166)
Supplement: Supplementary file 1 — Table S1. Search string. Table S2. Pre‐defined criteria Newcastle‐Ottawa scale for risk of bias. Table S3. Risk of bias assessment. Table S4. Summary of studies assessing neurological manifestations and complication in the more clinically mild HCoVs: HCoV‐229E, HKU1, NL63 and/or OC43 infections. Table S5. Summary of studies assessing neurological manifestations and complication in MERS‐CoV infections. Table S6. Summary of studies assessing neurological manifestations and complication in SARS‐CoV‐1 infections. Table S7. Summary of studies assessing neurological manifestations and complication in SARS‐CoV‐2 infections. Table S8. Association of coronaviruses with multiple sclerosis. [file ACN3-7-2057-s001.docx]

**Table S1:** Predefined systematic search strings in PubMed Ovid EMBASE and Web of Science. Last search 26^th^ of July, 2020.

| **Databases** | **Studies produced** | **Search string** |
| --- | --- | --- |
| **Pubmed via Medline:** | 2966 | (coronavirus OR HCoV OR SARS-CoV-1 OR SARS-CoV-2 OR SARS-CoV1 OR SARS-CoV2 OR SARS) AND (neuroimaging OR magnetic resonance imaging OR MRI OR Neurology OR neurological complications OR neuroimmunology OR neurotropism OR neurologic OR central nervous system OR CNS OR head- CT OR brain-CT OR Positron emission tomography OR PET OR Nervous System Diseases [MeSH]) |
| **Embase:** | 5416 | (&quot;coronavirus&quot; OR &quot;HCoV&quot; OR &quot;SARS-CoV-1&quot; OR &quot;SARS-CoV-2&quot; OR &quot;SARS-CoV1&quot; OR &quot;SARS-CoV2&quot; OR &quot;SARS&quot;) AND (&quot;neuroimaging&quot; OR &quot;magnetic resonance imaging&quot; OR &quot;MRI&quot; OR &quot;Neurolog*&quot; OR &quot;neurological complications&quot; OR &quot;neuroimmunology&quot; OR &quot;neurotropism&quot; OR &quot;central nervous system&quot; OR &quot;CNS&quot; OR &quot;head-CT&quot; OR &quot;brain-CT&quot; OR &quot;Positron emission tomography&quot; OR &quot;PET&quot; OR &quot;neurologic disease&quot;) |
| **Web of Science:** | 3813 | (coronavirus OR HCoV OR SARS-CoV-1 OR SARS-CoV-2 OR SARS-CoV1 OR SARS-CoV2 OR SARS) AND TOPIC: (neuroimaging OR magnetic resonance imaging OR MRI OR Neurology OR neurological complications OR neuroimmunology OR neurotropism OR neurologic OR central nervous system OR CNS OR head-CT OR brain-CT OR Positron emission tomography OR PET OR Nervous System Diseases) |

**Table S2**: Newcastle-Ottawa scale to assess risk of bias for included studies on three domains: selection, comparability and exposure.

| **Cohort studies/Case series/Retrospective studies** |
| --- |
| **i. Selection** |
| **Ascertainment of exposure** |
| - Yes, i.e. authors checked coronavirus-status e.g. via RT-PCR in sputum* |
| - No description |
| **Representativeness of cohort** |
| - Truly representative of the average community, e.g. consecutive inclusions, all cases between date *x* and *y** |
| - Somewhat representative of the average community* |
| - Selected group of users, e.g. nurses, volunteers |
| - No description of derivation of cohort |
| **Demonstration that outcome of interest was not present at start of study** |
| - Yes* |
| - No |
| **ii. Comparability** |
| - Study controls for most important factor, e.g. age-and/or sex-matched controls* |
| - Study controls for additional factors, e.g. statistics adjusted for smoking* |
| - No statement |
| **iii. Exposure** |
| Assessment of outcome |
| - Independent blind assessment* |
| - No description |
| Was follow-up long enough for outcomes to occur |
| - Yes* |
| - No |
| Adequacy of follow-up of cohorts of patients |
| - Compete follow-up of all subjects* |
| - Subjects lost to follow-up unlikely to introduce bias (small number lost <= 10%)* |
| - Subjects lost to follow-up potentially to introduce bias (small number lost > 10%) |
| - No statement |
|  |
| **Case-control studies** |
| **i. Selection** |
| **Case definition adequate** |
| - Yes, i.e. authors checked coronavirus-status e.g. via RT-PCR in sputum* |
| - No description |
| **Representativeness of cases** |
| - Consecutive cases, i.e. mentioning of consecutive inclusion or obviously consecutive* |
| - Random inclusion of cases, i.e. usage of adequate randomization procedure* |
| - Potential for selection bias or not stated |
| **Selection of controls** |
| - Community controls/drawn from the same pool* |
| - Not drawn from the same pool |
| - No description |
| **Definition of controls** |
| - No history of disease (endpoint)* |
| - History of disease which does likely not introduce bias, e.g. prior appendectomy, prior influenza* |
| - No description |
| **ii. Comparability** |
| - Study controls for most important factor, e.g. age- and/or sex-matched controls* |
| - Study controls for additional factor, e.g. statistics adjusted for smoking* |
| No statement |
| **iii. Exposure** |
| **Assessment of outcome** |
| Independent blind assessment, i.e. mentioning blinding during any step of the experimental procedures* |
| No statement |
| **Cases and controls treated similarly throughout** |
| Yes, e.g. same MRI protocol, same treatment schedule* |
| Obviously differently treated, e.g. handed-out questionnaire vs telephone-questionnaire |
| **Adequacy of exclusion of patients** |
| No obvious exclusions* |
| Subjects excluded unlikely to introduce bias (small number excluded <= 10%)* |
| Subjects excluded potentially to introduce bias (small number excluded > 10%) |

Based on the Newcastle-Ottawa Scale (NOS) for assessing the quality of nonrandomized studies in meta-analyses.^1^ Two slightly different versions were used to evaluate risk of bias in cohort studies, case series and retrospective studies versus case-control studies, respectively. Each study was evaluated on the sub-item of each domain, e.g. whether the study confirmed coronavirus infection via reverse-transcriptase polymerase chain reaction (RT-PCR). Low risk of bias by each sub-item is rewarded with a star (*). There can be a maximum of one star per sub-item in the selection and exposure domain and two stars for the comparability dimension.

1. Wells GA, Tugwell P, O’Connell D, et al. The Newcastle-Ottawa Scale (NOS) for assessing the quality of nonrandomized studies in meta-analyses. 2015.

**Table S3**: The results of the risk of bias assessment of the included studies with 10 or more included subjects according to the Newcastle-Ottawa scale for nonrandomized studies^1^.

| **First author** | **Title** | **Year** | **Study type** | **S1** | **S2** | **S3** | **S4** | **C1** | **C2** | **E1** | **E2** | **E3** |
| --- | --- | --- | --- | --- | --- | --- | --- | --- | --- | --- | --- | --- |
| Alperovitch | Viral antibody titers, immunogenetic markers, and their interrelations in multiple sclerosis patients and controls | 1991 | Case-control | * | * | * | * | * | * | - | * | * |
| Ameres | Association of neuronal injury blood marker neurofilament light chain with mild-to-moderate COVID-19 | 2020 | Case series | * | - | * | N/A | * | * | - | * | * |
| Arbour | Neuroinvasion by human respiratory coronaviruses | 2000 | Case-control | * | - | - | * | - | - | * | - | * |
| Beltran-Corbellini | Acute-onset smell and taste disorders in the context of Covid-19: a pilot multicenter PCR-based case-control study | 2020 | Case-control | * | - | - | * | * | * | - | - | * |
| Benussi | Clinical characteristics and outcomes of inpatients with neurologic disease and COVID-19 in Brescia, Lombardy, Italy | 2020 | Retrospective cohort | * | * | - | N/A | * | * | - | * | * |
| Burks | 2 coronaviruses isolated from central nervous system tissue of 2 multiple sclerosis patients | 1980 | Case-control | * | - | - | * | - | - | - | * | * |
| Cantador | Incidence and consequences of systemic arterial thrombotic events in COVID-19 patients | 2020 | Retrospective cohort | - | * | - | N/A | - | - | - | * | * |
| Capelli | Anosmia and COVID-19 in south Lombardy: description of the first cases series in Europe | 2020 | Retrospective cohort | - | - | - | N/A | - | - | - | * | * |
| Carfi | Persistent Symptoms in Patients After Acute COVID-19 | 2020 | Cohort | * | * | * | N/A | - | - | - | * | * |
| Carignan | Anosmia and dysgeusia associated with SARS-CoV-2 infection: an age-matched case-control study | 2020 | Case-control | * | * | * | * | * | * | - | * | * |
| Carman | Viral etiological causes of febrile seizures for respiratory pathogens (EFES Study) | 2019 | Prospective cohort | * | * | * | N/A | * | - | - | * | * |
| Chen | Clinical characteristics of 113 deceased patients with coronavirus disease 2019: retrospective study | 2020 | Retrospective case series | * | * | - | N/A | - | - | - | * | * |
| Chen | Epidemiological and clinical characteristics of 99 cases of 2019 novel coronavirus pneumonia in Wuhan, China: a descriptive study | 2020 | Retrospective case series | * | * | - | N/A | - | - | - | * | * |
| Chen | Syncope, Near Syncope, or Nonmechanical Falls as a Presenting Feature of COVID-19 | 2020 | Retrospective case series | - | * | * | N/A | - | - | - | * | * |
| Chiesa | Patterns of smell recovery in 751 patients affected by the COVID-19 outbreak | 2020 | Prospective survey | * | * | * | N/A | * | - | - | * | * |
| Chougar | Retrospective Observational Study of Brain Magnetic Resonance Imaging Findings in Patients with Acute SARS-CoV-2 Infection and Neurological Manifestations | 2020 | Retrospective case series | * | * | * | N/A | * | - | - | * | * |
| Coolen | Early postmortem brain MRI findings in COVID-19 non-survivors | 2020 | Case series | * | * | * | N/A | - | - | - | * | * |
| Dessau | Coronaviruses in spinal fluid of patients with acute monosymptomatic optic neuritis | 1999 | Case-control | * | - | - | * | - | - | - | * | * |
| Dessau | Coronaviruses in brain tissue from patients with multiple sclerosis | 2001 | Case-control | * | * | - | * | - | - | - | - | * |
| Dominguez | Detection of four human coronaviruses in respiratory infections in children: a one-year study in Colorado | 2009 | Retrospective cohort | * | * | - | N/A | * | - | * | * | * |
| Dogra | Hemorrhagic stroke and anticoagulation in COVID-19 | 2020 | Retrospective case series | * | - | * | N/A | - | - | * | * | * |
| Edler | Dying with SARS-CoV-2 infection-an autopsy study of the first consecutive 80 cases in Hamburg, Germany | 2020 | Case series | * | * | * | N/A | * | - | - | * | * |
| Escalard | Treatment of Acute Ischemic Stroke due to Large Vessel Occlusion With COVID-19: Experience From Paris | 2020 | Case series | * | * | * | N/A | * | - | - | * | * |
| Fazzini | Cerebrospinal fluid antibodies to coronavirus in patients with Parkinson's disease | 1992 | Case-control | * | - | * | - | * | - | * | * | - |
| Fleming | Antigenic assessment of coronaviruses isolated from patients with multiple sclerosis | 1987 | Case-control | * | - | * | - | - | - | - | * | * |
| Franceschi | Neurovascular Complications in COVID-19 Infection: Case Series | 2020 | Case series | * | - | * | N/A | - | - | - | * | * |
| Freni | Symptomatology in head and neck district in coronavirus disease (COVID-19): A possible neuroinvasive action of SARS-CoV-2 | 2020 | Prospective case series | * | - | * | N/A | * | - | - | * | * |
| Galanopo | EEG findings in acutely ill patients investigated for SARS-CoV-2/COVID-19: A small case series preliminary report | 2020 | Retrospective case series | * | - | * | N/A | * | - | - | * | * |
| Gomez-Iglesias | An Online Observational Study of Patients With Olfactory and Gustory Alterations Secondary to SARS-CoV-2 Infection | 2020 | Observational | - | - | * | N/A | * | - | - | * | * |
| Gu | Multiple organ infection and the pathogenesis of SARS | 2005 | Retrospective case series | * | * | - | N/A | - | - | - | * | * |
| Helms | Neurologic Features in Severe SARS-CoV-2 Infection | 2020 | Observational case series | * | * | - | N/A | - | - | - | * | * |
| Hernandez-Fernandez | Cerebrovascular disease in patients with COVID-19: neuroimaging, histological and clinical description | 2020 | Retrospective case series | * | * | * | N/A | * | * | - | * | * |
| Hopkins | Presentation of new onset anosmia during the COVID-19 pandemic | 2020 | Survey based cohort | * | - | * | N/A | * | - | - | * | * |
| Hornuss | Anosmia in COVID-19 patients | 2020 | Case-control | * | * | * | - | * | - | - | * | * |
| Hovanec | Detection of antibodies to human coronaviruses 229E and OC43 in the sera of multiple sclerosis patients and normal subjects | 1983 | Case-control | * | - | - | * | * | - | - | * | * |
| Huang | Clinical features of patients infected with 2019 novel coronavirus in Wuhan, China | 2020 | Prospective | * | * | - | N/A | * | - | - | * | * |
| Jain | COVID-19 related neuroimaging findings: A signal of thromboembolic complications and a strong prognostic marker of poor patient outcome | 2020 | Retrospective cohort | * | - | * | N/A | * | * | - | * | * |
| Joob | Alteration of consciousness as initial presentation in COVID-19: Observation | 2020 | Retrospective cohort | - | - | - | N/A | - | - | - | * | * |
| Jevsnik | The Role of Human Coronaviruses in Children Hospitalized for Acute Bronchiolitis, Acute Gastroenteritis, and Febrile Seizures: A 2-Year Prospective Study | 2016 | Prospective cohort | * | * | - | N/A | * | * | - | * | * |
| Kanberg | Neurochemical evidence of astrocytic and neuronal injury commonly found in COVID-19 | 2020 | Cohort | * | - | * | N/A | * | * | * | * | * |
| Karadas | A prospective clinical study of detailed neurological manifestations in patients with COVID-19 | 2020 | Retrospective cohort | * | * | * | N/A | * | - | - | * | * |
| Kremer | Brain MRI Findings in Severe COVID-19: A Retrospective Observational Study | 2020 | Retrospective observational | * | * | * | N/A | * | - | - | * | * |
| Kriesel | Multiple sclerosis attacks are associated with picornavirus infections | 2004 | Retrospective case series | * | * | - | N/A | - | - | * | * | * |
| Lau | Coronavirus HKU1 and other coronavirus infections in Hong Kong | 2006 | Prospective and retrospective | * | * | - | N/A | * | - | - | * | * |
| Lechien | Olfactory and gustatory dysfunctions as a clinical presentation of mild-to-moderate forms of the coronavirus disease (COVID-19): a multicenter European study | 2020 | Cohort | * | * | * | N/A | - | - | - | * | * |
| Levinson | Time course of anosmia and dysgeusia in patients with mild SARS-CoV-2 infection | 2020 | Case series | * | - | * | N/A | * | - | - | * | * |
| Li | Coronavirus Infections in the Central Nervous System and Respiratory Tract Show Distinct Features in Hospitalized Children | 2016 | Cohort | * | - | - | N/A | * | - | - | * | * |
| Liguori | Subjective neurological symptoms frequently occur in patients with SARS-CoV2 infection | 2020 | Prospective observational | - | - | * | N/A | * | - | - | * | * |
| Lodigiani | Venous and arterial thromboembolic complications in COVID-19 patients admitted to an academic hospital in Milan, Italy | 2020 | Retrospective cohort | - | * | * | N/A | * | * | - | * | * |
| Lu | New-onset acute symptomatic seizure and risk factors in Corona Virus Disease 2019: A Retrospective Multicenter Study | 2020 | Retrospective | * | * | - | N/A | * | - | - | * | * |
| Madden | Coronavirus antibodies in sera from patients with multiple sclerosis and matched controls | 1981 | Case-control | * | - | * | * | * | * | - | * | * |
| Mahamme | Imaging in Neurological Disease of Hospitalized COVID-19 Patients: An Italian Multicenter Retrospective Observational Study | 2020 | Retrospective observational | * | * | * | N/A | * | - | - | * | * |
| Mao | Neurologic Manifestations of Hospitalized Patients With Coronavirus Disease 2019 in Wuhan, China | 2020 | Retrospective, observational case series | * | * | - | N/A | * | - | - | * | * |
| Merkler | Risk of Ischemic Stroke in Patients With Coronavirus Disease 2019 (COVID-19) vs Patients With Influenza | 2020 | Retrospective cohort | * | - | * | N/A | * | * | - | * | * |
| Murray | Detection of coronavirus RNA and antigen in multiple sclerosis brain | 1992 | Case-control | * | * | * | * | - | - | - | * | * |
| Noorwali | Descriptive epidemiology and characteristics of confirmed cases of Middle East respiratory syndrome coronavirus infection in the Makkah Region of Saudi Arabia, March to June 2014 | 2015 | Retrospective case series | * | * | - | N/A | - | - | - | * | * |
| Okusaga | Association of seropositivity for influenza and coronaviruses with history of mood disorders and suicide attempts | 2011 | Case-control | * | - | - | * | * | * | - | * | * |
| Paterson | The emerging spectrum of COVID-19 neurology: clinical, radiological and laboratory findings | 2020 | Case series | * | * | * | N/A | * | - | - | * | * |
| Peng | Improved Early Recognition of Coronavirus Disease-2019 (COVID-19): Single-Center Data from a Shanghai Screening Hospital | 2020 | Cross-sectional | * | * | - | N/A | - | - | - | * | * |
| Pinna | Neurological manifestations and COVID-19: Experiences from a tertiary care center at the Frontline | 2020 | Retrospective case series | * | - | * | N/A | * | . | . | * | * |
| Pokorn | Respiratory and Enteric Virus Detection in Children | 2017 | Prospective | * | * | - | N/A | * | - | - | * | * |
| Pons-Escoda | Neurologic Involvement in COVID-19: Cause or Coincidence? A Neuroimaging Perspective | 2020 | Cross-sectional study | * | - | * | N/A | * | - | - | * | * |
| Qin | Dysregulation of immune response in patients with COVID-19 in Wuhan, China | 2020 | Retrospective cohort | * | * | - | N/A | * | - | - | * | * |
| Radmanesh | COVID-19 -associated Diffuse Leukoencephalopathy and Microhemorrhages | 2020 | Case series | * | - | * | N/A | * | - | - | * | * |
| Radmanesh | Brain Imaging Use and Findings in COVID-19: A Single Academic Center Experience in the Epicenter of Disease in the United States | 2020 | Retrospective cohort | * | - | * | N/A | * | - | - | * | * |
| Reddy | Cerebrovascular Disease in Patients with COVID-19: A Review of the Literature and Case Series | 2020 | Case series | - | - | * | N/A | * | - | - | * | * |
| Riski | Coronavirus infections of man associated with diseases other than the common cold | 1980 | Retrospective cohort | * | * | - | N/A | * | * | - | * | * |
| Romero-Sanchez | Neurologic manifestations in hospitalized patients with COVID-19: The ALBACOVID registry | 2020 | Observational retrospective cohort | * | * | * | N/A | * | - | - | * | * |
| Saad | Clinical aspects and outcomes of 70 patients with Middle East respiratory syndrome coronavirus infection: a single-center experience in Saudi Arabia | 2014 | Retrospective case series | * | * | - | N/A | * | * | - | * | * |
| Scullen | Coronavirus 2019 (COVID-19)-Associated Encephalopathies and Cerebrovascular Disease: The New Orleans Experience | 2020 | Retrospective cross-sectional | * | * | * | N/A | * | - | - | * | * |
| Severance | Coronavirus immunoreactivity in individuals with a recent onset of psychotic symptoms | 2009 | Retrospective | * | * | - | N/A | * | * | - | * | * |
| Sheng | The effects of disease severity, use of corticosteroids and social factors on neuropsychiatric complaints in severe acute respiratory syndrome (SARS) patients at acute and convalescent phases | 2005 | Cross-sectional | - | * | - | N/A | * | - | - | * | * |
| Sierpinski | Sex differences in the frequency of gastrointestinal symptoms and olfactory or taste disorders in 1942 nonhospitalized patients with coronavirus disease 2019 (COVID-19) | 2020 | Cross-sectional survey-based | * | - | * | N/A | * | - | - | * | * |
| Solomon | Neuropathological Features of Covid-19 | 2020 | Case series | * | * | - | N/A | * | - | - | * | * |
| Speth | Mood, anxiety and olfactory dysfunction in COVID-19: evidence of central nervous system involvement? | 2020 | Prospective cross-sectional | * | - | * | N/A | * | - | - | * | * |
| Speth | Olfactory Dysfunction and Sinonasal Symptomatology in COVID-19: Prevalence, Severity, Timing, and Associated Characteristics | 2020 | Prospective cross-sectional | * | - | * | N/A | * | - | - | * | * |
| Spoldi | Isolated olfactory cleft involvement in SARS-CoV-2 infection: prevalence and clinical correlates | 2020 | Retrospective cross-sectional | - | - | * | N/A | * | - | * | * | * |
| Stewart | Human coronavirus gene expression in the brains of multiple sclerosis patients | 1992 | Case-control | * | - | * | * | - | - | - | * | * |
| Sweid | Cerebral ischemic and hemorrhagic complications of coronavirus disease 2019 | 2020 | Retrospective case series | - | - | * | N/A | * | - | - | * | * |
| Tsivgoulis | Quantitative evaluation of olfactory dysfunction in hospitalized patients with Coronavirus [2] (COVID-19) | 2020 | Prospective cohort | * | - | * | N/A | * | - | - | * | * |
| Vacchiano | Early neurological manifestations of hospitalized COVID-19 patients | 2020 | Prospective survey-based | * | * | * | N/A | * | - | - | * | * |
| Varatharaj | Neurological and neuropsychiatric complications of COVID-19 in 153 patients: a UK-wide surveillance study | 2020 | Surveillance study | * | - | * | N/A | * | - | - | * | * |
| Vespignani | Report on Electroencephalographic Findings in Critically Ill Patients with COVID-19 | 2020 | Case series | * | - | * | N/A | * | - | - | * | * |
| Wei | Endocrine cells of the adenohypophysis in severe acute respiratory syndrome (SARS) | 2009 | Case-control | - | - | - | * | * | - | - | * | * |
| Xiong | New onset neurologic events in people with COVID-19 infection in three regions in China | 2020 | Retrospective cohort | * | * | * | N/A | * | * | - | * | * |
| Yaghi | SARS-CoV-2 and Stroke in a New York Healthcare System | 2020 | Retrospective cohort | * | * | * | N/A | * | * | - | * | * |

S1-S4: Selection domain (only S1-S3 for cohort studies, case series and retrospective studies), C1-C2: comparability domain, E1-E3: exposure domain.

1. Wells GA, Tugwell P, O’Connell D, et al. The Newcastle-Ottawa Scale (NOS) for assessing the quality of nonrandomized studies in meta-analyses. 2015.

**Table S4**: Summary of studies assessing neurological manifestations and complication in the more clinically mild HCoVs: HCoV-229E, HKU1, NL63 and/or OC43 infections.

| **First author** | **Year** | **Virus** | **Study type** | **Multi-center** | **Country** | **Number of patients** | **Controls** | **Corona-proof** | **Summary findings** |
| --- | --- | --- | --- | --- | --- | --- | --- | --- | --- |
| Cabeca | 2011 | HCoV NL63 | Case report | No | Brazil | 1 | N/A | RT-PCR NPH swab | A 46-year-old female with diabetes mellitus type 2. Developed hemorrhagic pneumonia. At day 9 of admission, brain CT showed brain edema. |
| Carman | 2019 | HCoV 229E, OC43 | Prospective cohort study | Yes | Turkey | 174 children | N/A | Multiplex array for different viruses, NPH swab | During 13-month follow-up, 192 children with febrile seizures have been admitted. Overall, the most common virus associated with febrile seizure was adenovirus. OC43 was most commonly associated with febrile seizures in children younger than 12 months (approximately 6% of children with upper respiratory infections). OC43 can cause febrile seizures, but remains a rare cause. |
| Dominguez | 2009 | HCoV 229E, HKU1, NL63, OC43 | Retrospective cohort study | No | USA | 84 children | N/A | RT-PCR NPH swab | 1683 respiratory specimens from children were assessed for respiratory viruses over a 1-year period. Coronavirus RNAs were detected in 84 of the specimens (5%). 5 patients (8%) presented with seizures or meningoencephalitis. |
| Fazzini | 1992 | HCoV 229E, OC43 | Case-control | No | USA | 20 patients with PD | 18 controls, 20 other neurological diseases | ELISA in CSF | PD patients had an elevated (p < 0.05) mean OD response to mouse hepatitis virus JHM (0.0856 VS. 0.0207) and mouse hepatitis virus A59 (0.1722 vs. 0.0636). Response (p > 0.05) to OC43 (0.0839 vs. 0.0071) was greater than that to 229E (0.1261 vs. 0.0743). When compared to other neurological diseases, PD patients had an elevated mean OD response to mouse hepatitis virus JHM (0.0856 vs. 0.0267, p < 0.05). Responses (p > 0.05) to mouse hepatitis virus A59 (0.1722 vs. 0.0929) and OC43 (0.0839 vs. 0.0446) were greater than that to 229E (0.1261 vs. 0.0946). These results suggest that there may be an association between coronavirus and PD. |
| Jevsnik | 2016 | HCoV 229E, HKU1, NL63, OC43 | Prospective study | No | Slovenia | 278 children under 6 years with febrile seizures | N/A | RT-PCR NPH swab | 6164 children were admitted during a 2 year observational period out of which 278 had febrile seizures. HCoVs were most often detected in children with febrile seizures (19/192, 9.9%). Among them, OC43 was detected most frequently (12/19, 63%), followed by HKU1 3/19 (16%), 229E 1/19 (5%). NL63 was not detected in patients with febrile seizures. 9/19 children (48%) were positive for HCoV only. |
| Kriesel | 2004 | HCoV 229E, OC43 | Retrospective case series | No | USA | 16 MS patients | N/A | RT-PCR NPH swab | Coronaviruses (among other viruses) were not detected in NPH swabs of MS patients |
| Lau | 2006 | HCoV 229E, HKU1, NL63, OC43 | Prospective and retrospective study | Yes | China | 87 | N/A | RT-PCR NPH swab | Coronavirus was detected in 87 out of 4181 patients with acute respiratory tract infections (2.1%: 1.3% OC43, 0.4% NL63, 0.3% HKU1, 0.1% 229E). 11/13 patients with HKU1 were children. Relatively high incidence of febrile seizures in HKU1 (6/11 children), significantly higher compared to OC43. 1 case with aseptic meningitis (OC43), 3 cases with breakthrough seizure (2 with HKU1, 1 with NL63). |
| Morfopoulou | 2016 | HCoV OC43 | Case report | No | UK | 1 child | N/A | RNA sequencing, RT-PCR brain | A 11-month-old boy with severe combined immunodeficiency with symptoms of viral encephalitis. Conventional diagnostic PCR was negative. RNA sequencing of a brain biopsy sample obtained 2 months after the onset of symptoms showed the presence of HCoV OC43. |
| Nilsson | 2020 | HCoV OC43 | Case report | No | Sweden | 1 child | N/A | Metagenomic next-generation sequencing, RT-PCR CSF, brain | A 9-month old child with pre-B acute lymphoblastic leukaemia. During chemotherapy the child had a persistent OC43 respiratory infection and later developed progressive encephalitis. CSF was negative for pathogens including OC43, but a brain biopsy was OC43-positive by metagenomic next-generation sequencing. |
| Okusaga | 2011 | HCoV NL63 | Case-control | No | USA | 117 recurrent unipolar and bipolar disorder, 99 suicide attempted | 39 controls | ELISA blood | Seropositivity for coronaviruses were associated with a history of mood disorders but not with the specific diagnosis of unipolar or bipolar depression. 86.7% of major depression patients, 79.8% of suicide attempted patients and 16% of controls were seropositive for NL63. |
| Pokorn | 2017 | HCoV 229E, HKU1, NL63, OC43 | Prospective study | No | Slovenia | 192 children < 6 years with febrile seizures | 156 | RT-PCR NPH swab | The viruses most strongly associated with febrile seizures were influenza, respiratory syncytial virus, parainfluenza, human coronavirus, and rotavirus. HCoVs were significantly more frequent in children with febrile seizures compared to healthy controls. The detected viruses did not influence clinical features of febrile seizures. 19 out of 192 patients (9.9%) with febrile seizures were HCoV positive (compared to 1.3% of healthy controls). |
| Riski | 1980 | HCoV OC43 | Retrospective cohort study | No | Finland | 28 | N/A | Complement fixation in serum (antibodies) | Out of 14000 screened sera from a patient with acute infectious diseases, 28 were positive for OC43 (0.2%). Four of these patients (14%) had neurological symptoms and/or complications: 1 meningitis, 1 seizures, 1 headache, 1 vertigo |
| Severance | 2009 | HCoV 229E, HKU1, NL63, OC43 | Retrospective study | No | USA | 106 patients with recent onset schizophrenia | 196 non-psychotic controls | ELISA blood | Higher antibody titers for HKU1, NL63, and OC43 in patients versus controls. All 4 HCoV were more seroprevalent in patients versus controls. Among patients, NL63 was associated with schizophrenia but not mood disorders. |
| Sharma | 2019 | HCoV OC43 | Case report | No | USA | 1 | N/A | RT-PCR blood | A 5-year-old boy with progressive lower extremity weakness and pain 3 days prior to onset of unilateral peripheral facial palsy and dysphagia. Diagnosis of Guillain–Barré syndrome variant was established. RT-PCR from blood was positive for OC43. Brain/spinal MRI with enhancement of left cranial nerves X and XI as well as anterior/posterior cervical nerve roots. |
| Turgay | 2015 | HCoV 229E, OC43 | Case report | No | Turkey | 1 child | N/A | RT-PCR NPH swab | A 3-year-old girl with reduced swallowing, chewing and speech functions and inability to walk 1 day after beginning of fever and cough. Deep tendon reflexes absent. CSF normal. Brain/spinal MRI normal. Intravenous immunoglobulins with therapeutic efficacy. |
| Yeh | 2004 | HCoV 229E, OC43 | Case report | No | USA | 1 child | N/A | RT-PCR NPH swab and CSF | A 15-year-old boy with numbness in lower extremities and difficulties to walk for several days. Brain MRI with patchy areas of hyperintensity in the white matter tracts, particularly in the centrum semiovale as well as an area of hyperintensity in the left cerebellum adjacent to the superior aspect of the left middle cerebellar peduncle. Some lesions were contrast-enhancing, including that in the left centrum semiovale. OC43 was detected in NPH swab and CSF. Patient presumed to have acute disseminated encephalomyelitis. |

*Abbreviations: HCoV = human coronavirus; ISH = in situ hybridization; NPH = nasopharyngeal; OR = odds ratio; PD = parkinson’s disease; OD = optical density; RT-PCR = reverse transcriptase polymerase chain reaction.*

**Table S5**: Summary of studies assessing neurological manifestations and complication in MERS-CoV infections.

| **First author** | **Year** | **Study type** | **Multi-center** | **Country** | **Number of patients** | **Controls** | **Corona-proof** | **Summary findings** |
| --- | --- | --- | --- | --- | --- | --- | --- | --- |
| Algahtani | 2016 | Case series | No | Saudi Arabia | 2 | N/A | RT-PCR sputum | Case 1: 34-year-old female with diabetes mellitus type 2, two weeks after admission reduced consciousness (GCS 3/15), disseminated intravascular coagulation and subsequent multiorgan failure with brain stem dysfunction and death after 2 months. Case 2: 28-year-old male, after a couple of days upon admission, numbness in both legs, reduced nerve conduction velocity in lower extremity, axonal polyneuropathy (critical illness polyneuropathy), six months afterwards slow improvement. |
| Al-Hameed | 2017 | Case report | No | Saudi Arabia | 1 | N/A | RT-PCR tracheal aspirate | A 42-year-old female with type 2 diabetes mellitus and sudden onset diabetes insipidus and intracranial hemorrhage with intraventricular extension and tonsillar herniation on brain CT. |
| Arabi | 2015 | Case series | No | Saudi Arabia | 3 | N/A | RT-PCR NPH swab | Case 1: 74-year-old male with diabetes type 2, decreased motor strength left side, MRI with small vessel disease, day 24 reduced consciousness (GCS 3), MRI with multiple non-contrast-enhancing patchy hypointensities on T_1_-weighted image, hyperintensities on T_2_-weighted image and diffusion restriction, suggestive for acute disseminated encephalomyelitis (ADEM), CSF RT-PCR for MERS-HCoV negative, Case 2: 57-year-old with diabetes mellitus type 2 and vascular disease, day 8 of admission with MRI suggestive of stroke, Case 3: 45-year-old with diabetes type 2, low GCS on day 9. MRI with confluent non-contrast enhancing T_2_ hyperintensities suggestive for encephalitis. CSF RT-PCR for MERS-HCoV negative. |
| Kim | 2017 | Case series | No | South Korea | 4 | N/A | RT-PCR sputum | 2 Males/2 females (out of 23 MERS patients, 17%), 38 - 55-years-old, 3 of them with diabetes mellitus. Neurological complications delayed by 2–3 weeks after respiratory symptoms. Symptoms: hyporeflexia in both legs (3 patients) and leg weakness (2 patients). 1 patient with normal CSF, 3 patients without CSF. 1 patient with normal brain MRI. Clinical presentation suggestive of Bickerstaff’s encephalitis overlapping with Guillain-Barré syndrome, ICU-acquired weakness, infectious/toxic neuropathy. |
| Noorwali | 2015 | Retrospective case series | Yes | Saudi arabia | 261 | N/A | RT-PCR respiratory tract | Out of 227 MERS patients, 185 showed symptoms (71%), neurological symptoms were: fatigue (108 cases, 58%), headache (59 cases, 31%), altered consciousness (53 cases, 29%) and/or local neurological deficit (10 cases, 5%). Altered level of consciousness at time of diagnosis was a significant risk factor for increased mortality. |
| Saad | 2014 | Retrospective case series | No | Saudi Arabia | 70 | N/A | RT-PCR respiratory tract | Majority of patients were symptomatic (67/70, 96%), common neurological symptoms were: generalized fatigue (29, 43%), confusion (18, 27%), headache (9, 13%) and/or seizures (6, 9%). |

*Abbreviations: CoV = coronavirus; GCS = Glasgow Coma Scale; ISH = in situ hybridization; MERS = Middle Eastern respiratory syndrome; NPH = nasopharyngeal; OR = odds ratio; RT-PCR = reverse transcriptase polymerase chain reaction;*

**Table S6**: Summary of studies assessing neurological manifestations and complication in SARS-CoV-1 infections

| **First author** | **Year** | **Study type** | **Multi-center** | **Country** | **Number of patients** | **Controls** | **Corona-proof** | **Summary findings** |
| --- | --- | --- | --- | --- | --- | --- | --- | --- |
| Ding | 2003 | Case series | No | China | 3 | N/A | Unclear | Autopsy of three patients who died from SARS-CoV-1 (one of them with headache). All three patients had systemic vasculitis, localized fibrinoid necrosis, and infiltration of immune cells. In two patients, there was edema surrounding the small veins in the brain, with infiltration of the vascular walls by monocytes and lymphocytes. Brain tissue was slightly edematous, with patchy demyelination and focal neuronal degeneration. |
| Gu | 2005 | Retrospective study | No | China | 8 | 10 | RT-PCR and ISH blood and brain, ELISA blood | 18 autopsies of patients with suspected SARS-CoV-1, 8 cases (44%) were confirmed having SARS-CoV-1 (no description of neurological symptoms). ISH signals were confined to neurons in the hypothalamus and cortex. Edema and diffuse neurodegeneration was present in the brains of 6/8 (75%) confirmed cases of SARS-CoV-1, suggestively a sign of hypoxia/ischemia. Infection of neurons seemed to occur in only selected areas of the brain in all eight cases. SARS-CoV-1 viral sequences and pathologic changes were unique to brains of SARS-CoV-1-confirmed cases. |
| Hu | 2004 | Cohort study | Yes | China | 1291 | N/A | Unclear | Cerebrocardiovascular diseases were an independent risk factor in SARS-CoV-1 patients for critical conditions and multi-organ dysfunction in comparison with those without underlying diseases. The incidence rates of critical conditions and multi-organ dysfunction among the SARS-CoV-1 cases without underlying diseases, with other comorbid diseases and with cerebrocardiovascular diseases were 28%, 44%, and 58%, and 10%, 19%, and 27% respectively. |
| Hung | 2003 | Case report | No | China | 1 | N/A | RT-PCR swab and CSF | A 59-year-old woman with IgA nephropathy. Day 5 of admission, confusion and disorientation. Normal brain CT. Status epilepticus. RT-PCR in CSF positive for SARS-CoV-1. |
| Hwang | 2006 | Case report | No | China | 1 | N/A | Unclear | A 27-year-old female. 3 weeks after onset of first symptoms, she noted anosmia. Brain MRI normal. Anosmia persisted for 2 years. |
| Lau | 2004 | Case report | No | China | 1 | N/A | RT-PCR swab and CSF | A 32-year-old pregnant women with severe SARS, developed generalized tonic-clonic convulsion on day 22 of illness. RT-PCR CSF positive for SARS-CoV-1. |
| Leung | 2005 | Case series | No | China | 8 | N/A | In situ hybridization muscle | Skeletal muscle autopsy findings from 8 consecutive SARS-CoV-1 patients. Focal myofiber necrosis was identified in 4 of 8 cases (50%). In situ hybridization for coronavirus was negative in all subjects. Potentially immune-mediated or critical illness myopathy and superimposed steroid myopathy. |
| Li | 2016 | Cohort study | No | China | 183 children with acute encephalitis (22 SARS1+) | 236 children with respiratory tract infection (26 SARS1+) | ELISA for IgM blood | Anti-SARS-CoV-1 IgM antibodies were detected in 22/183 (12%) of children with acute encephalitis. Headache, seizures and neck stiffness were common symptoms among SARS-CoV-1 positive children with encephalitis (10/22 [46%], 5/22 [23%] and 7/22 [32%], respectively). Serum granulocyte colony-stimulating factor (G-CSF) was significantly higher in CoV-CNS compared to healthy controls. Serum level of granulocyte macrophage colony-stimulating factor (GM-CSF) was significantly higher in CoV-CNS infection compared to CoV-respiratory tract infection. In patients with CoV-CNS infection, the levels of IL-6, IL-8, MCP-1, and GM-CSF were significantly higher in CSF compared to matched serum samples. 16 patients with neuroimaging: 8 showed abnormal findings - 2 children with seizures (1 CT, 1 MRI) with abnormity on temporal lobe; 2 children with headache with abnormity in periventricular region on MRI; 4 children (3 MRI, d1 CT) with abnormity in basal ganglia and thalamus. Complete recovery without sequelae of all children. |
| Sheng | 2005 | Cross-sectional study | No | China | 102 | N/A | Unclear | Questionnaires survey of 308 SARS patients after discharge from hospital. Among the 102 (33%) valid replies, 65% had strong neuropsychiatric complaints in convalescent phase as indicated by the general health questionnaire score ≥ 5. Pulse steroid and total dosages of pulse steroid during hospitalization were predictive for anxiety-depression, psychosis and behavioral symptoms during the acute phase. These effects persisted during the convalescent phase. Disease severity had direct correlation with symptoms in all neuropsychiatric domains during acute phase and anxiety-depression and cognition during convalescent phase. |
| Stainsby | 2011 | Case series | No | Canada | 3 | N/A | Unclear | Case 1: 38-year-old female with vision changes, bilateral peripheral neuropathy and sleeping difficulty 3 years after SARS-CoV-1 ; Case 2: 25-year-old female with sleeping difficulty and stumbling 1.5 years after SARS-CoV-1, normal brain MRI; Case 3: 39-year-old female with constant pain all over the body following a two-week isolation period in hospital. |
| Tang | 2004 | Retrospective study | Unclear | China | 173 SARS-CoV-1 patients with neuropsychiatric symptoms | N/A | Unclear | Approximately 53% of SARS-CoV-1 patients had neuropsychiatric symptoms. Most common symptoms were headache (67%), affective disorder (31%), dizziness (29%), anxiety (20%), reduced consciousness (10%), phobia (8%), depression (6%), mental disorder (5%), tendency of committing suicide (1%), seizures (1%) and focal neurological signs (0.6%). More severe clinical disease courses were more often associated with neuropsychiatric involvement. Ophthalmoscopic examination was performed for four patients and revealed that the optic disc outline was blurred and the retinal veins were congestive. Pathological characteristics of 2 patients: edema of the perivascular tissue and vascular wall of the small veins accompanied by mononuclear cells and lymphocytes infiltration, neurodegeneration and demyelination in the brain. |
| Tsai | 2004 | Case series | No | China | 4 | N/A | Serum corona-virus antibodies. | 4 patients (31 - 48-years-old) developed hyporeflexia/limb weakness 3 weeks after the onset of SARS-CoV-1. Two women experienced motor-predominant peripheral nerve disorders. One woman suffered neuropathy. One man developed myopathy. CSF in 2 patients normal (and negative for SARS-CoV-2). Both patients with myopathy had elevated serum creatine kinase levels. Clinical presentations were suggestive of critical illness neuro-/myopathy, DD Guillain-Barré syndrome |
| Umapathi | 2004 | Prospective cohort study | Yes | Singapore | 206 SARS, 5 with large artery stroke | N/A | Unclear | Out of 206 patients with SARS-CoV-1 in Singapore, 5 (2.4%) suffered large artery cerebral infarctions. Patients were 39 - 69-years-old, only one had stroke risk factors (diabetes, hypertension, ischemic heart disease). Brain CTs with apparent strokes. |
| Wei | 2009 | Case-control | No | China | 5 | 5 | Unclear | Autopsy of adenohypophysis from 5 SARS-CoV-1 patients (and 5 controls). Number of cells and staining intensity for growth hormone, TSH and adrenocorticotrophic hormone decreased, whereas that of PRL, FSH, and LH were increased in all included SARS-CoV-1 cases. |
| Xu | 2005 | Case report | No | China | 1 | N/A | RT-PCR from brain, ELISA in serum | A 39-year-old male with progressive CNS symptoms including dysphoria, vomiting and delirium 26 days after symptom onset. A brain CT revealed broad encephalic pathological changes of probably ischemia and necrosis and brain edema. Neuropathologic examination of the brain tissue revealed necrosis of neuron cells and broad hyperplasia of glia cells. |

*Abbreviations: CoV = coronavirus; ISH, in situ hybridization; NPH = nasopharyngeal; OR, odds ratio; RT-PCR, reverse transcriptase polymerase chain reaction; SARS = severe acute upper respiratory syndrome*

**Table S7**: Summary of studies assessing neurological manifestations and complication in SARS-CoV-2 infections.

| **First author** | **Study type** | **Multi-center** | **Country** | **Number of patients** | **Controls** | **Corona-proof** | **Summary findings** |
| --- | --- | --- | --- | --- | --- | --- | --- |
| Abdel-Mannan | Case series | No | UK | 8 children out of 27 children with severe COVID-19 | N/A | RT-PCR NPH swab or IgG blood | 4 out of 27 children (15%) with multisystem inflammatory syndrome developed neurological symptoms, among them encephalopathy, headaches, brainstem/cerebellar signs, muscle weakness and reduced reflexes. MRI showed splenial signal changes in all 4 patients (suggestive for ongoing [para-]infectious process). CSF from 2 children was acellular and without evidence for infection. All patients showed some degree of recovery by the end of the study, 2 children recovered completely. |
| Abdelnour | Case report | No | Ireland | 1 | N/A | Unclear | 69-year-old male who developed peripheral neuropathy during COVID-19. |
| Abdi | Case report | No | Iran | 1 | N/A | RT-PCR NPH swab | 58-year-old male with COVID-19 and ADEM. Brain MRI showed diffuse confluent white matter hyperintensity on FLAIR MRI without prominent enhancement on T1-weighted images. CSF was negative for SARS-CoV-2 RNA. |
| Acharya | Case report | No | USA | 1 | N/A | RT-PCR | 60-year-old male with acute central retinal artery occlusion. Brain CT scan normal. |
| Afshar | Case report | No | Iran | 1 | N/A | RT-PCR NPH swab | 39-year-old female with self-limited generalized tonic-clonic seizure on day 11 of hospital admission. FLAIR MRI with high signal intensities in bilateral thalami, medial temporal and pons. No gadolinium-enhancement. CSF RT-PCR for SARS-CoV-2 negative. |
| Agarwal | Case series | No | India | 2 | N/A | RT-PCR NPH swab | 56-year-old male with intracerebral hemorrhage in the pons on a CT scan. 72-year-old male with diabetes and sudden left-sided weakness. Brain MRI showed microbleeds in bilateral subcortical white matter zones of cerebral hemispheres, left basal ganglia, thalamus, pons and cerebellum. |
| Agosti | Case report | No | Italy | 1 | N/A | RT-PCR NPH swab | 68-year-old male with acute inflammatory demyelinating polyradiculoneuropathy (AIDP) subtype of Guillain-Barré syndrome (GBS). |
| Alberti | Case report | No | Italy | 1 | N/A | RT-PCR NPH swab | A 71-year-old male with tetraparesis, suspect GBS. |
| Al Ketbi | Case report | No | United Arab Emirates | 1 | N/A | RT-PCR NPH swab | 32-year-old patient with sudden onset of paraplegia and urinary retention. Spinal MRI with extensive diffuse hyperintense signal in the grey matter of the cervical, dorsal, and lumbar spinal cord. Cervical spinal cord with mild swelling. |
| Al Saiegh | Case series | No | USA | 2 | N/A | RT-PCR NPH swab | A 31-year-old male with subarachnoid hemorrhage and a 62-year-old female with cerebral infarction. |
| Al Olama | Case report | No | United Arab Emirates | 1 | N/A | RT-PCR NPH swab | 36-year-old male with drowsiness showed CT signs suggestive for meningoencephalitis, complicated by intracerebral hematoma and subdural hematoma. CSF was positive for SARS-CoV-2 RNA. |
| Ameres | Case series | No | Switzerland | 28 | 72 | RT-PCR NPH swab | SARS-CoV-2 status is an independent predictor of serum neurofilament levels. |
| Andrea | Case series | No | Italy | 2 | N/A | RT-PCR NPH swab | 2 patients with GBS: one of them with Miller-Fisher syndrome overlap, one of them with acute motor sensory axonal neuropathy with markedly impaired consciousness. CSF negative for SARS-CoV-2 RNA. Brain MRI from 1 patient normal. |
| Andriuta | Case series | No | France | 2 | N/A | RT-PCR NPH swab | Two middle-aged patients with encephalopathy. CSF for SARS-CoV-2 RNA negative. Non-contrast-enhanced brain MRI showed diffuse brain hyperintensities with a normal apparent diffusion coefficient. |
| Anzalone | Case series | No | Italy | 4 | N/A | Unclear | Four patients with subacute encephalopathy with multifocal cortical involvement. Lesions were T2 hyperintense and were located in the parietal, occipital and frontal regions. On diffusion-weighted MRI, almost all lesions were characterized by the absence of apparent diffusion coefficient changes. Susceptibility-weighted imaging was normal. One patient with a follow-up MRI after 1 month showed complete resolution of all lesions. |
| Aragao | Case series | No | Brazil | 5 | N/A | Unclear | 1 patient with MRI suggestive of microbleeding in the olfactory bulb. Also imaging findings from the other four patients could represent microbleeding in the olfactory bulb (albeit only post-contrast imaging available). |
| Araca | Case report | No | USA | 1 | N/A | Unclear | 58-year-old male with multiple sclerosis on fingolimod developed neck stiffness. Brain MRI with no acute findings. |
| Arnaud | Case report | No | France | 1 | N/A | RT-PCR NPH swab | 64-year-old male with GBS. Brain CT normal. CSF negative for SARS-CoV-2 RNA. |
| Ashrafi | Case series | No | Iran | 6 | N/A | RT-PCR NPH swab | 6 patients under 55 years with stroke. Most commonly affected brain area was the middle cerebral artery territory (five patients). |
| Atere | Case report | No | USA | 1 | N/A | Unclear | 46-year-old male with syncope and seizure. Brain CT and MRI normal. CSF positive for SARS-CoV-2 RNA. |
| Avula | Case series | No | USA | 4 | N/A | RT-PCR, unclear site | 4 patients older than 70 with large artery cerebral infarction. |
| Balestrino | Case report | No | Italy | 1 | N/A | Unclear | 73-year-old male with ataxia and reduced consciousness. Brain CT normal. No CSF sample obtained. |
| Balloy | Case report | No | France | 1 | N/A | RT-PCR tracheal swab | A 59-year-old male with fever, dyspnea and headache. Due to hypoxia in need of intubation. After extubation confused, EEG suggested epileptic seizures. |
| Bao | Case report | No | China | 1 | N/A | RT-PCR NPH swab | 38-year-old male with large intracerebral hemorrhage in the basal ganglia. CSF negative for SARS-CoV-2 RNA. |
| Barrios-Lopez | Case series | No | Spain | 4 | N/A | Unclear | 4 patients with ischemic stroke, causal relationship between COVID-19 and stroke likely in 2 of these patients. |
| Beach | Case series | No | USA | 4 | N/A | RT-PCR NPH swab | 4 COVID-19 cases with prior cognitive decline presented with delirium. 3/4 cases (75%) lacked significant respiratory symptoms. |
| Beltran-Corbellini | Case-control | Yes | Spain | 97 | 40 (Influenza) | RT-PCR NPH swab | Study aimed at determining whether new-onset smell/taste disorders are more common among COVID-19 patients than influenza patients. New-onset smell/taste disorders more frequent among cases (31/97, 39%) compared to controls (5/40, 13%). Among the COVID-19 patients with smell/taste disorder, 25/31 (81%) presented smell disorders and 28/31 (90%) taste disorders. Only four patients reported concomitant NPH obstruction. The disorder was with an acute onset in 22/31 patients (71%) and was initial manifestation in 11/31 patients (35.5%). |
| Belvis | Case report | No | Spain | 1 | N/A | RT-PCR NPH swab | 51-year-old male with headache. |
| Benameur | Case series | No | USA | 3 | N/A | unclear | 3 patients with encephalopathy/encephalitis. MRI showed non-contrast-enhancing unilateral, bilateral, and midline changes not readily attributable to vascular causes. CSF negative for SARS-CoV-2 RNA in all cases. |
| Benger | Retrospective case series | 2020 | UK | 5 | N/A | RT-PCR NPH swab | 5 patients with intracerebral hemorrhage, most of the patients had known cardiovascular risk factors. |
| Benussi | Retrospective cohort study | 2020 | Italy | 56 COVID-19 patients | 117 SARS-CoV-2 negative patients | RT-PCR respiratory tract | Patients on Neuro-COVID19 ward. Patients and controls had similar baseline characteristics, but COVID-19 patients had more severe strokes and worse outcomes compared to non-COVID-19 patients. |
| Bernard-Valnet | Case series | No | Switzerland | 2 | N/A | RT-PCR NPH swab | A 64-year-old with acute psychotic symptoms and epileptic findings on EEG, with CSF suggesting viral meningoencephalitis. A 67-year old female with confusion, sensory neglect and hemianopia, CSF showing lymphocytic pleocytosis. MRI without pathological findings in both patients. |
| Beyrouti | Case series | No | UK | 6 | N/A | One with RT-PCR NPH swab, the rest symptomatic+ chest CT | 6 patients older than 50 with acute large vessel cerebral infarction. |
| Bhatta | Case report | No | USA | 1 child | N/A | RT-PCR unclear site | 11-year old boy with new-onset seizure. No CSF sample obtained. |
| Bigaut | Case series | No | France | 1 | N/A | RT-PCR | 2 patients with GBS. CSF negative for SARS-CoV-2 RNA. MRI from 1 patient with radiculitis and plexitis on both brachial and lumbar plexus as well as multiple cranial neuritis. |
| Bodro | Case series | No | Spain | 2 | N/A | RT-PCR NPH swab | 2 patients with encephalitis (one of them being 25 years old). CSF negative for SARS-CoV-2 RNA. Unremarkable brain MRI and CT scans. Increased CSF levels of IL-1β, IL-6, and ACE. |
| Bonardel | Case report | No | France | 1 | N/A | RT-PCR NPH swab | 51-year-old diabetic patient with bilateral temporo-occipital stroke with clinical presentation of sudden cortical blindness. Hemorrhagic transformation after intravenous thrombolysis. No CSF sample obtained. |
| Bracaglia | Case report | No | France | 1 | N/A | RT-PCR NPH swab | 66-year-old female with GBS, otherwise completely asymptomatic. |
| Bruggemann | Case report | No | The Netherlands | 1 | N/A | RT-PCR | 57-year-old male with arterial and venous thromboembolisms and subsequent hemiplegia due to ischemic stroke in the right frontal lobe (confirmed by brain CT scan). |
| Brun | Case report | No | France | 1 | N/A | RT-PCR NPH swab | 54-year-old female with altered mental status and hemiplegia. Brain MRI showed restricted diffusion with homogenous gadolinium-enhancement, suggestive of demyelination. CSF RT-PCR for SARS-CoV-2 RNA was negative twice. |
| Butt | Case report | No | UK | 1 | N/A | RT-PCR NPH swab | Confusion as first manifestation of COVID-19. Delirium still unchanged 4 weeks after admission. Normal brain CT. |
| Caamano | Case report | No | Spain | 1 | N/A | RT-PCR NPH swab | 61-year-old male with GBS. CSF negative for SARS-COV-2 RNA. Brain CT and MRI without acute pathology. |
| Cam-dessanche | Case series | No | France | 2 | N/A | RT-PCR NPH swab | 2 patients who developed GBS during the course of COVID-19. CSF from one patient, negative for SARS-CoV-2 RNA. |
| Cantador | Retrospective cohort study | No | Spain | 1419 | N/A | Unclear | Out of 1419 patients, 8 patients had a cerebrovascular event (0.6%): 6 with an acute ischemic stroke and 2 a transient ischemic attack. |
| Capelli | Retrospective case series | No | Italy | 27 | N/A | Unclear | 27 patients with COVID-19 and smell disorders. Increased incidence of anosmia compared to similar periods of recent years. |
| Carfi | Cohort study | No | Italy | 143 | N/A | RT-PCR NPH swab | Patients were assessed for a mean of 60 days after onset of the first COVID-19 symptoms. 76 patients (53%) still reported fatigue at this time point. |
| Carignan | Case-control study | No | Canada | 134 | N/A | RT-PCR | Smell and taste disorders were independently associated with COVID-19. |
| Carroll | Case series | No | USA | 2 | N/A | RT-PCR NPH swab | 2 critically ill patients with massive intracerebral hemorrhage. |
| Cavalcanti | Case series | No | USA | 3 | N/A | RT-PCR NPH swab | 3 patients under 43 years old with cerebral venous thrombosis. 2 of them with hemorrhagic venous infarcts. All patients with fatal outcome. |
| Cebrian | Case report | No | Spain | 1 | N/A | RT-PCR NPH swab | 74-year-old male with headache and impaired consciousness. CSF positive for SARS-CoV-2 RNA in the absence of inflammatory CSF. MRI with area of restricted diffusion. |
| Cecchetti | Consecutive case series | No | Italy | 18 | N/A | RT-PCR NPH swab | 18 patients with altered background EEG activity, based on a three-grade scale [normal/mild (5 patients), moderate (9 patients) or severe (4 patients)]. |
| Cerasti | Case report | No | Italy | 1 | N/A | Unclear | 47-year-old immunocompromised female with multiple strokes on brain CT. |
| Chan | Case report | No | Canada | 1 | N/A | RT-PCR oropharyngeal swab | 58-year-old patient with GBS. CSF negative for SARS-CoV-2 RNA. Brain MRI with bilateral facial nerve enhancement. |
| Chao | Case report | No | China | 1 | N/A | Unclear | A 51-year-old female, 12 days after intubation severe weakness and numbness in both legs. 10 days after extubation: normal mental state, normal cranial nerves, symmetric weakness in lower limbs, stocking distribution of paresthesia, decreased nerve amplitudes, normal spinal MRI, CSF sampling refused. 2 months later only minimal remaining weakness. Presentation suggestive for critical illness polyneuropathy. |
| Chaumont | Case report | No | Carribean France | 1 | N/A | RT-PCR NPH swab | 69-year-old male with headache, neck stiffness and altered consciousness suggestive of meningoencephalitis. Lymphocytic CSF (but negative for SARS-CoV-2 RNA). Brain MRI with gadolinium was normal. |
| Chaumont | Case series | No | Carribean France | 4 | N/A | RT-PCR NPH swab | 4 patients with the combination of central and peripheral nervous system disorders. Symptoms occurring unexpectedly late after the first symptoms (after mechanical ventilation weaning). 1 of these patients with small subcortical stroke, otherwise normal MRI. CSF negative for SARS-CoV-2 RNA in all patients. |
| Chen | Retrospective case series | No | China | 113 deceased patients | 161 recovered patients | RT-PCR NPH swab | Among a cohort of 799 COVID-19 patients, 113 deceased and 161 recovered patients were assessed for their symptomatology. Common complications in deceased patients were decreased consciousness (22% in deceased, 1% in recovered) and hypoxic encephalopathy (20% in deceased, 1% in recovered). Neurological symptoms included fatigue (57% in deceased, 45% in recovered), headache (10% in deceased, 12% in recovered) and dizziness (9% in deceased, 7% in recovered). |
| Chen | Retrospective case series | No | China | 99 | N/A | RT-PCR NPH swab | Study aimed at clarifying clinical characteristics of COVID-19. Neurological symptoms are confusion (9/99 patients, 9%) and headache (8/99 patients, 8%). |
| Chen | Consecutive case series | Yes | USA | 102 | N/A | Unclear | 24/102 COVID-19 patients (24%) presented with syncope/presyncope or mechanical falls. |
| Chen | Case series | No | China | 3 | N/A | RT-PCR NPH and throat swab | 3 18- and 27-year-old patients with anosmia. Brain CT scans were normal. |
| Chiesa | Prospective survey-based data | Yes | Spain | 751 | N/A | RT-PCR NPH swab | 621 patients (83%) who reported a total loss of smell and 130 (17%) with partial loss. Even after almost 7 weeks, 277 (37%) of patients reported persistent loss of smell, 107 (14%) reported partial recovery and 367 (49%) reported complete recovery. |
| Chougar | Case report | No | France | 1 | N/A | RT-PCR NPH swab | 72-year-old male with deep cerebral vein thrombosis complicated with hemorrhagic venous infarction |
| Chougar | Retrospective case series | No | France | 73 | N/A | RT-PCR NPH swab | Out of 73 patients with neurological symptoms, 43 had abnormal brain MRI (59%): 22 patients with abnormal perfusion (48%), 17 patients with ischemic stroke (23%), 8 patients with multiple microbleeds (11%), 3 patients with restricted diffusion within the corpus callosum (cytotoxic lesions of the corpus callosum) and 1 patient with a deep venous thrombosis (1%). Imaging findings associated to COVID-19 were multifocal white matter enhancing lesions (4 patients, 5%) and basal ganglia abnormalities (4 patients, 5%) |
| Co | Case report | No | Philippines | 1 | N/A | RT-PCR NPH swab | 62-year-old female with ischemic stroke. |
| Coen | Case report | No | Switzerland | 1 | N/A | RT-PCR NPH swab | Male patient in his 70s without prior medical history developed GBS. CSF was negative for SARS-CoV-2 RNA. Contrast-enhanced brain MRI was normal. |
| Coolen | Case series | No | Belgium | 19 non-survivors with post-mortem brain MRI | N/A | RT-PCR NPH swab | 4 decedents with intracranial vasculopathy: subcortical micro- and macro-bleeds (2 cases), cortico-subcortical edematous changes suggestive for posterior reversible encephalopathy syndrome (PRES, one case) and nonspecific deep white matter changes (one case). 4 decedents with asymmetric olfactory bulbs (but without downstream olfactory tract abnormalities). |
| Craen | Case report | No | USA | 1 | N/A | RT-PCR unclear site | A 66-year-old female with hypertension, diabetes mellitus type 2 and hyperlipidemia. Cardiac arrest followed by subarachnoid hemorrhage. |
| D’Anna | Consecutive case series | No | UK | 8 | N/A | Unclear | 7 patients with ischemic stroke, 1 patient with hemorrhagic stroke. |
| Dakay | Case report | No | USA | 1 | N/A | RT-PCR NPH swab | Female patient with cervical vertebral artery dissection combined with subarachnoid hemorrhage due to reversible cerebral vasoconstriction syndrome. |
| De Freitas | Case report | No | Brazil | 1 | N/A | RT-PCR NPH swab | 39-year-old immunocompetent male with mild COVID-19 developed left facial herpes zoster affecting the  trigeminal nerve. Brain MRI with trigeminal nerve enhancement. |
| Deliwala | Case report | No | USA | 1 | N/A | Unclear | 31-year-old female without cardiovascular risk factors and cortical stroke in the right middle cerebral artery territory. |
| Demirci Otluoglu | Case report | No | Turkey | 1 | N/A |  | 48-year-old male with encephalomyelitis with acute lesions on brain and spinal MRI. positive for SARS-CoV-2 RNA. |
| De Stefano | Case report | No | Switzerland | 1 | N/A | RT-PCR NPH swab | 56-year-old women with altered mental status and EEG findings suggestive for focal dysfunction. MRI with multiple microbleeds located bilaterally in the white matter junction, various regions of the corpus callosum and internal capsule, suggestive of critical illness-associated cerebral microbleeds. CSF negative for SARS-CoV-2 RNA. |
| Diaz-Segarra | Case series | No | USA | 4 | N/A | Unclear | Four cases with ischemic strokes. |
| Dixon | Case report | No | UK | 1 | N/A | RT-PCR NPH swab | 59-year-old female with aplastic anemia with diffuse brain edema on CT scan. MRI demonstrated symmetrical hemorrhagic lesions in the brain stem, amygdalae, putamina, and thalamic nuclei suggestive of hemorrhagic acute necrotizing encephalopathy. CSF negative for SARS-CoV-2 RNA. |
| Dogan | Case series | No | Turkey | 6 | N/A | RT-PCR unclear site | 6 ICU-patients which after extubation had either agitated delirium or failed to regain consciousness. MRI showed suspect encephalitis in 3 of them. |
| Dogra | Retrospective case series | No | USA | 33 with intracranial hemorrhage | N/A | RT-PCR NPH swab | 33 patients with intracranial hemorrhage: 5 patients with parenchymal hemorrhages with mass effect, 7 patients with punctate hemorrhages, 17 with small-moderate sized hemorrhages and 4 patients with large single site hemorrhage. Almost all patients received anticoagulation prior to intracranial hemorrhages. |
| Domingues | Case report | No | Brazil | 1 | N/A | RT-PCR NPH swab | 42-year-old male with a clinical presentation compatible with clinically isolated syndrome. SARS-CoV-2 genome was detected and sequenced in CSF with 99.74–100% similarity between patient virus and worldwide sequences. |
| Dugue | Case report | No | USA | 1 neonate | N/A | RT-PCR NPH swab | 6-week-old infant with fever, cough, and episodes of upward gaze and bilateral leg stiffening suggestive of febrile seizure. EEG normal. CSF negative for SARS-CoV-2 RNA. |
| Dumitrascu | Case report | No | USA | 1 | N/A | RT-PCR NPH swab | 48-year-old male with ophthalmic artery occlusion during therapeutic anticoagulation with apixaban for deep venous thrombosis. Brain/neck/ophthalmic MRI normal. |
| Duong | Case report | No | USA | 1 | N/A | Unclear | 41-year-old female with signs of viral encephalitis. Brain CT without contrast agent war normal. No CSF analysis. |
| Edler | Case series | No | Germany | 80 autopsy cases | N/A | RT-PCR NPH swab | At least one case with signs of hypoxic brain damage in neuropathological assessment. |
| Efe | Case report | No | Turkey | 1 | N/A | Unclear | 35-year-old female with COVID-19-associated encephalitis mimicking glial tumor. Brain MRI compatible with high-grade glioma. Neuropathological assessment of operated brain tissue revealed lymphocytic perivascular infiltration suggestive for encephalitis. |
| Elkhouly | Case series | No | USA | 2 | N/A | RT-PCR unclear site | One case with GBS, one case with Bell’s palsy. |
| El Otmani | Case report | No | Morocco | 1 | N/A | RT-PCR oropharyngeal swab | A 70-year-old female with tetraplegia, hypotonia and areflexia. Suspected GBS. |
| Escalada Pellit | Case report | No | Spain | 1 | N/A | RT-PCR unclear site | 30-year-old female with headache, hypoacusis and tinnitus suggestive of vestibular dysfunction. Normal brain MRI. |
| Escalard | Consecutive case series | No | France | 10 patients with ischemic stroke | N/A | RT-PCR unclear site | 10 patients with ischemic stroke. Median time from COVID-19 symptoms to stroke onset was 6 days. 6 of these patients died. |
| Espinosa | Case report | No | USA | 1 | N/A | RT-PCR unclear site | A 72-year-old male with hypertension, diabetes mellitus type 2 and hyperlipidemia. Went into shock. MRI showed new lacunar infarction in the parietal region, EEG showed encephalopathy. |
| Espindola | Case series | No | Brazil | 8 | N/A | RT-PCR NPH swab | COVID-19 patients with distinct neurological disorders such as meningoencephalitis, facial palsy, consciousness disorder or headache have undetectable or extremely low levels of SARS-CoV-2 RNA in the CSF. |
| Falcone | Case report | No | USA | 1 | N/A | Unclear | 32-year-old male with abducens nerve palsy. MRI with signs of left lateral rectus muscle atrophy. |
| Fara | Case report | No | USA | 1 | N/A | RT-PCR unclear site | 3 patients with mild COVID-19 had a stroke secondary to large vessel thrombosis without occlusion. |
| Farhadian | Case report | No | USA | 1 | 3 | RT-PCR NPH swab | 78-year-old immunocompromised female with seizure-like activity. MRI without acute pathological findings. CSF with increase in inflammatory cytokines such as IL-6, IL-8, IP-10 and MCP-1 (compared to 3 controls). |
| Farzi | Case report | No | Iran | 1 | N/A | RT-PCR NPH swab | 41-year-old male with GBS (AIDP type). Intravenous immunoglobulin with favorable effects. No MRI or CSF sample obtained. |
| Fasano | Case report | No | Italy | 1 | N/A | RT-PCR NPH swab | 54-year-old male with motor seizure as presenting symptom of COVID-19. Brain CT normal. No brain MRI or CSF sample obtained. |
| Faucher | Case report | No | France | 1 | N/A | RT-PCR unclear site | 21-year-old male with sudden onset binocular horizontal diplopia. Oculomotor nerve palsy. |
| Fernandez-Dominguez | Case report | No | Spain | 1 | N/A | Unclear | 74-year-old female with Miller-Fisher-like syndrome. Brain MRI without acute pathologies. CSF negative for SARS-CoV-2 RNA. |
| Filatov | Case report | No | USA | 1 | N/A | RT-PCR NPH swab | A 74-year-old male with cardiovascular risk factors. Quick exacerbation of respiratory symptoms and mental status alteration. Brain CT normal (except old stroke). EEG with bilateral slowing and focal slowing in the left temporal region with sharply contoured waves. CSF without signs of infection. Findings suggestive for encephalopathy. Patient remained in ill condition. |
| Finatti | Case series | No | Italy | 3 | N/A | Unclear | 3 30-40-year-old patients without history of psychiatric illness who were admitted to a psychiatric ward with psychotic symptoms. |
| Fischer | Case report | No | USA | 1 | N/A | Unclear | 47-year-old male with severe COVID-19 and prolonged unresponsiveness demonstrated intact functional network connectivity in functional MRI. He recovered weeks later with the ability to follow commands. |
| Fitsiori | Case series | No | Switzerland | 9 | N/A | RT-PCR NPH swab | 9 patients with moderate to severe COVID-19. Common MRI findings were the presence of microbleeds in an unusual distribution, i.e. in the corpus callosum. Other less frequent locations of microbleeds were the internal capsule (5 patients) and middle cerebellar peduncles (5 patients). In the majority of patients, subcortical regions were also affected. |
| Franceschi | Case series | No | USA | 2 | N/A | Unclear | 2 patients with hemorrhagic PRES. Brain CT without contrast agent showed focal edema in the posterior parietooccipital regions bilaterally and with a small right-sided hemorrhage. MRI susceptibility-weighted images showed extensive petechial hemorrhages diffusely distributed throughout the corpus callosum or extensive intracerebral hemorrhages as well as cortical patchy contrast-enhancement. |
| Franceschi | Case series | No | USA | 10 | N/A | RT-PCR unclear site | 10 patients with severe neurovascular complications, mostly ischemic. Most patients had cardiovascular risk factors. |
| Frank | Case report | No | Brazil | 1 child | N/A | RT-PCR NPH swab | 15-year-old male presenting with frontal headaches with retro-orbital pain who developed GBS. CNS MRI unremarkable. CSF negative for SARS-CoV-2 RNA. |
| Freni | Prospective case series | No | Italy | 50 | N/A | RT-PCR NPH swab | 46/50 patients (92%) had smell disorders related to COVID-19. 35/50 patients (70%) reported taste disorders. |
| Gane | Case series | No | UK | 12 | N/A | Unclear | 12 patients with isolated sudden onset anosmia. |
| Galanopoulou | Retrospective case series | No | USA | 22 | 6 | RT-PCR NPH swab | Most common indications for EEG in COVID-19 patients versus controls were new onset encephalopathy (68% vs 33%) and seizure-like events (64% vs 33%), even in patients without prior history of seizures. Sporadic epileptiform discharges were present in 41% of COVID-19 patients. |
| Garaci | Case report | No | Italy | 1 | N/A | RT-PCR NPH swab | 44-year-old female with negative past medical history. Cerebral CT angiography showed venous thrombosis in the vein of Galen, straight sinus and in the confluence sinus. No brain MRI was obtained. |
| Galougahi | Case report | No | Iran | 1 | N/A | RT-PCR NPH swab | Sudden onset of a complete loss of olfactory function. Olfactory bulb MRI showed normal olfactory bulb volume without abnormal signal intensity and no sign of nasopharyngeal congestion. |
| Gautier | Case report | No | Switzerland | 1 | N/A | Unclear | One of the authors of this study had a rapid onset of smell and taste disorders as well as headache upon presumable COVID-19. |
| Ghiasvand | Case report | No | Iran | 1 | N/A | RT-PCR upper respiratory tract | 68-year-old female with symmetric polyneuropathy. No CSF sample obtained. |
| Ghosh | Case report | No | Canada | 1 | N/A | RT-PCR NPH swab | 19-year-old female with thalamic hemorrhage and acute cognitive impairment unmasking Moyamoya angiopathy. No CSF or MRI obtained. |
| Gilani | Case series | No | Iran | 8 | N/A | Unclear | 8 (22 - 44 year old) patients with anosmia and/or ageusia starting within a couple of days after respiratory symptom/fever onset (3 of them were not tested for SARS-CoV-2) |
| Gill | Case report | No | USA | 1 | N/A | RT-PCR NPH swab | 79-year-old female with cardiovascular risk factors presented with ischemic stroke. CT angiography showed partial right M2, M3 and parietal segment occlusions. Brain MRI with multiple small peripheral infarctions. |
| Giorganni | Case report | No | Italy | 1 | N/A | RT-PCR NPH swab | 22-year-old female with diabetes mellitus type 1 who developed loss of consciousness. Brain CT and CT-angiography showed small right frontal parenchymal hemorrhage. Subsequently, she developed flaccid tetraparesis. CSF normal. No MRI obtained. |
| Goh | Case report | No | Singapore | 1 | N/A | RT-PCR NPH swab | 27-year-old male with facial nerve palsy. Brain MRI showed enhancement of the left facial nerve. CSF negative for SARS-CoV-RNA. |
| Goldberg | Case report | No | USA | 1 | N/A | RT-PCR NPH swab | A 64-year-old male with hemiparesis and acute hypoxemic respiratory failure. Acute cerebral infarction of the area corresponding with the right middle cerebral artery. Emboli in the right internal carotid artery. |
| Gomez-Iglesias | Online observational study | No | Spain | 909 | N/A | Unclear | 91% of COVID-19 patients reported simultaneous smell and taste disorder. 54% did not report concomitant nasal congestion or mucus. |
| González-Pinto | Case report | No | Spain | 1 | N/A | RT-PCR unclear site | A 36-year-old female. Global aphasia and right hemiplegia. Chest CT shows bilateral pulmonary thromboembolism. Head-neck CT-angiography with thrombi in ascending aorta, occluded left internal carotid, middle cerebral artery and anterior cerebral artery. |
| Gulko | Case series | No | USA | 2 | N/A | Unclear | 2 patients with ischemic stroke and carotid artery thrombi. |
| Guillan | Case report | No | Spain | 1 | N/A | RT-PCR NPH swab | 67-year-old male with hypertension develops ischemic stroke. Brain CT with bilateral parietooccipital and right cerebellar hypoattenuating lesions with areas of cortical hyperattenuating involvement. Brain MRI confirmed stroke. |
| Gutiérrez-Ortiz | Case series | No | Spain | 2 | N/A | RT-PCR NPH swab and CSF | Case 1: 50-year old male with anosmia, ageusia, right internuclear ophthalmoparesis, right fascicular oculomotor palsy, ataxia and areflexia 3 days after onset of respiratory symptoms. CSF with GD1b-IgG. Brain CT normal. Presentation suggestive for Miller-Fisher syndrome. Case 2: 39-year-old male with ageusia, bilateral abducens palsy and areflexia after 3 days of low-grade fever and diarrhea. Brain CT normal. Clinical presentation suggestive for polyneuritis cranialis. RT-PCR positive from NPH swab positive but negative for CSF in both patients. |
| Haddad | Case report | No | USA | 1 | N/A | Unclear | 41-year-old male with history of well-controlled HIV presented with confusion. Patient developed encephalopathy with tonic-clonic seizure. Brain MRI unremarkable. |
| Haddadi | Case report | No | Iran | 1 | N/A | Negative RT-PCR, diagnosed based on chest CT- | A 54-year-old female with hypertension and diabetes mellitus type 2. GCS suddenly 10. MRI and brain CT showing bilateral hemorrhagic insults in the basal ganglia. |
| Haldrup | Case report | No | Denmark | 1 | N/A | Unclear | Patient with the only COVID-19 symptom being a sudden onset of persistent smell and taste disorder. |
| Hanafi | Case report | No | USA | 1 | N/A | RT-PCR NPH swab | 65-year-old male with extensive cerebral small-vessel ischemic lesions suggestive of cerebral vasculitis. Brain MRI with combined imaging pattern of ischemia, hemorrhage, and punctuate postcontrast enhancement. |
| Hayashi | Case report | No | Japan | 1 | N/A | RT-PCR throat swab | 75-year-old male with Alzheimer’s disease developed mild ataxic gate. Brain MRI showed hyperintense lesion in the splenium of the corpus callosum on diffusion-weighted images suggestive for encephalitis/encephalopathy with a reversible splenial lesion (MERS). |
| Helbok | Case report | No | Austria | 1 | N/A | RT-PCR oropharyngeal swab (negative) and IgG/IgM in serum and CSF | 68-year-old male with GBS. Spinal MRI unremarkable. |
| Heman-Ackah | Case series | No | USA | 2 | N/A | RT-PCR unclear site | 2 patients with massive intracerebral hemorrhage and midline shift on brain CT scan. |
| Helms | Observational case series | No | France | 58 | N/A | RT-PCR NPH swab and CSF (in 7 patients) | 64 consecutive ICU patients, 58 included. Most common neurological symptoms were confusion (65%), agitation (69%), corticospinal tract signs (67%) and dysexecutive syndrome (36%). 13 patients underwent brain MRI: 8/13 patients had leptomeningeal enhancement (62%), 11/11 with frontotemporal perfusion abnormalities (100%), 3/13 with cerebral stroke, 1 subacute/old (23%). Seven patients with prior neurological disorders (TIA, partial epilepsy, mild cognitive impairment). CSF analysis in 7 patients: oligoclonal bands in 2/7 (29%), elevated CSF IgG and protein levels in 1/7 (14%). All 7 patients were negative for SARS-CoV-2 RNA in CSF. |
| Hemasian | Case report | No | Iran | 1 | N/A | RT-PCR unclear site | 65-year-old male without prior medical history with hemorrhagic infarct in the right temporal lobe and right sigmoid and transverse sinus thrombosis. |
| Hepburn | Case series | No | USA | 2 | N/A | RT-PCR NPH swab | 2 patients with acute encephalopathy with clinical  and EEG documented seizures. Spinal MRI in 1 patient with epidural abscess. |
| Hernandez-Fernandez | Retrospective case series | No | Spain | 23 patients with stroke or intracranial hemorrhage (out of 1683 SARS-CoV-2 admissions) | N/A | RT-PCR NPH swab and/or chest CT semiology | Out of 1683 COVID-19 admissions, 23 patients (1.4%) developed cerebrovascular disease; 17 of these were diagnosed with ischemic stroke (74%, two with arterial dissections), 5 with intracerebral hemorrhage (22%, some patients with additional subarachnoid hemorrhages and/or microbleeds) and 1 with leukoencephalopathy. Ischemic strokes were common in the vertebrobasilar territory. |
| Hjelmesæth | Case series | No | Norway | 2 | N/A | RT-PCR unclear site | A man in his 90s that was in contact with his relatives and they both developed anosmia and ageusia 7-10 days after. Both RT-PCR confirmed. |
| Homma | Case report | No | Japan | 1 | N/A | RT-PCR NPH swab | 35-year-old female with facial nerve palsy. CSF negative for SARS-CoV-2 RNA. |
| Hopkins | Observational study | No | UK | 382 | N/A | Self-reported anosmia, no clear evidence | 80% reported lower severity score of anosmia one week after the initial survey, particularly those who reported anosmia longer than 2 weeks before the initial survey, |
| Hopkins | Survey based cohort study | Unclear | UK | 2428 | N/A | RT-PCR NPH swab for most respondents | 17% of patients did not report any other symptom besides anosmia. |
| Hornuss | Case control study | No | Germany | 45 | 45 | RT-PCR NPH swab | 49% of patients reported dys-/anosmia versus 27% of control patients. |
| Hosseini | Case series | No | UK | 2 | N/A | RT-PCR NPH swab | 2 patients with delirium and MRI signs of encephalitis (diffusion restriction). CSF negative for SARS-CoV-2 RNA. |
| Huang | Prospective study | No | China | 41 | N/A | RT-PCR serum | Most common neurological symptoms were myalgia or fatigue (18/41, 44%), headache (3/38, 8%; 3/25, 12% for non-ICU patients). |
| Huang | Case report | No | China | 1 | N/A | RT-PCR NPH swab | 40-year-old woman with diabetes mellitus type 2 and encephalitis. CSF positive for SARS-CoV-2 RNA. |
| Hutchins | Case report | No | USA | 1 | N/A | RT-PCR NPH swab | 21-year-old male with bifacial weakness with paresthesia, GBS subtype. Brain MRI with abnormal enhancement of facial and abducens nerves bilaterally, as well as the right oculomotor nerve. |
| Immovilli | Retrospective case series | No | Italy | 19 | N/A | Unclear | 19 patients with ischemic stroke. Etiologies were large artery atherosclerosis in 4 cases, cardioembolism in 5 cases, small vessel disease in 2 cases, and undetermined in 6 cases. There was an association between stroke and pneumonia severity. |
| Jain | Retrospective cohort study | Yes | USA | 545 | N/A | RT-PCR NPH or oro-pharyngeal swab | 545/3218 (14%) of COVID-19 patients underwent neuroimaging. 38 of these patients (8%) had acute pathological findings in neuroimaging: large infarct - 17 patients, 45%; lacunar infarct - 9 patients, 24%; hemorrhagic stroke - 9 patients, 24%; encephalitis – 1 patient, 2.5%; hypoxic ischemia – 2 patients, 5%. In total, 1.1% of hospitalized patients developed a stroke. |
| Joob | Retrospective cohort study | Yes | Thailand, China | 272 | N/A | Unclear | Only 1/272 had confusion as initial manifestation of COVID-19. |
| Kadono | Case report | No | Japan | 1 | N/A | RT-PCR NPH swab | 44-year-old male with known symptomatic epilepsy due to cerebral venous thrombosis. Upon COVID-19, he developed brain edema on brain CT with additional seizures and subsequent smell disorder. |
| Kanberg | Cohort study | No | Sweden | 47 | 33 | RT-PCR NPH swab | Patients were grouped into mild, moderate and severe COVID-19. Patients with severe COVID-19 had higher plasma concentrations of GFAP and neurofilament light chain compared to controls. GFAP was also increased in patients with moderate disease. In severe patients, an early peak in plasma GFAP decreased upon follow-up. Neurofilament light chain showed a persistent increase from first to last follow-up, possibly reflecting a sequence of early astrocytic response and more delayed axonal injury. |
| Kandemirli | Retrospective case series | Yes | Turkey | 27 | N/A | Unclear | 12 of 27 patients (48%) had acute pathological brain MRI findings (only included those who had COVID-19 and neurological symptoms, in need of ICU-treatment and who had an MRI). 10 had cortical T2-weighted FLAIR hyperintensities, 3 of those also had white matter hyperintensities. 1 had a thrombosis in the transverse sinus and 1 had an acute infarction in the right cerebral artery territory. |
| Karadas | Retrospective cohort study | No | Turkey | 239 | N/A | RT-PCR NPH swab | 83 of 239 patients (35%) had neurological symptoms. Most common neurological finding was headache (28%). D-dimer blood levels were higher in patients with at least one neurological symptom compared to patients without neurological symptom. |
| Karimi | Case report | No | Iran | 1 | N/A | RT-PCR respiratory tract | 30-year-old female with repeated generalized tonic-clonic seizures. Brain MRI normal. |
| Karimi-Galougah | Case report | No | Iran | 1 | N/A | RT-PCR unclear site | A 27-year-old female with anosmia. ^18^FDG PET/CT suggests hypometabolism of the left orbitofrontal cortex, corresponding with the olfactory bulb. |
| Kaya | Case report | No | Turkey | 1 | N/A | RT-PCR NPH swab | A 38-year-old male with confusion and high blood pressure. Visual loss in both eyes. MRI shows changes similar to PRES. |
| Khan | Case series | No | United Arab Emirates | 22 | N/A | RT-PCR unclear site | All 22 patients had large territory infarctions, most having evidence of vessel occlusion on CT angiography. 7 patients had free-floating thrombi. Stroke was the presenting symptom in 18 patients. |
| Khalifa | Case report | No | Saudi Arabia | 1 child | N/A | RT-PCR NPH swab | 11-year-old boy with GBS. Spinal MRI with enhancement of the cauda equina nerve roots. |
| Khoo | Case report | No | UK | 1 | N/A | RT-PCR NPH and throat swab | 65-year-old female with postinfectious brainstem encephalitis. Normal brain MRI. CSF negative for SARS-CoV-2 RNA. |
| Kilinc | Case report | No | The Netherlands | 1 | N/A | RT-PCR blood and fecal | 50-year-old male with GBS. Brain MRI normal. CSF negative for SARS-CoV-2 RNA. |
| Kim | Case report | No | South Korea | 1 | N/A | RT-PCR NPH swab | 53-year-old female with intracerebral hemorrhage on the right external capsule and putamen. |
| Kishfy | Case series | No | USA | 2 | N/A | Unclear | 2 patients with PRES. |
| Klein | Case report | No | USA | 1 | N/A | RT-PCR NPH swab | 29-year-old female with new onset seizures. Brain MRI showed hyperintense diffusion signal of the left temporoparietal lobe, hemorrhagic infarct with mass effect. |
| Klok | Retrospective cohort study | Yes | The Netherlands | 5 patients with stroke (out of 184 ICU patients) | N/A | Unclear | 5 out of 184 ICU patients (2.7%) suffered ischemic stroke. Patient data has been previously published. |
| Kong | Case report | No | China | 1 | N/A | RT-PCR unclear site | A 53-year-old female whose only symptom was sudden dizziness. Abnormal lesions on chest CT suggestive of COVID-19. |
| Kremer | Retrospective observational study | No | France | 37 | N/A | RT-PCR NPH swab | Abnormal brain MRI from 37 patients showed three patterns, which could either occur in isolation or in combination: Pattern 1 featured medial temporal lobe signal abnormalities suggestive of viral/autoimmune encephalitis; patterns 2 and 3 featured microbleeds, either in the context of multifocal white matter hyperintense lesions or as separate features, respectively. |
| Kulick-Soper | Case report | No | USA | 1 | N/A | RT-PCR NPH swab | 52-year-old female with hypoxia or acute necrotizing encephalopathy. T2 hyperintensity in the globi pallidi and multiple infarctions on brain MRI. |
| Kwee | Retrospective case series | No | The Netherlands | 27 | N/A | RT-PCR NPH swab | 11 patients with ischemic stroke, 5 patients with brain contusion, 2 patients with non-traumatic intracranial hemorrhage. The sensitivity of additional chest CT imaging in patients undergoing neuroimaging was high (86%). |
| Lang | Retrospective case series | No | USA | 6 | N/A | RT-PCR NPH swab | Out of 42 tested patients, 6 critically ill patients showed signs of leukoencephalopathy. On brain MRI, all patients exhibited symmetric T2 FLAIR hyperintense lesions and restricted diffusion in the deep white matter of both cerebral hemispheres, relatively sparing the subcortical U-fibers. Additional sites were also the corpus callosum in patient, middle cerebellar peduncles and corticospinal tracts. |
| Lantos | Case report | No | USA | 1 | N/A | RT-PCR NPH swab | 36-year-old male with left eye oculomotor nerve palsy. MRI with enlargement and enhancement of left oculomotor nerve. |
| Lascano | Case series | No | Switzerland | 3 | N/A | RT-PCR NPH swab and/or IgG/IgM | 63 and 61-year-old females with tetraparesis, 52-year-old female with tetraplegia. One patient had lumbosacral nerve enhancement on MRI. |
| Laurendon | Case report | No | France | 1 | N/A | RT-PCR unclear site | 27-year-old male with anosmia and dysgeusia. Olfactory clefts showed mild edema and T2-weighted images showed enlargement of olfactory bulbs bilaterally on brain MRI. |
| Le Guennec | Case report | No | France | 1 | N/A | RT-PCR tracheal aspirate | 69-year-old male with status epilepticus. Hyperintensity of the right orbital prefrontal cortex adjacent to the olfactory bulb on brain MRI. |
| Lechien | Cohort study | Yes | Europe (Belgium, France, Italy, Spain) | 417 (mild to moderate disease) | N/A | RT-PCR NPH swab | Out of 417 patients with mild to moderate COVID-19, 86% and 88% of patients reported olfactory and gustatory dysfunctions, respectively. These symptoms occurred before other symptoms in 12% of cases. Females were significantly more affected by olfactory and gustatory dysfunctions than males. 45% of patients had headache. After two weeks, 26% of patients recovered both olfactory and gustatory functions. |
| Lechien | Observational cohort study | Yes | Belgium, France, Italy, Spain and Switzerland | 1420 | N/A | RT-PCR unclear site | The most prevalent symptoms were headache (70.3%), loss of smell (70.2%), nasal obstruction (67.8%), cough (63.2%), asthenia (63.3%), myalgia (62.5%), rhinorrhea (60.1%), gustatory dysfunction (54.2%) and sore throat (52.9%). Fever was reported by 45.4%. Some patients added the following: conjunctivitis (N=9), visual acuity reduction (N=6), rotatory vertigo (N=6), tinnitus (N=5), cutaneous rash (N=4), cervical lymphadenopathies (N=2), and parotitis (N=1). The mean duration of Covid-19 symptoms of mild-to-moderate cured patients (N=264) was 11.5 ± 5.7 days. |
| Lechien | Retrospective survey-based study | No | Belgium | 68 | N/A | RT-PCR NPH swab | Most common symptoms were fatigue (73%), headache (60%), nasal obstruction (59%), and postnasal drip (49%). Total loss of smell was reported by 61% of patients. |
| Lechien | Retrospective cohort study | No | Belgium | 78 | N/A | RT-PCR NPH swab | 78 patients with initial sudden olfactory anosmia. |
| Lechien | Case series | No | Belgium | 16 | N/A | RT-PCR NPH swab | 16 patients with anosmia. In the brain CT scan, the olfactory clefts were opacified in 3 patients. |
| Lee | Prospective study | Yes | South Korea | 3191 | N/A | Unclear | Acute anosmia or ageusia was observed in 15.3% (488/3,191) patients in the early stage of COVID-19 and in 15.7% (367/2,342) patients with asymptomatic-to-mild disease severity. Most patients with anosmia or ageusia recovered within 3 weeks. The median time to recovery was 7 days for both symptoms. |
| Lee | Case report | No | South Korea | 1 | N/A | Unclear | 53-year-old female with delayed hyposmia, one month after recovery. |
| Levinson | Case series | No | Israel | 42 | N/A | RT-PCR NPH swab | 15 patients (36%) reported smell disorder and 14 (33%) reported taste disorder. 14 patients reported both smell and taste disorder, and 1 reported only smell disorder. |
| Li | Prospective cohort study | Yes | China | 145 | 170 | Unclear | Dysosmia may last up to 14 weeks longer in patients with COVID-19 compared to controls. |
| Li | Retrospective observational study | No | USA | 219 | N/A | RT-PCR throat swab | 10/219 (5%) had acute ischemic stroke and 1 (0.5%) had an intracerebral hemorrhage. Patients with new onset of cerebrovascular disease were older, more likely to present with severe COVID-19 and were more likely to have cardiovascular risk factors. |
| Li | Case report | No | Taiwan | 1 | N/A | RT-PCR NPH swab | 21-year-old male with anosmia. Brain MRI using T2-weighted sequences with fat suppression showed hyperintensity in olfactory bulb and reduced size right side. |
| Li | Case report | No | China | 1 | N/A | RT-PCR oropharyngeal swab | 68-year-old male with cerebral hemorrhage right temporal lobe and subarachnoid hemorrhage. |
| Li | Case report | No | China | 1 | N/A | RT-PCR unclear site | 57-year-old male in critical condition, exploratory scanning. No increased FDG uptake or abnormalities in the brain on MRI. |
| Liang | Retrospective case series | No | USA | 7 | N/A | RT-PCR NPH swab | 7 patients with malignant cerebral edema due to large hemispheric stroke. |
| Liguori | Prospective observational study | No | Italy | 103 | N/A | Unclear | 94/103 patients (93%) reported at least one neurological symptom. Sleep impairment was the most commonly reported symptom, followed by taste disorder, headache, hyposmia and depression. |
| Lodigiani | Retrospective cohort study | No | Italy | 388 | N/A | Unclear | 10/388 patients suffered ischemic stroke (2.5%). |
| Logmin | Case report | No | Germany | 1 | N/A | RT-PCR oropharyngeal swab | 70-year-old female with non-epileptic seizures. No acute brain MRI findings. |
| Lu | Retrospective study | Yes | China | 304 | N/A | RT-PCR NPH swab | Study aimed at identifying incidence of seizures in patients with COVID-19. None of included patients had a known history of epilepsy. 84/311 patients (27%) had brain insults or metabolic imbalances during the disease course known to increase the risk of seizures (hypoxia, hypocalcemia, hypokalemia, hyponatremia) but only two patients (0.7%) had seizure-like symptoms during hospitalization due to acute stress reaction and hypocalcemia. No cases of acute symptomatic seizures nor status epilepticus were observed. |
| Lyons | Case report | No | Ireland | 1 | N/A | RT-PCR NPH swab | 20-year-old male with myalgia and tonic-clonic seizures, CSF analysis demonstrated a lymphocytic pleocystosis. No acute brain MRI findings. |
| Madia | Case series | No | Italy | 6 | N/A | Unclear | 5 males, 1 female (51 to 72 years old) with flaccid quadriplegia, deemed either GBS, critical illness neuropathy or toxic myopathy. |
| Mahammedi | Retrospective observational study | Yes | Italy | 108 | N/A | RT-PCR respiratory tract swab | 108 patients with acute neurological symptoms and neuroimaging (out of 725 consecutive patients, 15%). 51 of 108 with acute pathologies on neuroimaging (47%): 34 (31%) with acute ischemic strokes, 6 (6%) with acute cerebral hemorrhage, 2 with cerebral venous thrombosis, 2 with MS plaque exacerbation, 2 with nonspecific encephalopathy, 2 with GBS, 1 with Miller-Fisher syndrome, 1 with PRES, 1 with acute encephalopathy. |
| Mahboob | Case report | No | USA | 1 | N/A | RT-PCR unclear site | 58-year-old female with cerebellar stroke (PICA territory). |
| Malentacchi | Case report | No | Italy | 1 | N/A | RT-PCR NPH site | 81-year-old male, mental confusion. Bilateral medial cerebral artery infarctions and venous thrombosis. |
| Manganelli | Case series | No | Italy | 3 | N/A | Unclear | 3 COVID-19 patients in the ICU who had had recovered from pneumonia but could not be weaned from invasive mechanical ventilation. Clinical evaluation showed involvement of the brainstem and especially of the respiratory center. |
| Manganotti | Case series | No | Italy | 5 | N/A | RT-PCR NPH swab | 4 patients with flaccid paresis, 1 with bilateral ophthalmoplegia, trigeminal hypoesthesia and facial palsy. 49-92 years old. 2 with normal brain MRI findings. |
| Manganotti | Case report | No | Italy | 1 | N/A | Unclear, bilateral pneumonia | 50-year-old female with diplopia and facial paresthesia as well as limb areflexia and ataxia. MRI normal. |
| Mao | Retrospective, observational case series | Yes | China | 214 | N/A | RT-PCR NPH swab and/or high-throughput sequencing | 78/214 patients (36%) had neurologic manifestations, among them dizziness (17%), headache (13%), impaired consciousness (8%), acute cerebrovascular disease (3%), ataxia (0.5%), seizure (0.5%), peripheral nervous system manifestations (9%), ageusia (6%), anosmia (5%), vision (1%), skeletal muscle injury (11%). Most symptoms started within the first 4 weeks upon admission. Patients with more severe infection had more neurologic complications compared to patients with less severe disease course: cerebrovascular insults (5 [6%] versus 1 [1%]), impaired consciousness (13 [15%] versus 3 [2%]), and skeletal muscle injury (17 [19%] versus 6 [5%]). Patients with CNS symptoms had lower lymphocyte levels, platelet counts and higher blood urea nitrogen levels compared with those without CNS symptoms. |
| Marta-Enguita | Case report | No | Spain | 1 | N/A | RT-PCR unclear site | A 76-year-old female with tetraparesis with distal-onset paresthesia. 8 days before onset she had fever and cough. Suspect GBS. |
| Matos | Case report | No | Portugal | 1 | N/A | RT-PCR NPH | 42-year-old male with apathy, altered mental status. Multiple lesions on T2-weighted FLAIR bilaterally in the white matter, as well as thalami, basal ganglia and pons. Diffusion restriction in some brain regions. |
| Maurier | Case report | No | France | 1 | N/A | PCR unclear site | 58-year-old female with no expansion of the lungs during inspiration and restrictive pulmonary syndrome. |
| Mawhinney | Case report | No | UK | 1 | N/A | RT-PCR NPH | 41-year-old male with headache, mania and grandiose thoughts. Normal behavior on 23-day follow up. Brain MRI normal. |
| McAbee | Case report | No | USA | 1 child | N/A | RT-PCR NPH | 11-year-old male with new onset of status epilepticus and CSF indicating encephalitis. |
| Mehrpour | Case series | No | Iran | 10 | 21 | RT-PCR NPH swab | 10 stroke patients positive for COVID-19 had a higher mean of age, a difference in the type of stroke and higher stroke severity. |
| Melley | Case report | No | USA | 1 | N/A | RT-PCR NPH swab | 59-year-old female with hypogeusia. |
| Méndez-Guerrero | Case report | No | Spain | 1 | N/A | RT-PCR NPH swab | 58-year-old male with myoclonus at rest 23 days after mechanical ventilation, transient period of impaired consciousness. Sudden spontaneous improvement. No acute brain MRI findings. |
| Mermelstein | Case report | No | Brazil | 1 | N/A | RT-PCR unclear site | Self-report by a 27-year-old female. Anosmia for 15 days, onset 6 days after myalgia and cough. |
| Merkler | Retrospective cohort study | Yes | USA | 31 | 3 | RT-PCR NPH swab | 31/1916 (1.6%) with emergency department visits/hospitalizations with COVID-19 had acute ischemic stroke. Only 3/1486 with influenza (0.2%) had an acute ischemic stroke. |
| Mirzaee | Case report | No | Iran | 1 child | N/A | PCR NPH | 12-year-old child with onset of seizures and hemiparesis as well as dysarthria. Brain MRI showed acute infarction left basal ganglia and narrowed left proximal M1-segment on Time-of-Flight MR angiography. |
| Mohamud | Case series | No | USA | 6 | N/A | RT-PCR NPH swab | 6 patients with acute ischemic stroke due to intraluminal carotid artery thrombus. |
| Morassi | Retrospective case series | No | Italy | 6 | N/A | RT-PCR NPH swab | 6 patients with acute stroke. 5 of them with fatal outcome. |
| Morassi | Case report | No | Italy | 1 | N/A | RT-PCR NPH swab | 58-year-old male with acute bilateral carotid dissections. |
| Moriguchi | Case report | No | Japan | 1 | N/A | RT-PCR NPH swab and CSF | 24-year-old male with progressive headache and fatigue. Day 9 after symptom onset GCS deterioration (6/15) and convulsions and neck stiffness. SARS-CoV-2 RNA was not detected in the NPH swab but was detected in CSF. Brain MRI showed hyperintensity along the wall of the right lateral ventricle and hyperintense signal changes in the right mesial temporal lobe and hippocampus, compatible with meningitis. |
| Moshayedi | Case report | No | USA | 1 | N/A | RT-PCR respiratory tract | Male in his 70s with acute myocardial infarction as well as acute infarction in the cerebellum, major parts of the left cerebral hemisphere and right caudate nucleus. |
| Muhammad | Case report | No | Germany | 1 | N/A | RT-PCR oropharyngeal swab | 60-year-old female, left frontal brain hemorrhage, ruptured pericallosal aneurysm. |
| Munz | Case report | No | Germany | 1 | N/A | RT-PCR oropharyngeal swab | 60-year-old male, bladder dysfunction and hypoesthesia below Th9-level as well as spastic paraparesis. Lesion on spinal MRI at the Th3-5 and Th9 levels suggestive of transverse myelitis. |
| Nepal | Case report | No | USA | 1 | N/A | RT-PCR oropharyngeal swab | 50-year-old male found unresponsive. Brain MRI showed multiple punctate lesions with restricted diffusion, suggesting multiple acute infarctions or vasculitis. |
| Nicholson | Case series | No | Canada | 4 | N/A | Unclear | 4 patients (43-62 years old), 3 with extracorporeal membrane oxygenation treatment and 1 on ventilator. All with altered mental status when extubated, except the 42-year-old who developed a larger intracranial hemorrhage with acute pupil asymmetry. Susceptibility-weighted images showed multiple suspected microhemorrhages in multiple lobes in 3 of the patients. Diffusion restriction and T2-weighted FLAIR showed multiple cortical infarctions. |
| Noro | Case report | No | Brazil | 1 | N/A | RT-PCR NPH swab | 35-year-old female with headache and increased intracranial pressure (40cm H_2_O). Brain MRI showing indirect signs of increased intracranial pressure. |
| Novi | Case report | No | Italy | 1 | N/A | RT-PCR CSF | 64-year-old female with sudden bilateral vision impairment and sensory deficit in the right leg. Hyperreflexia right leg. MRI with multiple enhancing lesion in brain and spinal column as well as optic nerves. ADEM. |
| Oguz-Akarsu | Case report | No | Turkey | 1 | N/A | RT-PCR NPH swab | 53-year-old female with dysarthria and weakness in lower limbs. MRI of spine showing asymmetrical thickening and hyperintensity in postganglionic roots in brachial and lumbar plexus. GBS. |
| Oliveira | Case report | No | Brazil | 1 | N/A | RT-PCR NPH swab | 69-year-old male with diplopia and headache. Bilateral trochlear paresis. MRI angiography suggested vasculitis. Enhancement of basilar artery wall as well as vertebral arteries. |
| Ollarves-Carrero | Case report | No | Spain | 1 | N/A | PCR unclear site | 40-year-old female with anosmia. |
| Ottaviani | Case report | No | Italy | 1 | N/A | RT-PCR NPH swab | 66-year-old female who 7 days after developing cough and fatigue gets progressively weaker in the lower limbs. Suspect GBS. |
| Ottaviano | Case series | No | Italy | 6 | N/A | RT-PCR NPH swab | 6 SARS-2-CoV patients with exclusive sudden anosmia. Le Nez du Vin confirmed the olfactory loss in all of them. Symptoms still present after 15 days. |
| Oxley | Case series | No | USA | 5 | N/A | RT-PCR unclear site | 5 patients < 50 years old with ischemic stroke. |
| Padroni | Case report | No | Italy | 1 | N/A | RT-PCR NPH swab | A 70-year-old female with bilateral paresthesia. GBS. |
| Palomar-Ciria | Case report | No | Spain | 1 | N/A | IgM and IgG-antibodies in serum. | 65-year-old male with over 20 years of stable schizophrenia, severe worsening. No symptoms of respiratory disease. Serum antibodies to SARS-CoV-2, suspected cause of worsening. |
| Panariello | Case report | No | Italy | 1 | N/A | Chest CT | 23-year-old male, substance use disorder. Sudden agitation, disorganization and hallucinations. THC in urine sample. Anti-NMDA-receptor antibodies in CSF. |
| Paniz-Mondolfi | Case report | No | USA | 1 | N/A | RT-PCR NPH swab, electron microscopy | 74-year-old male with Parkinson’s disease. Brain CT with signs of known vascular leukoencephalopathy. Progressive delirium. Transmission electron microscopy of post-mortem frontal lobe brain sections showed the presence of 80 to 110 nm viral particles. Neuronal cell bodies with cytoplasmic vacuoles containing enveloped viral particles exhibiting electron dense centers suggestive for coronavirus. |
| Papi | Case report | No | Italy | 1 | N/A | Post-mortem PCR NPH. | 79-year-old female with wake-up stroke. Left middle artery occlusion. Thrombectomy, died 4 days later. |
| Parsons | Case report | No | USA | 1 | N/A | RT-PCR NPH swab | 51-year-old female. 18 days after intubation flaccid muscle tone. No spontaneous movement of limbs. Lowered GCS (3) without sedatives. Brain MRI with multiple lesions with restricted diffusion. Frontal lesions with cortical enhancement. ADEM. Full recovery 41 days later. |
| Paybast | Case series | No | Iran | 2 | N/A | RT-PCR oropharyngeal swab | 38-year-old male with ascending paresthesia and bilateral facial droop. GBS. 14-year-old daughter developed GBS at the same time. |
| Paterson | Case series | No | UK | 43 patients (29 with definite COVID-19 diagnosis, some of them published as case reports) | N/A | RT-PCR NPH swab | 43 COVID-19 patients which were referred to the COVID-19 multidisciplinary team neurology/encephalitis and neurovascular unit. Patients could be classified according to five major neurological disease classes; 1) encephalopathy: 10 patients, clinical presentation mostly with confusion/disorientation, 1 with psychosis, 1 with seizure; 2) inflammatory CNS syndromes (para-/postinfectious): 12 patients, 2 with encephalitis (brain stem/limbic encephalitis), 9 with ADEM, 4 patients with hemorrhagic change on imaging, including microbleeds; 3) stroke: 8 patients with ischemic stroke in the context of hypercoagulability; 4) PNS syndromes: 7 patients with GBS. (5) Miscellaneous: Abducens nerve palsy, pediatric patient with status epilepticus. |
| Peng | Cross-sectional study | No | China | 11 | N/A | RT-PCR NPH swab | Out of 86 patients with upper respiratory tract infection, 11 (13%) were tested positive for SARS-CoV-2. 7/11 patients with fatigue (64%). No difference in prevalence of fatigue between COVID-19 and non-COVID-19 patients. |
| Pérez Álvarez | Case report | No | Spain | 1 | N/A | RT-PCR sputum | 48-year-old male with known paranoid schizophrenia. Sudden binocular diplopia, earlier upper respiratory symptoms. Spontaneous recovery. Positive in serum for anti-acetylcholine receptor antibodies. |
| Pfefferkorn | Case report | No | Germany | 1 | N/A | PRT-CR oropharyngeal swab | 51-year-old male with progressive weakness of upper and lower limbs. Complete loss of sensory functions. 2 weeks of flu-like symptoms. Spinal MRI showing massive symmetrical enhancement of spinal nerve roots and cauda equina. Suspected para-infectious GBS. |
| Pilotto | Case report | No | Italy | 1 | N/A | RT-PCR NPH swab | 60-year-old male with altered conciousness. 5 days of worsening irritability and confusion. CSF showing mild lymphocytic pleocytosis. 5 days of high-dose steroids leading to recovery. |
| Pinna | Retrospective observational case series | No | USA | 50 | N/A | RT-PCR NPH swab | 50 patients with the most common neurological complications being encephalopathy (30 patients), cerebrovascular disease (20 patients), cognitive impairment (13 patients), seizures (13 patients), hypoxic brain injury (7 patients), taste disorder (5 patients) and extraocular movement abnormalities (5 patients) |
| Pinto | Case report | No | USA | 1 | N/A | RT-PCR NPH swab | 44-year-old female with gradual hand incoordination 7 days before onset of respiratory symptoms. Progressive worsening with right arm weakness and suspected stroke. MRI showed periventricular lesions with enhancement. Developed aphasia. Anti-MOG disease with antibodies. |
| Poillon | Case series | No | France | 2 | N/A | RT-PCR NPH and chest CT. | 62-year-old female with intraparenchymal hemorrhage in the left frontotemporal lobes and thrombosis of the left transverse sinus, straight vein, vein of Galen and internal cerebral veins. 54-year-old female with hemorrhagic infarction in the left temporal lobe and thrombosis of left transverse sinus. |
| Politi | Case report | No | Italy | 1 | N/A | RT-PCR unclear site | 25-year-old female with anosmia and dysgeusia. Brain MRI with cortical hyperintensity and hyperintesity on T2-weighted FLAIR images. Normalization of MRI finding 28 days later. |
| Pons-Escoda | Cross-sectional study | No | Spain | 103 | N/A | RT-PCR unclear site | 103/2249 underwent neuroimaging (5%). Most frequent symptoms motivating neuroimaging were mild non-focal neurologic symptoms, stroke, focal neurologic symptoms, post-sedation encephalopathy, and seizures. 13 patients with acute ischemic events, and 7 patients with hemorrhagic events. Most patients had at least one cardiovascular risk factor. |
| Poyiadji | Case report | No | USA | 1 | N/A | RT-PCR NPH swab | A female patient with acute necrotizing/hemorrhagic encephalopathy. Brain MRI demonstrated hemorrhagic rim enhancing lesions within the thalami, medial temporal lobes as well as subinsular regions. |
| Princiotta Cariddi | Case report | No | Italy | 1 | N/A | PCR NPH | 64-year-old female, 10 days of fever and dyspnea. 25 days of sedated ventilation, then altered mental status and blurred vision. Weakness left side of face and bilateral lower limbs with reduced reflexes. Brain CT and MRI with signs of PRES. Day 56 with reduction of brain edema on MRI. |
| Qin | Retrospective cohort study | No | China | 452 (166 non-severe, 286 severe disease courses) | N/A | RT-PCR NPH swab | Study compared symptomatology among patients with severe and non-severe COVID-19 courses. Neurological symptoms were fatigue (212/463, 46% overall; 39% in non-severe, 51% in severe patients), headache (52/452, 11% overall; 8% in non-severe patients, 14% in severe patients) and confusion (3/452, 0.7% overall; 0% in non-severe patients, 1% in severe patients). |
| Rábano-Suárez | Case series | No | Spain | 3 | N/A | 1 of them with RT-PCR NPH swab, the rest on symptomatology. | 2 male and 1 female between 63-88 years of age with onset of mild hypersomnia and generalized myoclonus after COVID-19. Brain MRI was normal in all cases. |
| Radmanesh | Case report | No | USA | 1 | N/A | RT-PCR NPH swab | 59-year-old male with one week of dyspnea. Altered mental status 2 weeks after. EEG with moderate diffuse slowing. Brain CT with bilateral hypodensities in the supratentorial white matter. Brain MRI with confluent T2 hyperintensities in the white matter sparing U-fibers and areas of suspected necrosis. Probable diagnosis of delayed post-hypoxic leukoencephalopathy. |
| Radmanesh | Case series | No | USA | 11 | N/A | RT-PCR NPH swab | Neuroimaging in 11 critically-ill patients. Main imaging features: 1) confluent T2 hyperintensity and mild restricted diffusion in supratentorial deep and subcortical white matter. 2) multiple microbleeds, located mainly in juxtacortical and callosal white matter. |
| Radmanesh | Retrospective cohort study | No | USA | 242 with neuro-imaging | N/A | RT-PCR NPH swab | 3 most common indications for neuroimaging were altered mental status (42%), syncope/fall (33%), and focal neurologic deficit (12%). Most common imaging findings were nonspecific white matter microangiopathy (134 patients, 55%), chronic infarct (47 patients, 19%), acute or subacute ischemic infarct (13 patients, 5%) and acute hemorrhage (11 patients, 5%). Patients with white matter microangiopathy showed higher 2-week mortality. |
| Rana | Case report | No | USA | 1 | N/A | RT-PCR unclear location | 54-year-old male with GBS. No CSF sampling or brain MRI obtained. |
| Reddy | Case series | No | USA | 12 | N/A | Unclear | 10 patients with acute ischemic stroke, 2 patients with intracerebral hemorrhage. Etiologies ranging from cardioembolic to carotid dissection to infectious endocarditis. One hemorrhagic stroke  likely hypertensive and another related to mycotic aneurysm from infectious endocarditis. |
| Regev | Case report | No | Israel | 1 | N/A | RT-PCR NPH swab | 16-year-old male with Kawasaki syndrome with brain MRI signs of CNS small vessel vasculitis. |
| Reichard | Case report | No | USA | 1 | N/A | Unclear | Hospitalized patient due to coronary artery disease developed postoperatively COVID-19. Neuropathological assessment showed hemorrhagic white matter lesions throughout the cerebral hemispheres with adjacent axonal injury. Additionally, subcortical white matter had clusters of macrophages and associated axonal injury, and ADEM-like appearance. Few neocortical organizing microscopic infarcts were also observed. |
| Reyes Bueno | Case report | No | Spain | 1 | N/A | RT-PCR unclear site | 51-year-old female diagnosed with Miller-Fisher syndrome two weeks after COVID-19 . |
| Rigamonti | Case report | No | Italy | 1 | N/A | Unclear | 54-year-old male with headache and aphasia, 2 weeks of fever and cough. Right hemianopsia and hemiparesis. Brain CT showed hemorrhage and hypodensity in left basal ganglia and thalamus. Deep vein thrombosis left side on CT angiography. |
| Riva | Case report | No | Italy | 1 | N/A | IgG positive | Male in his 60s, sensory deficit in lower limbs 20 days after fever and cough. Weakness spread to upper limbs and cranial nerves over 10 days, then recovery. RT-PCR CSF negative as well as RT-PCR NPH swab. GBS. |
| Rogg | Case report | No | USA | 1 | N/A | Unclear, says tested positive | 59-year-old male with PRES 5 days after intubation. MRI with T2-weighted FLAIR images with extensive whiter matter hyperintensity, no enhancement. Died 2 days later. |
| Romero-Sanchez | Observational retrospective cohort study | Yes | Spain | 483 | N/A | RT-PCR NPH or blood IgG/IgM | 483/841 (57%) of hospitalized patients had neurological symptoms, among them disorders of consciousness (20%), headache (14%) and dizziness (6%). Smell and taste disorders were common initial symptoms. 4% of patients died due to neurological complications (most commonly stroke [8 patients] and intracranial hemorrhage [3 patients]). |
| Roy-Gash | Case report | No | France | 1 | N/A | IgG/IgM positive ELISA | 63-year-old female with aphasia and right hemiplegia. Brain MRI with large left temporal hemorrhage. CT angiography showed venous thrombosis. New contralateral bleeding 14 days later. EEG with atypical background and paroxysmal patterns. |
| Rudilosso | Case report | No | Spain | 1 | N/A | Unclear | 50-year-old male with bilateral pneumonia, non-invasive ventilation. Two days later, despite anticoagulation, right facial palsy and weakness of limbs. Brain MRI showed acute infarction left medial thalamus. |
| Sachs | Case report | No | USA | 1 | N/A | RT-PCR unclear site | 59-year-old male with upper respiratory symptoms, severe agitations on hospital day 6. Brain MRI showed bilateral microhemorrhage in the corpus callosum and posterior white matter abnormalities. ADEM. |
| Saggese | Case report | No | Italy | 1 | N/A | Negative RT-PCR NPH | 62-year-old male with acute right hemiplegia, neglect and aphasia. Brain CT and CT angiography normal. Ground glass patterns on chest CT. Thrombophlebitis of left forearm and occlusion of right dorsalis pedis artery. New brain CT with hemorrhagic transformation of left frontotemporal ischemic lesion. |
| Sancho-Saldana | Case report | No | Spain | 1 | N/A | RT-PCR NPH swab | 56-year-old female with GBS. |
| Sangalli | Case series | No | Italy | 4 | N/A | Mixed proof, 1 with RT-PCR NPH | 4 patients (53-77 years old) presenting with ischemic stroke. Cough and fever prior to infarction. All had thrombolysis, 1 survived. The rest had hemorrhagic transformation. |
| Scheidl | Case report | No | Germany | 1 | N/A | RT-PCR oropharyngeal | A 54-year-old female presenting acute demyelinating polyradiculoneuropathy, a common form of GBS. |
| Schupper | Case series | No | USA | 2 children | N/A | RT-PCR NPH swab | 5-year-old boy with right middle cerebral artery stroke, cerebral edema, and diffuse contralateral subarachnoid hemorrhage. 2-month-old boy with seizures and hemorrhagic strokes in bilateral parieto-occipital lobes, left temporal and left frontal lobes, likely of cardioembolic origin. |
| Scullen | Retrospective cross-sectional study | No | USA | 27 | N/A | RT-PCR unclear site | 27 out of 76 critically ill COVID-19 patients (36%) with neurological sequelae. Heterogenous presentation with encephalopathy, acute necrotizing encephalopathy and/or vasculopathy. |
| Sedhagat | Case report | No | Iran | 1 | N/A | RT-PCR NPH swab | A 65-year-old male with diabetes mellitus type 2 suffered acute progressive symmetric ascending quadriparesis after two weeks of respiratory symptoms. No CSF analysis. Brain MRI normal. Clinical picture suggestive of GBS. |
| Selvaraj | Case report | No | USA | 1 | N/A | Positive, unclear test | Female in her 50s, acute right eye monocular visual disturbances. Right side subjective hemiparesis. Improvement one day later. Brain MRI normal. Differential diagnosis included central retinal artery occlusion, central retinal vein occlusion, retinal detachment and optic neuropathy. |
| Sharifi-Razavi | Case series | No | Iran | 3 | N/A | RT-PCR throat swab | 4 patients (55-88 years old) with ischemic stroke. Prior cough and dyspnea. 2 with brain CT and acute infarction, 1 with normal brain CT but clinical stroke due to dysarthria. |
| Sharifi-Razavi | Case report | No | Iran | 1 | N/A | RT-PCR NPH swab | 79-year-old male with acute loss of consciousness and GCS deterioration (GCS 7/15) after 3 days of coughing. Brain CT revealed a massive intracerebral/intraventricular and subarachnoid hemorrhage in the right hemisphere. No hypertension or anticoagulation. |
| Shoskes | Case report | No | USA | 1 | N/A | RT-PCR NPH swab | 69-year-old male, one week with cough, fever and dyspnea. Developed purpura with biopsy showing thrombotic vasculopathy. Altered mental status, brain MRI showed multiple susceptibility weighted imaging changes throughout the bilateral juxtacortical white matter, corpus callosum, basal ganglia, and brainstem. Died shortly after due to respiratory failure. |
| Sierpinski | Cross-sectional survey-based study | N/A | Poland | 1942 | N/A | RT-PCR nasal/throat swab | 54% of patients reported at least 1 olfactory or taste disorder and 43% reported both alterations. |
| Sierra-Hidalgo | Case series | No | Spain | 8 | N/A | RT-PCR unclear site | Median age 69, 7 out of 8 were male. Ischemic stroke with median onset 11.5 days after onset of COVID-19 symptoms. 5 had stroke in one cerebral artery territory and 3 in two or more territories. None with atherosclerosis. |
| Singh | Case report | No | USA | 2 | N/A | RT-PCR NPH swab | Two women with severe persistent headache as initial symptom of COVID-19, both had a prior history of migraine. |
| Singh | Case report | No | USA | 1 | N/A | RT-PCR unclear site | 36-year-old female with stable Myasthenia Gravis. Exacerbation of symptoms including ptosis, dysphagia and weakness at the same time as fever and cough. Intubated. Recovery of COVID-19 also meant reduction of Myasthenia Gravis symptoms. |
| Sohal | Case report | No | USA | 1 | N/A | RT-PCR unclear site | A 72-year-old man with hypertension and end-stage kidney disease. Felt dizzy after hemodialysis and during his hospital stay had episodes of tonic-clonic movements. EEG showed temporal seizures. He died shortly after. |
| Soldatelli | Case report | No | Brazil | 1 | N/A | Unclear | Perfusion abnormalities (using arterial spin labeling) in an acutely ill patient. |
| Solomon | Case series | No | USA | 18 | N/A | RT-PCR NPH swab | Neuropathological assessment was suggestive of diffuse hypoxic brain injury. No signs of encephalitis or other specific brain  changes referable to the virus. Immunohistochemistry and PCR presented only minimal evidence of SARS-CoV-2 central nervous system infection. |
| Somani | Case series | No | USA | 2 | N/A | RT-PCR NPH swab and tracheal aspirate | A 49-year-old female presenting with multiple seizures and pathological EEG as well as a 73-year-old female with myoclonus and EEG suggested discrete seizures. |
| Sotoca | Case report | No | Spain | 1 | N/A | RT-PCR NPH swab | 69-year-old female with 7 days of cervical pain, imbalance and numbness of left hand. Fever and cough 15 days prior to hospital visit. Right facial and left-hand hypoesthesia, subtle left-hand interosseous weakness, and general hyperreflexia. Brain MRI normal, spinal MRI with diffuse patchy enhancing lesions indicating acute transverse myelitis. Later spinal MRI showed necrosis, diagnosis of acute necrotizing myelitis. Negative SARS-CoV-2 PCR in CSF. |
| Speth | Prospective cross-sectional study | No | USA | 103 | N/A | RT-PCR unclear site | Olfactory dysfunction in 61 % of COVID-19 patients. Nasal obstruction not correlated to olfactory dysfunction. |
| Speth | Prospective cross-sectional study | No | USA | 114 | N/A | RT-PCR unclear site | Severity of smell and taste loss were associated with depressed mood and anxiety. Emotional disturbances may thus be another symptom of COVID-19. |
| Spoldi | Retrospective cross-sectional study | No | Italy | 63 | N/A | Unclear | 16/63 patients with olfactory cleft mucosal thickening on brain CT. |
| Su | Case report | No | USA | 1 | N/A | RT-PCR NPH swab | 72-year-old male with mild diarrhea, no fever or cough. 6 days later weakness of limbs. Reduced sensation in limbs. Brain CT normal. CSF PCR negative. Spontaneous improvement 12 days later. GBS. |
| Sun | Case series | No | China | 2 | N/A | RT-PCR NPH swab | 8 children (0.5-15 years old) with severe COVID-19. Some of them had neurological symptoms including headache (1/8, 12.5%) and fatigue (1/8, 12.5%). |
| Sweid | Retrospective case series | Yes | USA | 22 | N/A | Unclear | 17 patients with acute ischemic stroke, 3 patients with aneurysm rupture, 2 patients with sinus thrombosis. |
| Tapé | Case report | No | USA | 1 | N/A | RT-PCR unclear site | 79-year-old female presenting with syncope. Had fever and cough for 3 days. No brain CT or MRI. |
| Tatu | Case report | Yes | France, Switzerland | 7 | N/A | No proof | 1 patient with EBV. Negative PCR for SARS-CoV-2 in all 6 other patients. GBS. No other etiology discovered. 1 died due to a severe acute respiratory syndrome. A higher incidence of GBS than normal in the same region, typically 0-2 cases during the same time interval according to the last 3 years. |
| Torabi | Case series | No | Iran | 3 | 3 | RT-PCR unclear site | Average TNF-α levels were higher in the olfactory epithelium of the COVID-19 patients compared to the control group. |
| Toscano | Case series | Yes | Italy | 5 | N/A | 4 with RT-PCR NPH swab, 1 with serology | 4 with lower limb paresis, 1 with facial diplegia and ataxia. They developed flaccid paresis over 1.5- 4 days. GBS. All negative PCR CSF. All had fever or cough 5-10 days prior to paresis. |
| Tunç | Case series | No | Turkey | 4 | N/A | RT-PCR NPH and oropharyngeal swab | 4 patients between 45 and 77 years old with cerebral infarction. Mild to moderate symptoms of COVID-19 before attending hospital for their neurological symptoms. |
| Turbin | Case series | No | USA | 2 children | N/A | RT-PCR NPH swab | Case 1: 12-year-old male with unilateral orbital swelling right side. No fever or respiratory symptoms. Brain MRI with frontal dural enhancement. Case 2: 15-year-old male with unilateral orbital swelling, mild rhinorrhea and headache. Brain CT showed thrombophlebitis of the right superior ophthalmic vein. MRI with marked enhancement of the periorbita. Thrombosis in cavernous sinus and dural venous sinuses. Pachymeningeal enhancement of frontal lobes and epidural abscess posterior of right frontal sinus. |
| Tsivgoulis | Prospective cohort study | No | Greece | 22 | 22 | RT-PCR NPH swab | Taste disorders commonly reported by patients (23%). |
| Utukuri | Case report | No | USA | 1 | N/A | RT-PCR NPH swab | 44-year-old male presenting with bilateral lower limb weakness and urinary retention. Examination showed lethargy, dysarthria, bilateral arm ataxia, urinary retention, and weakness of both legs. Brain CT normal. MRI spine with expansion of conus medullaris with mild T2-weighted hyperintensity and minimal foci of enhancement. Diagnosis of transverse myelitis. MRI showed several non-enhancing lesions in the periventricular and juxtacortical white matter as well as spine. CSF PCR negative. Diagnosis suspected as ADEM. |
| Vacchiano | Prospective survey-based study | N/A | Italy | 108 | N/A | Unclear | Patients reported the following neurological symptoms: taste disorder (61%), headache (43%), smell disorder (37%) and dizziness (10%). |
| Vaira | Case series | No | Italy | 19 | N/A | Unclear | 74% of the patients reported having or having had smell or taste disorders. |
| Valderrama | Case report | No | USA | 1 | N/A | RT-PCR NPH swab | 52-year-old male with the left middle cerebral artery occlusion and infarction. |
| Varatharaj | Nation-wide surveillance study | Yes | UK | 153 | N/A | RT-PCR NPH swab | Nationwide surveillance study of acute neurological and psychiatric complications of COVID-19. 77 of 125 patients (62%) had a cerebrovascular event (74% with ischemic stroke, 12% with intracerebral hemorrhage, 1 patient with CNS vasculitis). 39 of 125 patients (31%) presented with altered mental status consisting of 9 patients (23%) with encephalopathy and 7 patients (18%) with encephalitis. The remaining 23 patients (59%) with altered mental status fulfilled the clinical case definitions for psychiatric diagnoses, most of them being newly diagnosed (92%). 10 out of 23 patients (43%) with neuropsychiatric disorders had new-onset psychosis, six patients (26%) had a neurocognitive syndrome, and four patients (17%) had an affective disorder. 18 of 37 patients (49%) with altered mental status were younger than 60 years. |
| Vargas-Gandica | Case series | Yes | Germany, Bolivia, Columbia, USA | 10 | N/A | RT-PCR unclear site | 10 patients with viral-associated olfactory and taste loss. |
| Velayos Galán | Case report | No | Spain | 1 | N/A | RT-PCR unclear site | 43-year-old male with weakness in all four limbs, 10 days prior with respiratory infection. GBS diagnosis. |
| Vellieux | Case series | No | France | 2 | N/A | Case 1: IgM and IgG, Case 2: PCR NPH | Case 1: 37-year-old male with fever and myalgia, 3 days later he developed incoherent speech. ECMO because of acute myocarditis. EEG with slightly asymmetric, monomorphic, diphasic, delta slow waves with diffuse projection but greater amplitude over both frontal areas. Brain MRI with hypoxic encephalopathy, right middle cerebral artery infarction. Case 2: 42-year-old male with dyspnea and fever, EEG with same pattern as Case 1. |
| Vespignani | Case series | No | France | 26 | N/A | RT-PCR NPH swab | 5/26 patients (19%) had EEGs showing periodic discharges consisting of high-amplitude frontal monomorphic delta waves with absence of epileptic activity. |
| Viguier | Case report | No | France | 1 | N/A | RT-PCR NPH swab | A 66-year-old male with thrombi in the left common carotid artery and ischemia in the left middle cerebral artery territory. |
| Virhammar | Case report | No | Sweden | 1 | N/A | RT-PCR NPH swab and CSF | 55-year-old female with fever and myalgia. Suddenly lethargic and found unresponsive. Brain CT with bilateral thalami hypodensities. Worsened and intubated, new brain CT with signs of acute necrotizing encephalitis. Brain MRI suggestive of acute necrotizing encephalitis. PCR CSF positive on third try. |
| Vollono | Case report | No | Italy | 1 | N/A | RT-PCR NPH and oropharyngeal swab | A 78-year-old female with myoclonic jerks in the right limbs. One day after she developed status epilepticus. Brain CT and MRI showed no acute changes. |
| Von Weyhern | Case series | No | Germany | 6 | N/A | Unclear | 3 patients < 65 years old died of cardiorespiratory failure. The 3 younger patients died either of massive intracranial hemorrhage or pulmonary embolism. Latter patients showed diffuse petechial hemorrhages in the entire brain. All 6 patients groups showed lymphocytic panencephalitis and meningitis, including in the brain stem. |
| Vu | Case series | No | USA | 3 | N/A | RT-PCR unclear site | One case with a 30-year-old male with spontaneous left basal ganglia hemorrhage without underlying vascular malformation. |
| Wang | Case report | No | China | 1 | N/A | Unclear | A case with tuberculous meningitis and COVID-19 symptoms. |
| Webb | Case report | No | UK | 1 | N/A | PCR NPH | 57-year-old male with progressive bilateral flaccid motor and sensory neuropathy. Negative CSF PCR. 1-week history of cough and myalgia. GBS. |
| Williams | Case report | No | UK | 1 | N/A | PCR NPH | 38-year-old female with dysarthria. 1-week history of fever and cough. On day 11 worsening with right hemiparesis and facial weakness as well as increased tonus in the left upper limb. Brain MRI showing bilateral acute infarctions in the internal border zone on 11 spots, suggesting CADASIL. |
| Wei | Case report | No | China | 1 | N/A | RT-PCR throat swab | 62-year-old male with onset of left oculomotor nerve dysfunction. Normal brain MRI (including angiography). |
| Wong | Case report | No | UK | 1 | N/A | RT-PCR upper respiratory tract | 40-year-old male presenting with diplopia, oscillopsia, limb ataxia, altered sensation in right arm, hiccups and dribbling when eating or drinking. Brain MRI with hyperintense signal in the right inferior cerebellar peduncle and microhemorrhage suggestive of rhombencephalitis. |
| Xiong | Retrospective cohort study | Yes | China | 917 | N/A | RT-PCR NPH swab | Frequency of new onset critical neurologic events was 4% (32/917) overall and 9% (30/319) among those with severe or critical COVID-19. These included impaired consciousness (25, 3%) or/and stroke (10, 1%). The risk of such neurologic events was associated with higher age (> 60) and previous history of neurological conditions. Non-critical events were seen in less than 1% of patients (7/917), including headache, occipital neuralgia and tremor. |
| Yaghi | Retrospective cohort study | Yes | USA | 3556 | N/A | RT-PCR NPH swab | 32 patients (0.9%) had imaging proven ischemic stroke. COVID-19 patients with stroke had more severe disease with a higher mortality compared to non-COVID-19 stroke patients. |
| Yin | Case report | No | China | 1 | N/A | RT-PCR NPH swab and CSF | 64-year-old male patient without cardiovascular risk factors with progressive muscle soreness, deterioration of consciousness and psychiatric symptoms. Neurological exam with pyramidal signs and neck stiffness. Brain CT normal. CSF negative for SARS-CoV-2 RNA. Patient fully recovered within 2 – 3 weeks. |
| Zachariadis | Case report | No | Switzerland | 1 | N/A | Later IgG and IgM. | 63-year-old male who developed paresthesia in his feet 12 days after cough and myalgia. Sensory level of Th10. Negative PCR in CSF and NPH for SARS-CoV-2. MRI showed no abnormality. He developed sphincter malfunction and complete anesthesia below Th10. Recovered 30 days later. Transverse myelitis. |
| Zahid | Case report | No | USA | 1 | N/A | Positive, unclear method. | 38-year-old male with fever and dyspnea. ECMO. Left intracranial hemorrhage on brain CT. |
| Zanin | Case report | No | Austria | 1 | N/A | RT-PCR unclear site | A 54-year-old female with an anterior communicating artery aneurysm treated surgically 20 years ago. Found unconscious. Anosmia and ageusia since multiple days. Normal brain CT. EEG showing two seizures starting from the right frontotemporal lobe. |
| Zayet | Case series | No | France | 2 | N/A | RT-PCR NPH swab | Case 1: 68-year-old male with altered consciousness. Intubated. Brain MRI normal. Spontaneous recovery. Case 2: 39-year-old male with headache, anosmia and dysgeusia. Dyspnea. Progressive drowsiness, GCS 12. Spontaneous recovery in 3 days. |
| Zayet | Case series | No | France | 2 | N/A | RT-PCR NPH swab | Case 1: 84-year-old male with fever. Developed dysarthria, left hemiplegia, and alteration of consciousness, brain MRI showed acute infarction in multiple vascular territories. Case 2: 74-year-old male with disorientation and influenza-like symptoms. Aphasia. Brain CT with infarction in multiple vascular territories. |
| Zhai | Case report | No | China | 1 | N/A | RT-PCR throat swab | 79-year-old male with CT-confirmed stroke. |
| Zhang | Case report | No | USA | 1 | N/A | RT-PCR unclear site | 58-year-old female with cough, dyspnea and myalgia. Symmetric proximal limb weakness. MRI of brain and spinal cord showed no abnormalities in the nerves, though diffuse muscle edema and enhancement as well as myonecrosis. |
| Zhao | Case report | No | China | 1 | N/A | RT-PCR NPH swab | 61-year-old female with acute weakness in both legs and severe fatigue, progressive within 1 day (no respiratory symptoms or fever) with positive RT-PCR for SARS-CoV-2 in NPH swab. Nerve conduction studies (day 5) showed delayed distal latencies and absent F waves in early course. Together, the case was suggestive of GBS. |
| Zhou | Case report | No | China | 1 | N/A | RT-PCR unclear site | A 75-year-old female with bilateral cerebral infarctions. |
| Zoghi | Case report | No | Iran | 1 | N/A | No proof | 21-year-old male with fever and cough for 2 weeks. Urinary retention, paraparesis in upper limbs and drowsiness. Th8 sensory level. Brain MRI showed bilateral long corticospinal tract lesions in internal capsules extending to the cerebral peduncles and pons on T2-weighted FLAIR as well as hyperintensity of the corpus callosum. MRI of spine with and intramedullar lesion extending over 3 segments. Slight improvement over time. CSF negative for anti-MOG antibodies. Atypical demyelination. Negative PCR for SARS-CoV-2 RNA. |

*Abbreviations: ADEM = acute demyelinating encephalomyelitis; AIDP = acute inflammatory demyelinating polyneuropathy; CADASIL = cerebral autosomal dominant arteriopathy with subcortical infarcts and leukoencephalopathy; CoV = coronavirus; CT = computed tomography; COVID-19 = coronavirus disease 2019; EBV = Epstein–Barr virus; ECMO = extracorporeal membrane oxygenation; EEG = electroencephalogram; FLAIR = fluid attenuated inversion recovery; GBS = Guillain-Barre syndrome; GCS = Glasgow Coma Scale; GFAP = glial fibrillary acidic protein; ICU = intensive care unit; ISH = in situ hybridization; MOG = m* *yelin oligodendrocyte glycoprotein; MRI = magnetic resonance imaging; NPH = nasopharyngeal; OR = odds ratio; PRES = posterior reversible encephalitis syndrome; RT-PCR = reverse transcriptase polymerase chain reaction; SARS = severe acute upper respiratory syndrome*

**Table S8:** Fifteen studies assessed the association of coronavirus 229E and OC43 (potentially other HCoVs) to multiple sclerosis (including optic neuritis).

| **First author** | **Year** | **MS patients** | **Non-MS controls** | **Tissue** | **Method** | **Main findings** |
| --- | --- | --- | --- | --- | --- | --- |
| Burks | 1980 | 13 | 12 | Brain | PCR | Coronavirus (unclear species) detected in 2/13 MS patients (15%) but none of the controls. |
| Madden | 1981 | unclear | unclear | Serum | ELISA | No difference in seroprevalence of OC43 or 229E antibodies in MS patients vs controls (OC43: 56.4% MS, 73.7% Ctrl - 229E: 50.9% MS, 47.3% Ctrl). |
| Salmi* | 1982 | 27 | unclear | CSF | Radio-immuneassay | Higher prevalence of antibodies against OC43 and 229E coronaviruses in MS patients (41% [9/22] and 26% [7/27], respectively) compared to control patients (0%) in CSF. |
| Hovanec | 1983 | 90 | 148 | Serum | ELISA | No difference in seroprevalence for OC43 or 229E coronavirus between MS patients and controls (229E: 50% MS patients, 57% controls, OC43: 93% MS patients, 96% controls). |
| Sorensen | 1986 | 5 | 0 | Brain | Unclear | Autopsy brain were negative for OC43 RNA. |
| Johnson-Lussenburg | 1987 | 39 | 39 | Serum | ELISA | No differences in antibody titers between MS patients and controls in serum. |
| Fleming | 1987 | 21 | 21 | Serum, CSF | ELISA | No difference in antibody titers to coronaviruses OC43 and 229E in CSF and serum between MS and control samples. |
| Alperovitch | 1991 | 57 | 59 | Serum | ELISA | Coronavirus (unclear species) titers not different between MS patients and controls. |
| Stewart | 1992 | 11 | 11 | Brain (gray matter) | RT-PCR | 4/11 MS patients (36%) with 229E RNA in brain compared to 0/11 controls. OC43 RNA was neither detectable in MS patients nor in controls. |
| Murray | 1992 | 22 | 21 | Brain | ISH, IHC | MS patients with higher prevalence of OC43 RNA in brain tissue (5/12, 42%) compared to controls (0/11, 0%). No difference for 229E. Plaques of two progressive MS with OC43 RNA and antigen. |
| Cristallo* | 1997 | 20 | 10 | CSF | RT-PCR | 100% of MS patients and 90% of controls with OC43 RNA in CSF. 35% of MS patients and 20% of controls with 229E RNS in CSF. |
| Dessau | 1999 | 37 (optic neuritis) | 15 | CSF | RT-PCR | No difference in 229E or OC43 RNA in optic neuritis patients versus controls in CSF (4/37 [11%] optic neuritis vs. 1/15 [7%] controls and 0% optic neuritis/controls, respectively). |
| Arbour | 2000 | 39 | 25 | Brain | RT-PCR, ISH | OC43 RNA more common in MS brains compared to control brains (35.7% vs 13.7%), confirmed outside blood vessel using in situ hybridization. No difference between MS and controls for 229E. |
| Dessau | 2001 | 25 | 36 | Brain | RT-PCR | No difference in 229E or OC43 RNA in MS patients versus controls in brain tissue (pooled 7/19 [37%] MS patients and 10/34 [29%] controls). |
| Kriesel | 2004 | 16 | unclear | Nasal mucosa | RT-PCR | Coronaviruses (among other viruses) were not detected in nasal swabs of MS patients. |

*Abbreviations: HCoV, human coronavirus; ISH, in situ hybridization; MS = Multiple Sclerosis; RT-PCR, reverse transcriptase polymerase chain reaction. *Only abstract retrievable.*
