# Supplementary material for: Neurological manifestations of coronavirus infections – a systematic review
Source: Ann Clin Transl Neurol. 2020 Aug 27;7(10):2057–71. doi: 10.1002/acn3.51166 (PMC7461163; doi:10.1002/acn3.51166)
Supplement: Supplementary file 2 — Data S1. Supplementary search string for medical data bases. [file ACN3-7-2057-s002.docx]

**Supplementary reference list**

In alphabetical order

1. Abdel-Mannan O, Eyre M, Löbel U, et al. Neurologic and Radiographic Findings Associated With COVID-19 Infection in Children. JAMA Neurol. 2020 Jul 1.

2. Abdelnour L, Eltahir Abdalla M, Babiker S. COVID 19 infection presenting as motor peripheral neuropathy. J Formos Med Assoc. 2020 2020;119(6):1119-20.

3. Abdi S, Ghorbani A, Fatehi F. The association of SARS-CoV-2 infection and acute disseminated encephalomyelitis without prominent clinical pulmonary symptoms. J Neurol Sci. 2020 2020;416:117001.

4. Abdulkadir T, ÜNLÜBAŞ Y, ALEMDAR M, AKYÜZ E. Coexistence of Covid-19 and Acute Ischemic Stroke Report of Four Cases. Journal of Clinical Neuroscience. 2020.

5. Acharya S, Diamond M, Anwar S, Glaser A, Tyagi P. Unique case of central retinal artery occlusion secondary to COVID-19 disease. IDCases. 2020 2020;21:e00867.

6. Afshar H, Yassin Z, Kalantari S, et al. Evolution and resolution of brain involvement associated with SARS- CoV2 infection: A close Clinical - Paraclinical follow up study of a case. Mult Scler Relat Disord. 2020 May 21;43:102216.

7. Agarwal A, Vishnu VY, Vibha D, et al. Intracerebral Hemorrhage and SARS-CoV-2: Association or Causation. Ann Indian Acad Neurol. 2020 2020;23(3):261-4.

8. Agosti E, Giorgianni A, D’Amore F, Vinacci G, Balbi S, Locatelli D. Is Guillain-Barrè syndrome triggered by SARS-CoV-2? Case report and literature review. Neurological Sciences. 2020 2020.

9. Al Saiegh F, Ghosh R, Leibold A, et al. Status of SARS-CoV-2 in cerebrospinal fluid of patients with COVID-19 and stroke. Journal of Neurology, Neurosurgery & Psychiatry. 2020.

10. Alberti P, Beretta S, Piatti M, et al. Guillain-Barré syndrome related to COVID-19 infection. Neurology-Neuroimmunology Neuroinflammation. 2020;7(4).

11. Algahtani H, Subahi A, Shirah B. Neurological Complications of Middle East Respiratory Syndrome Coronavirus: A Report of Two Cases and Review of the Literature. Case Rep Neurol Med. 2016 2016;2016:3502683.

12. Al-Hameed FM. Spontaneous intracranial hemorrhage in a patient with Middle East respiratory syndrome corona virus. Saudi Med J. Feb;38(2):196-200.

13. AlKetbi R, AlNuaimi D, AlMulla M, et al. Acute myelitis as a neurological complication of Covid-19: A case report and MRI findings. Radiology Case Reports. 2020 2020;15(9):1591-5.

14. Al-Olama M, Rashid A, Garozzo D. COVID-19-associated meningoencephalitis complicated with intracranial hemorrhage: a case report. Acta Neurochir (Wien). 2020 2020;162(7):1495-9.

15. Alpérovitch A, Berr C, Cambon-Thomsen A, et al. Viral antibody titers, immunogenetic markers, and their interrelations in multiple sclerosis patients and controls. Hum Immunol. Jun;31(2):94-9.

16. Ameres M, Brandstetter S, Toncheva AA, et al. Association of neuronal injury blood marker neurofilament light chain with mild-to-moderate COVID-19. Journal of Neurology. 2020 2020.

17. Andrea A, Luana B, Silvia DM, Erika S, Massimo DS. New clinical manifestation of COVID-19 related Guillain-Barrè syndrome highly responsive to intravenous immunoglobulins: two Italian cases. Neurological Sciences. 2020 2020.

18. Andriuta D, Roger PA, Thibault W, et al. COVID-19 encephalopathy: detection of antibodies against SARS-CoV-2 in CSF. J Neurol. 2020 2020:1-2.

19. Anzalone N, Castellano A, Scotti R, et al. Multifocal laminar cortical brain lesions: a consistent MRI finding in neuro-COVID-19 patients. Journal of Neurology. 2020 2020.

20. Arabi YM, Harthi A, Hussein J, et al. Severe neurologic syndrome associated with Middle East respiratory syndrome corona virus (MERS-CoV). Infection. Aug;43(4):495-501.

21. Aragão M, Leal MC, Cartaxo Filho OQ, Fonseca TM, Valença MM. Anosmia in COVID-19 Associated with Injury to the Olfactory Bulbs Evident on MRI. AJNR Am J Neuroradiol. 2020 2020.

22. Arbour N, Day R, Newcombe J, Talbot PJ. Neuroinvasion by human respiratory coronaviruses. J Virol. Oct;74(19):8913-21.

23. Arca KN, Starling AJ. Treatment-Refractory Headache in the Setting of COVID-19 Pneumonia: Migraine or Meningoencephalitis? Case Report. SN Comprehensive Clinical Medicine. 2020 2020.

24. Arnaud S, Budowski C, Ng Wing Tin S, Degos B. Post SARS-CoV-2 Guillain-Barré syndrome. Clin Neurophysiol. 2020 2020;131(7):1652-4.

25. Ashrafi F, Zali A, Ommi D, et al. COVID-19-related strokes in adults below 55 years of age: a case series. Neurol Sci. 2020 2020:1-5.

26. Atere M, Singh S, Arora K, et al. COVID-19: The Case of Three Patients with the Same Diagnosis but Different Clinical and Laboratory Features. Case Rep Med. 2020 2020;2020:9185041.

27. Avula A, Nalleballe K, Narula N, et al. COVID-19 presenting as stroke. Brain, behavior, and immunity. 2020.

28. Balestrino R, Rizzone M, Zibetti M, et al. Onset of Covid-19 with impaired consciousness and ataxia: a case report. Journal of Neurology. 2020 2020.

29. Balloy G, Mahé P-J, Leclair-Visonneau L, et al. Non-lesional status epilepticus in a patient with coronavirus disease 2019. Clinical Neurophysiology. 2020.

30. Bao Y, Lin SY, Cheng ZH, et al. Clinical Features of COVID-19 in a Young Man with Massive Cerebral Hemorrhage—Case Report. SN Comprehensive Clinical Medicine. 2020 2020;2(6):703-9.

31. Barrios-López JM, Rego-García I, Muñoz Martínez C, et al. Ischaemic stroke and SARS-CoV-2 infection: A causal or incidental association? Neurologia. 2020 2020;35(5):295-302.

32. Beach SR, Praschan NC, Hogan C, et al. Delirium in COVID-19: A case series and exploration of potential mechanisms for central nervous system involvement. Gen Hosp Psychiatry. 2020 2020;65:47-53.

33. Beltrán-Corbellini Á, Chico-García JL, Martínez-Poles J, et al. Acute-onset smell and taste disorders in the context of Covid-19: a pilot multicenter PCR-based case-control study. Eur J Neurol. Apr-22.

34. Belvis R. Headaches During COVID-19: My Clinical Case and Review of the Literature. Headache. 2020 2020.

35. Benameur K, Agarwal A, Auld SC, et al. Encephalopathy and Encephalitis Associated with Cerebrospinal Fluid Cytokine Alterations and Coronavirus Disease, Atlanta, Georgia, USA, 2020. Emerg Infect Dis. 2020 2020;26(9).

36. Benger M, Williams O, Siddiqui J, Sztriha L. Intracerebral haemorrhage and COVID-19: Clinical characteristics from a case series. Brain, Behavior, and Immunity. 2020 2020.

37. Benussi A, Pilotto A, Premi E, et al. Clinical characteristics and outcomes of inpatients with neurologic disease and COVID-19 in Brescia, Lombardy, Italy. Neurology. 2020 2020.

38. Bernard‐Valnet R, Pizzarotti B, Anichini A, et al. Two patients with acute meningo‐encephalitis concomitant to SARS‐CoV‐2 infection. European Journal of Neurology. 2020.

39. Beyrouti R, Adams ME, Benjamin L, et al. Characteristics of ischaemic stroke associated with COVID-19. Journal of Neurology, Neurosurgery & Psychiatry. 2020.

40. Bhatta S, Sayed A, Ranabhat B, Bhatta RK, Acharya Y. New-Onset Seizure as the Only Presentation in a Child With COVID-19. Cureus. 2020 2020;12(6):6.

41. Bigaut K, Mallaret M, Baloglu S, et al. Guillain-Barré syndrome related to SARS-CoV-2 infection. Neurology(R) neuroimmunology & neuroinflammation. 2020 2020;7(5).

42. Bodro M, Compta Y, Llansó L, et al. Increased CSF levels of IL-1β, IL-6, and ACE in SARS-CoV-2-associated encephalitis. Neurology(R) neuroimmunology & neuroinflammation. 2020 2020;7(5).

43. Bonardel C, Bonnerot M, Ludwig M, et al. Bilateral Posterior Cerebral Artery Territory Infarction in a SARS-Cov-2 Infected Patient: discussion about an unusual case. Journal of Stroke and Cerebrovascular Diseases. 2020 2020;29(9).

44. Bracaglia M, Naldi I, Govoni A, Brillanti Ventura D, De Massis P. Acute inflammatory demyelinating polyneuritis in association with an asymptomatic infection by SARS-CoV-2. J Neurol. 2020 2020:1-3.

45. Brüggemann R, Gietema H, Jallah B, Ten Cate H, Stehouwer C, Spaetgens B. Arterial and venous thromboembolic disease in a patient with COVID-19: A case report. Thromb Res. 2020 2020;191:153-5.

46. Brun G, Hak JF, Coze S, et al. COVID-19-White matter and globus pallidum lesions: Demyelination or small-vessel vasculitis? Neurology(R) neuroimmunology & neuroinflammation. 2020 Jul;7(4).

47. Burks JS, Devald BL, Jankovsky LD, Gerdes JC. 2 CORONAVIRUSES ISOLATED FROM CENTRAL NERVOUS-SYSTEM TISSUE OF 2 MULTIPLE-SCLEROSIS PATIENTS. Science. 1980 1980;209(4459):933-4.

48. Butt I, Sawlani V, Geberhiwot T. Prolonged Confusional state as first manifestation of COVID-19. Annals of Clinical and Translational Neurology. 2020 2020.

49. Caamano DSJ, Beato RA. Facial diplegia, a possible atypical variant of Guillain-Barre Syndrome as a rare neurological complication of SARS-CoV-2. Journal of Clinical Neuroscience. 2020 2020;77:230-2.

50. Cabeça TK, Bellei N. Human coronavirus NL-63 infection in a Brazilian patient suspected of H1N1 2009 influenza infection: description of a fatal case. J Clin Virol. Jan;53(1):82-4.

51. Camdessanche JP, Morel J, Pozzetto B, Paul S, Tholance Y, Botelho-Nevers E. COVID-19 may induce Guillain-Barré syndrome. Rev Neurol (Paris). 2020 2020;176(6):516-8.

52. Cantador E, Núñez A, Sobrino P, et al. Incidence and consequences of systemic arterial thrombotic events in COVID-19 patients. Journal of Thrombosis and Thrombolysis. 2020 2020.

53. Capelli M, Gatti P. Anosmia and COVID-19 in south Lombardy: description of the first cases series in Europe. B-Ent. 2020 2020;16(1):86-90.

54. Carfì A, Bernabei R, Landi F. Persistent Symptoms in Patients After Acute COVID-19. JAMA.

55. Carignan A, Valiquette L, Grenier C, et al. Anosmia and dysgeusia associated with SARS-CoV-2 infection: an age-matched case-control study. Cmaj. 2020 2020;192(26):E702-e7.

56. Carman KB, Calik M, Karal Y, et al. Viral etiological causes of febrile seizures for respiratory pathogens (EFES Study). Hum Vaccin Immunother. 2019 2019;15(2):496-502.

57. Carroll E, Lewis A. Catastrophic Intracranial Hemorrhage in Two Critically Ill Patients with COVID-19. Neurocritical Care. 2020 2020.

58. Cavalcanti DD, Raz E, Shapiro M, et al. Cerebral Venous Thrombosis Associated with COVID-19. AJNR Am J Neuroradiol. 2020 2020.

59. Cebrián J, Gonzalez-Martinez A, García-Blanco MJ, et al. Headache and impaired consciousness level associated with SARS-CoV-2 in CSF: A case report. Neurology. 2020 2020.

60. Cecchetti G, Vabanesi M, Chieffo R, et al. Cerebral involvement in COVID-19 is associated with metabolic and coagulation derangements: an EEG study. Journal of Neurology. 2020 2020.

61. Cerasti D, Ormitti F, Pardatscher S, et al. Multiple Acute Ischemic Strokes in a COVID-19 Patient: a Case Report. SN Comprehensive Clinical Medicine. 2020 2020.

62. Chan JL, Ebadi H, Sarna JR. Guillain-Barré syndrome with facial diplegia related to SARS-CoV-2 infection. The Canadian journal of neurological sciences Le journal canadien des sciences neurologiques. 2020 2020:1-10.

63. Chao CC, Tsai LK, Chiou YH, et al. Peripheral nerve disease in SARS: report of a case. Neurology. Dec-23;61(12):1820-1.

64. Chaumont H, Etienne P, Roze E, Couratier C, Roger PM, Lannuzel A. Acute meningoencephalitis in a patient with COVID-19. Rev Neurol (Paris). 2020 2020;176(6):519-21.

65. Chaumont H, San-Galli A, Martino F, et al. Mixed central and peripheral nervous system disorders in severe SARS-CoV-2 infection. J Neurol. 2020 2020:1-7.

66. Chen C, Chen M, Cheng C, et al. A special symptom of olfactory dysfunction in coronavirus disease 2019: report of three cases. J Neurovirol. 2020 2020;26(3):456-8.

67. Chen N, Zhou M, Dong X, et al. Epidemiological and clinical characteristics of 99 cases of 2019 novel coronavirus pneumonia in Wuhan, China: a descriptive study. The Lancet. 2020;395(10223):507-13.

68. Chen T, Hanna J, Walsh EE, Falsey AR, Laguio-Vila M, Lesho E. Syncope, Near Syncope, or Nonmechanical Falls as a Presenting Feature of COVID-19. Ann Emerg Med. 2020 2020;76(1):115-7.

69. Chen T, Wu D, Chen H, et al. Clinical characteristics of 113 deceased patients with coronavirus disease 2019: retrospective study. Bmj. Mar-26;368:m1091.

70. Chiesa-Estomba CM, Lechien JR, Radulesco T, et al. Patterns of smell recovery in 751 patients affected by the COVID-19 outbreak. Eur J Neurol. 2020 2020.

71. Chougar L, Mathon B, Weiss N, Degos V, Shor N. Atypical Deep Cerebral Vein Thrombosis with Hemorrhagic Venous Infarction in a Patient Positive for COVID-19. AJNR Am J Neuroradiol. 2020 2020.

72. Chougar L, Shor N, Weiss N, et al. Retrospective Observational Study of Brain Magnetic Resonance Imaging Findings in Patients with Acute SARS-CoV-2 Infection and Neurological Manifestations. Radiology. 2020 2020:202422.

73. Co COC, Yu JRT, Laxamana LC, David-Ona DIA. Intravenous Thrombolysis for Stroke in a COVID-19 Positive Filipino Patient, a Case Report. J Clin Neurosci. 2020 2020;77:234-6.

74. Coen M, Jeanson G, Culebras Almeida LA, et al. Guillain-Barré syndrome as a complication of SARS-CoV-2 infection. Brain Behav Immun. 2020 2020;87:111-2.

75. Coolen T, Lolli V, Sadeghi N, et al. Early postmortem brain MRI findings in COVID-19 non-survivors. Neurology. 2020 2020.

76. Craen Alexandra LG, Ganti Latha Novel Coronavirus Disease 2019 and Subarachnoid Hemorrhage: A Case Report Cureus. 2020.

77. Cristallo A, Gambaro F, Biamonti G, Ferrante P, Battaglia M, Cereda PM. Human coronavirus polyadenylated RNA sequences in cerebrospinal fluid from multiple sclerosis patients. New Microbiol. Apr;20(2):105-14.

78. D’Anna L, Kwan J, Brown Z, et al. Characteristics and clinical course of Covid-19 patients admitted with acute stroke. Journal of Neurology. 2020 2020.

79. Dakay K, Kaur G, Gulko E, et al. Reversible cerebral vasoconstriction syndrome and dissection in the setting of COVID-19 infection. Journal of Stroke and Cerebrovascular Diseases. 2020 2020;29(9).

80. de Freitas Ferreira ACA, Romão TT, Y SIM, Pupe C, Nascimento OJM. COVID-19 and herpes zoster co-infection presenting with trigeminal neuropathy. European Journal of Neurology. 2020 2020.

81. De Stefano P, Nencha U, De Stefano L, Mégevand P, Seeck M. Focal EEG changes indicating critical illness associated cerebral microbleeds in a Covid-19 patient. Clin Neurophysiol Pract. 2020 2020;5:125-9.

82. Deliwala S, Abdulhamid S, Abusalih MF, Al-Qasmi MM, Bachuwa G. Encephalopathy as the Sentinel Sign of a Cortical Stroke in a Patient Infected With Coronavirus Disease-19 (COVID-19). Cureus. 2020 2020;12(5):e8121.

83. Demirci Otluoglu G, Yener U, Demir MK, Yilmaz B. Encephalomyelitis associated with Covid-19 infection: case report. British journal of neurosurgery. 2020 2020:1-3.

84. Dessau RB, Lisby G, Frederiksen JL. Coronaviruses in spinal fluid of patients with acute monosymptomatic optic neuritis. Acta Neurol Scand. Aug;100(2):88-91.

85. Dessau RB, Lisby G, Frederiksen JL. Coronaviruses in brain tissue from patients with multiple sclerosis. Acta Neuropathol. Jun;101(6):601-4.

86. Diaz-Segarra N, Edmond A, Kunac A, Yonclas P. COVID-19 Ischemic Strokes as an Emerging Rehabilitation Population: A Case Series. Am J Phys Med Rehabil. 2020 2020.

87. Ding Y, Wang H, Shen H, et al. The clinical pathology of severe acute respiratory syndrome (SARS): a report from China. J Pathol. Jul;200(3):282-9.

88. Dixon L, Varley J, Gontsarova A, et al. COVID-19-related acute necrotizing encephalopathy with brain stem involvement in a patient with aplastic anemia. Neurology(R) neuroimmunology & neuroinflammation. 2020 2020;7(5).

89. Dogan L, Kaya D, Sarikaya T, et al. Plasmapheresis treatment in COVID-19–related autoimmune meningoencephalitis: Case series. Brain, Behavior, and Immunity. 2020.

90. Dogra S, Jain R, Cao M, et al. Hemorrhagic stroke and anticoagulation in COVID-19. Journal of Stroke and Cerebrovascular Diseases. 2020 2020;29(8).

91. Domingues RB, Mendes-Correa MC, Leite F, et al. First case of SARS-COV-2 sequencing in cerebrospinal fluid of a patient with suspected demyelinating disease. Journal of Neurology.3.

92. Dominguez SR, Robinson CC, Holmes KV. Detection of four human coronaviruses in respiratory infections in children: a one-year study in Colorado. J Med Virol. Sep;81(9):1597-604.

93. Dugue R, Cay-Martínez KC, Thakur KT, et al. Neurologic manifestations in an infant with COVID-19. Neurology. 2020 2020;94(24):1100-2.

94. Dumitrascu OM, Volod O, Bose S, Wang Y, Biousse V, Lyden PD. Acute ophthalmic artery occlusion in a COVID-19 patient on apixaban. Journal of Stroke and Cerebrovascular Diseases. 2020 2020;29(8).

95. Duong L, Xu P, Liu A. Meningoencephalitis without respiratory failure in a young female patient with COVID-19 infection in Downtown Los Angeles, early April 2020. Brain Behav Immun. 2020 2020;87:33.

96. Edler C, Schröder AS, Aepfelbacher M, et al. Dying with SARS-CoV-2 infection-an autopsy study of the first consecutive 80 cases in Hamburg, Germany. Int J Legal Med. 2020 2020;134(4):1275-84.

97. Efe IE, Aydin OU, Alabulut A, Celik O, Aydin K. COVID-19-Associated Encephalitis Mimicking Glial Tumor. World Neurosurg. 2020 2020;140:46-8.

98. El Otmani H, El Moutawakil B, Rafai M-A, et al. Covid-19 and Guillain-Barré syndrome: More than a coincidence! Revue Neurologique. 2020.

99. Elkhouly A, Kaplan AC. Noteworthy Neurological Manifestations Associated With COVID-19 Infection. Cureus. 2020 2020;12(7):5.

100. Escalada Pellitero S, Garriga Ferrer-Bergua L. A patient with neurological manifestations as unique presentation of SARS-CoV-2 infection. Neurologia. 2020 2020.

101. Escalard S, Maïer B, Redjem H, et al. Treatment of Acute Ischemic Stroke due to Large Vessel Occlusion With COVID-19: Experience From Paris. Stroke. 2020 2020:Strokeaha120030574.

102. Espíndola OM, Siqueira M, Soares CN, et al. Patients with COVID-19 and neurological manifestations show undetectable SARS-CoV-2 RNA levels in the cerebrospinal fluid. Int J Infect Dis. 2020 Jun 4.

103. Espinosa PS, Rizvi Z, Sharma P, Hindi F, Filatov A. Neurological Complications of Coronavirus Disease (COVID-19): Encephalopathy, MRI Brain and Cerebrospinal Fluid Findings: Case 2. Cureus. 2020 May 2;12(5):e7930.

104. Falcone MM, Rong AJ, Salazar H, Redick DW, Falcone S, Cavuoto KM. Acute abducens nerve palsy in a patient with the novel coronavirus disease (COVID-19). J aapos. 2020 2020.

105. Fara MG, Stein LK, Skliut M, Morgello S, Fifi JT, Dhamoon MS. Macrothrombosis and stroke in patients with mild Covid-19 infection. Journal of thrombosis and haemostasis : JTH. 2020 2020.

106. Farhadian S, Farhadian S, Glick LR, et al. Acute encephalopathy with elevated CSF inflammatory markers as the initial presentation of COVID-19. BMC Neurology. 2020 2020;20(1).

107. Farzi MA, Ayromlou H, Jahanbakhsh N, Bavil PH, Janzadeh A, Shayan FK. Guillain-Barré syndrome in a patient infected with SARS-CoV-2, a case report. J Neuroimmunol. 2020 2020;346:577294.

108. Fasano A, Cavallieri F, Canali E, Valzania F. First motor seizure as presenting symptom of SARS-CoV-2 infection. Neurol Sci. 2020 2020;41(7):1651-3.

109. Faucher A, Rey PA, Aguadisch E, Degos B. Isolated post SARS-CoV-2 diplopia. Journal of Neurology. 2020 2020.

110. Fazzini E, Fleming J, Fahn S. Cerebrospinal fluid antibodies to coronavirus in patients with Parkinson's disease. Mov Disord. 1992 1992;7(2):153-8.

111. Fernández-Domínguez J, Ameijide-Sanluis E, García-Cabo C, García-Rodríguez R, Mateos V. Miller–Fisher-like syndrome related to SARS-CoV-2 infection (COVID 19). Journal of Neurology. 2020 2020.

112. Filatov A, Sharma P, Hindi F, Espinosa PS. Neurological Complications of Coronavirus Disease (COVID-19): Encephalopathy. Cureus. Mar-21;12(3):e7352.

113. Filatov A, Sharma P, Hindi F, Espinosa PS. Neurological complications of coronavirus disease (COVID-19): encephalopathy. Cureus. 2020;12(3).

114. Finatti F, Pigato G, Pavan C, Toffanin T, Favaro A. Psychosis in Patients in COVID-19-Related Quarantine: A Case Series. Prim Care Companion CNS Disord. 2020 2020;22(3).

115. Fischer D, Threlkeld ZD, Bodien YG, et al. Intact Brain Network Function in an Unresponsive Patient with COVID-19. Ann Neurol. 2020 2020.

116. Fitsiori A, Pugin D, Thieffry C, Lalive P, Vargas MI. Unusual Microbleeds in Brain MRI of Covid-19 Patients. J Neuroimaging. 2020 2020.

117. Fleming JO, el Zaatari FA, Gilmore W, et al. Antigenic assessment of coronaviruses isolated from patients with multiple sclerosis. Arch Neurol. Jun;45(6):629-33.

118. Franceschi AM, Ahmed O, Giliberto L, Castillo M. Hemorrhagic Posterior Reversible Encephalopathy Syndrome as a Manifestation of COVID-19 Infection. AJNR Am J Neuroradiol. 2020 2020;41(7):1173-6.

119. Franceschi AM, Arora R, Wilson R, Giliberto L, Libman RB, Castillo M. Neurovascular Complications in COVID-19 Infection: Case Series. AJNR Am J Neuroradiol. 2020 2020.

120. Frank CHM, Almeida TVR, Marques EA, et al. Guillain-Barré Syndrome Associated with SARS-CoV-2 Infection in a Pediatric Patient. J Trop Pediatr. 2020 2020.

121. Freni F, Meduri A, Gazia F, et al. Symptomatology in head and neck district in coronavirus disease (COVID-19): A possible neuroinvasive action of SARS-CoV-2. Am J Otolaryngol. 2020 2020;41(5):102612.

122. Galanopoulou AS, Ferastraoaru V, Correa DJ, et al. EEG findings in acutely ill patients investigated for SARS-CoV-2/COVID-19: A small case series preliminary report. Epilepsia Open. 2020 2020;5(2):314-24.

123. Galougahi MK, Ghorbani J, Bakhshayeshkaram M, Naeini AS, Haseli S. Olfactory Bulb Magnetic Resonance Imaging in SARS-CoV-2-Induced Anosmia: The First Report. Acad Radiol. Apr-11.

124. Gane SB, Kelly C, Hopkins C. Isolated sudden onset anosmia in COVID-19 infection. A novel syndrome? Rhinology. 2020 2020;58(3):299-301.

125. Garaci F, Di Giuliano F, Picchi E, Da Ros V, Floris R. Venous cerebral thrombosis in COVID-19 patient. J Neurol Sci. 2020 2020;414:116871.

126. Gautier JF, Ravussin Y. A New Symptom of COVID-19: Loss of Taste and Smell. Obesity (Silver Spring). 2020 2020;28(5):848.

127. Ghiasvand F, Ghadimi M, Ghadimi F, Safarpour S, Hosseinzadeh R, SeyedAlinaghi S. Symmetrical polyneuropathy in coronavirus disease 2019 (COVID-19). IDCases. 2020 2020;21.

128. Ghosh R, Dubey S, Kanti Ray B, Chatterjee S, Benito-León J. COVID-19 Presenting With Thalamic Hemorrhage Unmasking Moyamoya Angiopathy. Can J Neurol Sci. 2020 2020:1-3.

129. Gilani S, Roditi R, Naraghi M. COVID-19 and anosmia in Tehran, Iran. Med Hypotheses. Apr-23;141:109757.

130. Gill I, Chan S, Fitzpatrick D. COVID-19-associated pulmonary and cerebral thromboembolic disease. Radiology Case Reports. 2020 2020;15(8):1242-9.

131. Giorgianni A, Vinacci G, Agosti E, et al. Transient acute-onset tetraparesis in a COVID-19 patient. Spinal Cord. 2020 2020.

132. Goh Y, Beh DLL, Makmur A, Somani J, Chan ACY. Pearls and Oy-sters: Facial nerve palsy as a neurological manifestation of Covid-19 infection. Neurology. 2020 2020.

133. Goldberg MF, Goldberg MF, Cerejo R, Tayal A. Cerebrovascular Disease in COVID-19. American Journal of Neuroradiology. 2020.

134. Gómez-Iglesias P, Porta-Etessam J, Montalvo T, et al. An Online Observational Study of Patients With Olfactory and Gustory Alterations Secondary to SARS-CoV-2 Infection. Front Public Health. 2020 2020;8:243.

135. González‐Pinto T, Luna‐Rodríguez A, Moreno‐Estébanez A, Agirre‐Beitia G, Rodríguez‐Antigüedad A, Ruiz‐Lopez M. Emergency Room Neurology in times of COVID‐19: Malignant Ischemic Stroke and SARS‐COV2 Infection. European Journal of Neurology. 2020.

136. Gu J, Gong E, Zhang B, et al. Multiple organ infection and the pathogenesis of SARS. J Exp Med. Aug-1;202(3):415-24.

137. Guillan M, Villacieros-Alvarez J, Bellido S, et al. Unusual simultaneous cerebral infarcts in multiple arterial territories in a COVID-19 patient. Thromb Res. 2020 2020;193:107-9.

138. Gulko E, Gomes W, Ali S, Al-Mufti F, Mehta H. Acute Common Carotid Artery Bifurcation Thrombus: An Emerging Pattern of Acute Strokes in Patients with COVID-19? AJNR American journal of neuroradiology. 2020 2020.

139. Gutiérrez-Ortiz C, Méndez A, Rodrigo-Rey S, et al. Miller Fisher Syndrome and polyneuritis cranialis in COVID-19. Neurology. Apr-17.

140. Haddad S, Tayyar R, Risch L, et al. Encephalopathy and seizure activity in a COVID-19 well controlled HIV patient. IDCases. 2020 2020;21.

141. Haddadi Kaveh GR, Shafizad Misagh Basal Ganglia Involvement and Altered Mental Status: A Unique Neurological Manifestation of Coronavirus Disease 2019. Cureus. 2020.

142. Haldrup M, Johansen MI, Fjaeldstad AW. [Anosmia and ageusia as primary symptoms of COVID-19]. Ugeskr Laeger. 2020 2020;182(18).

143. Hanafi R, Roger PA, Perin B, et al. COVID-19 Neurologic Complication with CNS Vasculitis-Like Pattern. AJNR Am J Neuroradiol. 2020 2020.

144. Hayashi M, Sahashi Y, Baba Y, Okura H, Shimohata T. COVID-19-associated mild encephalitis/encephalopathy with a reversible splenial lesion. Journal of the Neurological Sciences. 2020 2020;415.

145. Helbok R, Beer R, Löscher W, et al. Guillain-Barré syndrome in a patient with antibodies against SARS-COV-2. Eur J Neurol. 2020 2020.

146. Helms J, Kremer S, Merdji H, et al. Neurologic Features in Severe SARS-CoV-2 Infection. N Engl J Med. Apr-15.

147. Heman-Ackah SM, Su YS, Spadola M, et al. Neurologically Devastating Intraparenchymal Hemorrhage in COVID-19 Patients on Extracorporeal Membrane Oxygenation: A Case Series. Neurosurgery. 2020 2020.

148. Hemasian H, Ansari B. First case of Covid-19 presented with cerebral venous thrombosis: A rare and dreaded case. Rev Neurol (Paris). 2020 2020;176(6):521-3.

149. Hepburn M, Mullaguri N, George P, et al. Acute Symptomatic Seizures in Critically Ill Patients with COVID-19: Is There an Association? Neurocrit Care. 2020 2020:1-5.

150. Hernández-Fernández F, Valencia HS, Barbella-Aponte RA, et al. Cerebrovascular disease in patients with COVID-19: neuroimaging, histological and clinical description. Brain : a journal of neurology. 2020 Jul 9.

151. Hjelmesæth J, Skaare D. Loss of smell or taste as the only symptom of COVID-19. Tidsskrift for Den norske legeforening. 2020.

152. Homma Y, Watanabe M, Inoue K, Moritaka T. Coronavirus Disease-19 Pneumonia with Facial Nerve Palsy and Olfactory Disturbance. Intern Med. 2020 2020;59(14):1773-5.

153. Hopkins C, Surda P, Kumar N. Presentation of new onset anosmia during the COVID-19 pandemic. Rhinology. 2020 2020;58(3):295-8.

154. Hopkins C, Surda P, Whitehead E, Kumar BN. Early recovery following new onset anosmia during the COVID-19 pandemic–an observational cohort study. Journal of Otolaryngology-Head & Neck Surgery. 2020;49:1-6.

155. Hornuss D, Lange B, Schröter N, Rieg S, Kern WV, Wagner D. Anosmia in COVID-19 patients. Clinical Microbiology and Infection. 2020 2020.

156. Hosseini AA, Shetty AK, Sprigg N, Auer DP, Constantinescu CS. Delirium as a presenting feature in COVID-19: Neuroinvasive infection or autoimmune encephalopathy? Brain, Behavior, and Immunity. 2020 2020.

157. Hovanec DL, Flanagan TD. Detection of antibodies to human coronaviruses 229E and OC43 in the sera of multiple sclerosis patients and normal subjects. Infect Immun. Jul;41(1):426-9.

158. Hu SS, Yang YJ, Zhu ML, et al. [Effects of underlying cerebrocardiovascular diseases on the incidence of critical conditions and multiple organs dysfunction syndrome in severe acute respiratory syndrome cases]. Zhonghua Yi Xue Za Zhi. Aug-2;84(15):1257-9.

159. Huang C, Wang Y, Li X, et al. Clinical features of patients infected with 2019 novel coronavirus in Wuhan, China. The lancet. 2020;395(10223):497-506.

160. Huang YH, Jiang D, Huang JT. SARS-CoV-2 Detected in Cerebrospinal Fluid by PCR in a Case of COVID-19 Encephalitis. Brain Behav Immun. 2020 2020;87:149.

161. Hung ECW, Chim SSC, Chan PKS, et al. Detection of SARS Coronavirus RNA in the Cerebrospinal Fluid of a Patient with Severe Acute Respiratory Syndrome. Clinical Chemistry. 2003 2003;49(12):2108-9.

162. Hutchins KL, Jansen JH, Comer AD, et al. COVID-19-Associated Bifacial Weakness with Paresthesia Subtype of Guillain-Barré Syndrome. AJNR Am J Neuroradiol. 2020 2020.

163. Hwang CS. Olfactory neuropathy in severe acute respiratory syndrome: report of A case. Acta Neurol Taiwan. Mar;15(1):26-8.

164. Immovilli P, Terracciano C, Zaino D, et al. Stroke in COVID-19 patients—A case series from Italy. International Journal of Stroke. 2020 2020.

165. Jain R, Young M, Dogra S, et al. COVID-19 related neuroimaging findings: A signal of thromboembolic complications and a strong prognostic marker of poor patient outcome. J Neurol Sci. 2020 2020;414:116923.

166. Jevšnik M, Steyer A, Pokorn M, et al. The Role of Human Coronaviruses in Children Hospitalized for Acute Bronchiolitis, Acute Gastroenteritis, and Febrile Seizures: A 2-Year Prospective Study. PLoS One. 2016 2016;11(5):e0155555.

167. Johnson-Lussenburg CM, Zheng Q. Coronavirus and multiple sclerosis: results of a case/control longitudinal serological study. Adv Exp Med Biol. 1987 1987;218:421-9.

168. Joob B, Wiwanitkit V. Alteration of consciousness as initial presentation in COVID-19: Observation. Annals of Indian Academy of Neurology. 2020 2020;23(7):44.

169. Kadono Y, Nakamura Y, Ogawa Y, et al. A case of COVID-19 infection presenting with a seizure following severe brain edema. Seizure. 2020 2020;80:53-5.

170. Kanberg N, Ashton NJ, Andersson LM, et al. Neurochemical evidence of astrocytic and neuronal injury commonly found in COVID-19. Neurology. 2020 2020.

171. Kandemirli SG, Dogan L, Sarikaya ZT, et al. Brain MRI Findings in Patients in the Intensive Care Unit with COVID-19 Infection. Radiology. 2020:201697.

172. Karadaş Ö, Öztürk B, Sonkaya AR. A prospective clinical study of detailed neurological manifestations in patients with COVID-19. Neurol Sci. 2020 2020:1-5.

173. Karimi N, Razavi AS, Rouhani N. Frequent Convulsive Seizures in an Adult Patient with COVID-19: A Case Report. Iranian Red Crescent Medical Journal. 2020 2020;22(3):3.

174. Karimi-Galougahi M, Yousefi-Koma A, Bakhshayeshkaram M, Raad N, Haseli S. 18FDG PET/CT scan reveals hypoactive orbitofrontal cortex in anosmia of COVID-19. Academic Radiology. 2020.

175. Kaya Y, Kara S, Akinci C, Kocaman AS. Transient cortical blindness in COVID-19 pneumonia; a PRES-like syndrome: Case report. J Neurol Sci. 2020 2020;413:116858.

176. Khalifa M, Zakaria F, Ragab Y, et al. Guillain-Barre Syndrome Associated with SARS-CoV-2 Detection and a COVID-19 Infection in a Child. J Pediatric Infect Dis Soc. 2020 2020.

177. Khan M, Ibrahim RHM, Siddiqi SA, et al. COVID-19 and acute ischemic stroke – A case series from Dubai, UAE. International Journal of Stroke. 2020 2020.

178. Khoo A, McLoughlin B, Cheema S, et al. Postinfectious brainstem encephalitis associated with SARS-CoV-2. J Neurol Neurosurg Psychiatry. 2020 2020.

179. Kilinc D, van de Pasch S, Doets AY, Jacobs BC, van Vliet J, Garssen MPJ. Guillain-Barré syndrome after SARS-CoV-2 infection. Eur J Neurol. 2020 2020.

180. Kim C, Kwak Y, Hwang J, Eun MY. Spontaneous Intracerebral Hemorrhage in a Patient with Asymptomatic 2019 Novel Coronavirus Disease. J Clin Neurol. 2020 2020;16(3):515-7.

181. Kim JE, Heo JH, Kim HO, et al. Neurological Complications during Treatment of Middle East Respiratory Syndrome. J Clin Neurol. Jul;13(3):227-33.

182. Kishfy L, Casasola M, Banankhah P, et al. Posterior reversible encephalopathy syndrome (PRES) as a neurological association in severe Covid-19. J Neurol Sci. 2020 2020;414:116943.

183. Klein DE, Libman R, Kirsch C, Arora R. Cerebral venous thrombosis: A typical presentation of COVID-19 in the young. Journal of Stroke and Cerebrovascular Diseases. 2020 2020;29(8).

184. Klok FA, Kruip M, van der Meer NJM, et al. Confirmation of the high cumulative incidence of thrombotic complications in critically ill ICU patients with COVID-19: An updated analysis. Thromb Res. 2020 2020;191:148-50.

185. Kong Z, Wang J, Li T, Zhang Z, Jian J. 2019 novel coronavirus pneumonia with onset of dizziness: a case report. Ann Transl Med. 2020 2020;8(7):506.

186. Kremer S, Lersy F, de Sèze J, et al. Brain MRI Findings in Severe COVID-19: A Retrospective Observational Study. Radiology. 2020 Jun 16:202222.

187. Kriesel JD, White A, Hayden FG, Spruance SL, Petajan J. Multiple sclerosis attacks are associated with picornavirus infections. Multiple Sclerosis. 2004 2004;10(2):145-8.

188. Kulick-Soper CV, McKee JL, Wolf RL, et al. Pearls & Oy-sters: Bilateral globus pallidus lesions in a patient with COVID-19. Neurology. 2020 2020.

189. Kwee RM, Krdzalic J, Fasen B, de Jaegere TMH. CT Scanning in Suspected Stroke or Head Trauma: Is it Worth Going the Extra Mile and Including the Chest to Screen for COVID-19 Infection? AJNR Am J Neuroradiol. 2020 2020;41(7):1165-9.

190. Lang M, Buch K, Li MD, et al. Leukoencephalopathy Associated with Severe COVID-19 Infection: Sequela of Hypoxemia? AJNR Am J Neuroradiol. 2020 2020.

191. Lantos JE, Strauss SB, Lin E. COVID-19-Associated Miller Fisher Syndrome: MRI Findings. AJNR Am J Neuroradiol. 2020 2020;41(7):1184-6.

192. Lascano AM, Epiney JB, Coen M, et al. SARS-CoV-2 and Guillain-Barré syndrome: AIDP variant with favorable outcome. Eur J Neurol. 2020 2020.

193. Lau KK, Yu WC, Chu CM, Lau ST, Sheng B, Yuen KY. Possible central nervous system infection by SARS coronavirus. Emerg Infect Dis. Feb;10(2):342-4.

194. Lau SK, Woo PC, Yip CC, et al. Coronavirus HKU1 and other coronavirus infections in Hong Kong. J Clin Microbiol. Jun;44(6):2063-71.

195. Laurendon T, Radulesco T, Mugnier J, et al. Bilateral transient olfactory bulbs edema during COVID-19-related anosmia. Neurology. 2020 2020.

196. Le Guennec L, Devianne J, Jalin L, et al. Orbitofrontal involvement in a neuroCOVID-19 patient. Epilepsia. 2020 2020.

197. Lechien JR, Cabaraux P, Chiesa-Estomba CM, et al. Objective olfactory evaluation of self-reported loss of smell in a case series of 86 COVID-19 patients. Head Neck. 2020 2020;42(7):1583-90.

198. Lechien JR, Cabaraux P, Chiesa-Estomba CM, et al. Psychophysical Olfactory Tests and Detection of COVID-19 in Patients With Sudden Onset Olfactory Dysfunction: A Prospective Study. Ear Nose Throat J. 2020 2020:145561320929169.

199. Lechien JR, Chiesa-Estomba CM, De Siati DR, et al. Olfactory and gustatory dysfunctions as a clinical presentation of mild-to-moderate forms of the coronavirus disease (COVID-19): a multicenter European study. Eur Arch Otorhinolaryngol. Apr-6:1-11.

200. Lechien JR, Chiesa‐Estomba CM, Place S, et al. Clinical and Epidemiological Characteristics of 1,420 European Patients with mild‐to‐moderate Coronavirus Disease 2019. Journal of Internal Medicine. 2020.

201. Lechien JR, Michel J, Radulesco T, et al. Clinical and Radiological Evaluations of COVID-19 Patients with Anosmia: Preliminary Report. Laryngoscope. 2020 2020.

202. Lee JM, Lee SJ. Olfactory and Gustatory Dysfunction in a COVID-19 Patient with Ankylosing Spondylitis Treated with Etanercept: Case Report. J Korean Med Sci. 2020 2020;35(21):e201.

203. Lee Y, Min P, Lee S, Kim S-W. Prevalence and Duration of Acute Loss of Smell or Taste in COVID-19 Patients. Journal of Korean medical science. 2020;35(18).

204. Leung TW, Wong KS, Hui AC, et al. Myopathic changes associated with severe acute respiratory syndrome: a postmortem case series. Arch Neurol. Jul;62(7):1113-7.

205. Levinson R, Elbaz M, Ben-Ami R, et al. Time course of anosmia and dysgeusia in patients with mild SARS-CoV-2 infection. Infect Dis (Lond). 2020 2020;52(8):600-2.

206. Li CW, Syue LS, Tsai YS, et al. Anosmia and olfactory tract neuropathy in a case of COVID-19. J Microbiol Immunol Infect. 2020 2020.

207. Li J, Long X, Zhu C, et al. A case of COVID-19 pneumonia with cerebral hemorrhage. Thrombosis Research. 2020 2020;193:22-4.

208. Li J, Long X, Zhu C, et al. Olfactory Dysfunction in Recovered Coronavirus Disease 2019 (COVID-19) Patients. Mov Disord. 2020 2020.

209. Li X, Wang Y, Bai Y, et al. PET/MR and PET/CT in a severe COVID-19 patient. European Journal of Nuclear Medicine and Molecular Imaging. 2020 2020.

210. Li Y, Li M, Wang M, et al. Acute cerebrovascular disease following COVID-19: a single center, retrospective, observational study. Stroke Vasc Neurol. 2020 2020.

211. Li YY, Li HP, Fan RY, et al. Coronavirus Infections in the Central Nervous System and Respiratory Tract Show Distinct Features in Hospitalized Children. Intervirology. 2016 2016;59(3):163-9.

212. Liang JW, Reynolds AS, Reilly K, et al. COVID-19 and Decompressive Hemicraniectomy for Acute Ischemic Stroke. Stroke. 2020 2020:Strokeaha120030804.

213. Liguori C, Pierantozzi M, Spanetta M, et al. Subjective neurological symptoms frequently occur in patients with SARS-CoV2 infection. Brain Behav Immun. 2020 2020.

214. Lodigiani C, Iapichino G, Carenzo L, et al. Venous and arterial thromboembolic complications in COVID-19 patients admitted to an academic hospital in Milan, Italy. Thromb Res. 2020 2020;191:9-14.

215. Logmin K, Karam M, Schichel T, Harmel J, Wojtecki L. Non-epileptic seizures in autonomic dysfunction as the initial symptom of COVID-19. Journal of Neurology. 2020 2020.

216. Lu L, Xiong W, Liu D, et al. New-onset acute symptomatic seizure and risk factors in Corona Virus Disease 2019: A Retrospective Multicenter Study. Epilepsia. Apr-18.

217. Lyons S, O'Kelly B, Woods S, et al. Seizure with CSF lymphocytosis as a presenting feature of COVID-19 in an otherwise healthy young man. Seizure. 2020 2020;80:113-4.

218. Madden DL, Wallen WC, Houff SA, et al. Coronavirus antibodies in sera from patients with multiple sclerosis and matched controls. Arch Neurol. Apr;38(4):209-10.

219. Madia F, Merico B, Primiano G, Cutuli SL, De Pascale G, Servidei S. Acute myopathic quadriplegia in COVID-19 patients in the intensive care unit. Neurology. 2020 2020.

220. Mahammedi A, Saba L, Vagal A, et al. Imaging in Neurological Disease of Hospitalized COVID-19 Patients: An Italian Multicenter Retrospective Observational Study. Radiology. 2020 2020:201933.

221. Mahboob S, Boppana SH, Rose NB, Beutler BD, Tabaac BJ. Large vessel stroke and COVID-19: Case report and literature review. eNeurologicalSci. 2020 2020;20:100250.

222. Malentacchi M, Gned D, Angelino V, et al. Concomitant brain arterial and venous thrombosis in a COVID-19 patient. European Journal of Neurology. 2020 2020.

223. Manganelli F, Vargas M, Iovino A, Iacovazzo C, Santoro L, Servillo G. Brainstem involvement and respiratory failure in COVID-19. Neurological Sciences. 2020 2020.

224. Manganotti P, Bellavita G, D'Acunto L, et al. Clinical neurophysiology and cerebrospinal liquor analysis to detect Guillain Barré syndrome and polyneuritis cranialis in COVID-19 patients: a case series. J Med Virol. 2020 2020.

225. Manganotti P, Pesavento V, Buoite Stella A, et al. Miller Fisher syndrome diagnosis and treatment in a patient with SARS-CoV-2. J Neurovirol. 2020 2020:1-2.

226. Mao L, Jin H, Wang M, et al. Neurologic Manifestations of Hospitalized Patients With Coronavirus Disease 2019 in Wuhan, China. JAMA Neurol. Apr-10.

227. Marta-Enguita J, Rubio-Baines I, Gastón-Zubimendi I. Fatal Guillain-Barre syndrome after infection with SARS-CoV-2. Neurología (English Edition). 2020.

228. Matos AR, Quintas-Neves M, Oliveira AI, et al. COVID-19 associated central nervous system vasculopathy. The Canadian journal of neurological sciences Le journal canadien des sciences neurologiques. 2020 2020:1-6.

229. Maurier F, Godbert B, Perrin J. Respiratory Distress in SARS-CoV-2 without Lung Damage: Phrenic Paralysis Should Be Considered in COVID-19 Infection. Eur J Case Rep Intern Med. 2020 2020;7(6):001728.

230. Mawhinney JA, Wilcock C, Haboubi H, Roshanzamir S. Neurotropism of SARS-CoV-2: COVID-19 presenting with an acute manic episode. BMJ Case Rep. 2020 2020;13(6).

231. McAbee GN, Brosgol Y, Pavlakis S, Agha R, Gaffoor M. Encephalitis Associated with COVID-19 Infection in an 11-Year-Old Child. Pediatric Neurology. 2020 2020.

232. Mehrpour M, Shuaib A, Farahani M, et al. Coronavirus disease 2019 and stroke in Iran: a case series and effects on stroke admissions. Int J Stroke. 2020 2020:1747493020937397.

233. Melley LE, Bress E, Polan E. Hypogeusia as the initial presenting symptom of COVID-19. BMJ Case Rep. 2020 2020;13(5).

234. Méndez-Guerrero A, Laespada-García MI, Gómez-Grande A, et al. Acute hypokinetic-rigid syndrome following SARS-CoV-2 infection. Neurology. 2020 2020.

235. Merkler AE, Parikh NS, Mir S, et al. Risk of Ischemic Stroke in Patients With Coronavirus Disease 2019 (COVID-19) vs Patients With Influenza. JAMA Neurol. 2020 2020.

236. Mermelstein S. Acute anosmia from COVID-19 infection. Practical Neurology. 2020.

237. Mirzaee SMM, Gonçalves FG, Mohammadifard M, Tavakoli SM, Vossough A. Focal Cerebral Arteriopathy in a COVID-19 Pediatric Patient. Radiology. 2020 2020:202197.

238. Mohamud AY, Griffith B, Rehman M, et al. Intraluminal Carotid Artery Thrombus in COVID-19: Another Danger of Cytokine Storm? AJNR Am J Neuroradiol. 2020 2020.

239. Morassi M, Bagatto D, Cobelli M, et al. Stroke in patients with SARS-CoV-2 infection: case series. Journal of Neurology.8.

240. Morassi M, Bigni B, Cobelli M, Giudice L, Bnà C, Vogrig A. Bilateral carotid artery dissection in a SARS-CoV-2 infected patient: causality or coincidence? J Neurol. 2020 2020:1-3.

241. Morfopoulou S, Brown JR, Davies EG, et al. Human Coronavirus OC43 Associated with Fatal Encephalitis. N Engl J Med. Aug-4;375(5):497-8.

242. Moriguchi T, Harii N, Goto J, et al. A first case of meningitis/encephalitis associated with SARS-Coronavirus-2. Int J Infect Dis. Apr-3;94:55-8.

243. Moshayedi P, Ryan TE, Mejia LLP, Nour M, Liebeskind DS. Triage of Acute Ischemic Stroke in Confirmed COVID-19: Large Vessel Occlusion Associated With Coronavirus Infection. Frontiers in Neurology. 2020;11.

244. Muhammad S, Petridis A, Cornelius JF, Hänggi D. Letter to editor: Severe brain haemorrhage and concomitant COVID-19 Infection: A neurovascular complication of COVID-19. Brain Behav Immun. 2020 2020;87:150-1.

245. Munz M, Wessendorf S, Koretsis G, et al. Acute transverse myelitis after COVID-19 pneumonia. Journal of Neurology. 2020 2020.

246. Murray RS, Brown B, Brian D, Cabirac GF. Detection of coronavirus RNA and antigen in multiple sclerosis brain. Ann Neurol. May;31(5):525-33.

247. Nepal P, Batchala PP, Songmen S, Parashar K, Sapire J. An unresponsive COVID-19 patient. Emerg Radiol. 2020 2020.

248. Nicholson P, Alshafai L, Krings T. Neuroimaging Findings in Patients with COVID-19. AJNR Am J Neuroradiol. 2020 2020.

249. Nilsson A, Edner N, Albert J, Ternhag A. Fatal encephalitis associated with coronavirus OC43 in an immunocompromised child. Infectious Diseases. 2020:1-4.

250. Noorwali ASA, Turkistani AHM, Asiri SI, et al. Descriptive epidemiology and characteristics of confirmed cases of Middle East respiratory syndrome coronavirus infection in the Makkah Region of Saudi Arabia, March to June 2014. Annals of Saudi Medicine. 2015 2015;35(3):203-9.

251. Noro F, Cardoso FM, Marchiori E. COVID-19 and benign intracranial hypertension: A case report. Rev Soc Bras Med Trop. 2020 2020;53:e20200325.

252. Novi G, Rossi T, Pedemonte E, et al. Acute disseminated encephalomyelitis after SARS-CoV-2 infection. Neurology(R) neuroimmunology & neuroinflammation. 2020 2020;7(5).

253. Oguz-Akarsu E, Ozpar R, Mirzayev H, et al. Guillain-Barré Syndrome in a Patient With Minimal Symptoms of COVID-19 Infection. Muscle and Nerve. 2020 2020.

254. Okusaga O, Yolken RH, Langenberg P, et al. Association of seropositivity for influenza and coronaviruses with history of mood disorders and suicide attempts. J Affect Disord. Apr;130(1):220-5.

255. Oliveira RMC, Santos DH, Olivetti BC, Takahashi JT. Bilateral trochlear nerve palsy due to cerebral vasculitis related to COVID-19 infection. Arq Neuropsiquiatr. 2020 2020;78(6):385-6.

256. Ollarves-Carrero MF, Rodriguez-Morales AG, Bonilla-Aldana DK, Rodriguez-Morales AJ. Anosmia in a healthcare worker with COVID-19 in Madrid, Spain. Travel Med Infect Dis. 2020 2020;35:101666.

257. Ottaviani D, Boso F, Tranquillini E, et al. Early Guillain-Barré syndrome in coronavirus disease 2019 (COVID-19): a case report from an Italian COVID-hospital. Neurological Sciences. 2020:1.

258. Ottaviano G, Carecchio M, Scarpa B, Marchese-Ragona R. Olfactory and rhinological evaluations in SARS-CoV-2 patients complaining of olfactory loss. Rhinology. Apr-27.

259. Oxley TJ, Mocco J, Majidi S, et al. Large-Vessel Stroke as a Presenting Feature of Covid-19 in the Young. N Engl J Med. 2020 2020;382(20):e60.

260. Padroni M, Mastrangelo V, Asioli GM, et al. Guillain-Barré syndrome following COVID-19: new infection, old complication? Journal of Neurology. 2020:1.

261. Palomar-Ciria N, Blanco del Valle P, Hernández-Las Heras MÁ, Martínez-Gallardo R. Schizophrenia and COVID-19 delirium. Psychiatry Research. 2020 2020;290.

262. Panariello A, Bassetti R, Radice A, et al. Anti-NMDA receptor encephalitis in a psychiatric Covid-19 patient: A case report. Brain Behav Immun. 2020 2020;87:179-81.

263. Paniz-Mondolfi A, Bryce C, Grimes Z, et al. Central nervous system involvement by severe acute respiratory syndrome coronavirus-2 (SARS-CoV-2). J Med Virol. 2020 Jul;92(7):699-702.

264. Papi C, Spagni G, Alexandre A, Calabresi P, Della Marca G, Broccolini A. Unprotected stroke management in an undiagnosed case of Severe Acute Respiratory Syndrome Coronavirus 2 infection. Journal of Stroke and Cerebrovascular Diseases. 2020 2020;29(9).

265. Parsons T, Banks S, Bae C, Gelber J, Alahmadi H, Tichauer M. COVID-19-associated acute disseminated encephalomyelitis (ADEM). J Neurol. 2020 2020:1-4.

266. Paterson RW, Brown RL, Benjamin L, et al. The emerging spectrum of COVID-19 neurology: clinical, radiological and laboratory findings. Brain : a journal of neurology. 2020 Jul 8.

267. Paybast S, Gorji R, Mavandadi S. Guillain-Barré Syndrome as a Neurological Complication of Novel COVID-19 Infection: A Case Report and Review of the Literature. Neurologist. 2020 2020;25(4):101-3.

268. Peng L, Liu KY, Xue F, Miao YF, Tu PA, Zhou C. Improved Early Recognition of Coronavirus Disease-2019 (COVID-19): Single-Center Data from a Shanghai Screening Hospital. Arch Iran Med. Apr-1;23(4):272-6.

269. Pérez Álvarez Á I, Suárez Cuervo C, Fernández Menéndez S. SARS-CoV-2 infection associated with diplopia and anti-acetylcholine receptor antibodies. Neurologia. 2020 2020.

270. Pfefferkorn T, Dabitz R, von Wernitz-Keibel T, Aufenanger J, Nowak-Machen M, Janssen H. Acute polyradiculoneuritis with locked-in syndrome in a patient with Covid-19. J Neurol. 2020 2020;267(7):1883-4.

271. Pilotto A, Odolini S, Masciocchi S, et al. Steroid-Responsive Encephalitis in Coronavirus Disease 2019. Ann Neurol. 2020 2020.

272. Pinna P, Grewal P, Hall JP, et al. Neurological manifestations and COVID-19: Experiences from a tertiary care center at the Frontline. J Neurol Sci. 2020 2020;415:116969.

273. Pinto AA, Carroll LS, Nar V, Varatharaj A, Galea I. CNS inflammatory vasculopathy with antimyelin oligodendrocyte glycoprotein antibodies in COVID-19. Neurology(R) neuroimmunology & neuroinflammation. 2020 2020;7(5).

274. Poillon G, Obadia M, Perrin M, Savatovsky J, Lecler A. Cerebral Venous Thrombosis associated with COVID-19 infection: causality or coincidence? Journal of neuroradiology = Journal de neuroradiologie. 2020 2020.

275. Pokorn M, Jevšnik M, Petrovec M, et al. Respiratory and Enteric Virus Detection in Children. J Child Neurol. Jan;32(1):84-93.

276. Politi LS, Salsano E, Grimaldi M. Magnetic Resonance Imaging Alteration of the Brain in a Patient With Coronavirus Disease 2019 (COVID-19) and Anosmia. JAMA Neurol. 2020 2020.

277. Pons-Escoda A, Naval-Baudín P, Majós C, et al. Neurologic Involvement in COVID-19: Cause or Coincidence? A Neuroimaging Perspective. AJNR Am J Neuroradiol. 2020 2020.

278. Poyiadji N, Shahin G, Noujaim D, Stone M, Patel S, Griffith B. COVID-19-associated Acute Hemorrhagic Necrotizing Encephalopathy: CT and MRI Features. Radiology. 2020 Mar 31:201187.

279. Princiotta Cariddi L, Tabaee Damavandi P, Carimati F, et al. Reversible Encephalopathy Syndrome (PRES) in a COVID-19 patient. J Neurol. 2020 2020:1-4.

280. Qin C, Zhou L, Hu Z, et al. Dysregulation of immune response in patients with COVID-19 in Wuhan, China. Clin Infect Dis. Mar-12.

281. Rábano-Suárez P, Bermejo-Guerrero L, Méndez-Guerrero A, et al. Generalized myoclonus in COVID-19. Neurology. 2020 2020.

282. Radmanesh A, Derman A, Ishida K. COVID-19-associated delayed posthypoxic necrotizing leukoencephalopathy. Journal of the Neurological Sciences. 2020 2020;415.

283. Radmanesh A, Derman A, Lui YW, et al. COVID-19 -associated Diffuse Leukoencephalopathy and Microhemorrhages. Radiology. 2020 2020:202040.

284. Radmanesh A, Raz E, Zan E, Derman A, Kaminetzky M. Brain Imaging Use and Findings in COVID-19: A Single Academic Center Experience in the Epicenter of Disease in the United States. AJNR Am J Neuroradiol. 2020 2020;41(7):1179-83.

285. Rana S, Lima AA, Chandra R, et al. Novel Coronavirus (COVID-19)-Associated Guillain-Barré Syndrome: Case Report. J Clin Neuromuscul Dis. 2020 2020;21(4):240-2.

286. Reddy ST, Reddy ST, Garg T, et al. Cerebrovascular Disease in Patients with COVID-19: A Review of the Literature and Case Series. Case Reports in Neurology. 2020 2020:199-209.

287. Regev T, Antebi M, Eytan D, et al. Pediatric Inflammatory Multisystem Syndrome With Central Nervous System Involvement and Hypocomplementemia Following SARS-COV-2 Infection. Pediatr Infect Dis J. 2020 2020;39(8):e206-e7.

288. Reichard RR, Kashani KB, Boire NA, Constantopoulos E, Guo Y, Lucchinetti CF. Neuropathology of COVID-19: a spectrum of vascular and acute disseminated encephalomyelitis (ADEM)-like pathology. Acta Neuropathol. 2020 Jul;140(1):1-6.

289. Reyes-Bueno JA, García-Trujillo L, Urbaneja P, et al. Miller-Fisher syndrome after SARS-CoV-2 infection. Eur J Neurol. 2020 Jun 5.

290. Rigamonti A, Mantero V, Piamarta F, Spena G, Salmaggi A. Cerebral venous thrombosis associated with coronavirus infection: an underestimated entity? Neurol Sci. 2020 2020:1-2.

291. Riski H, Hovi T. Coronavirus infections of man associated with diseases other than the common cold. J Med Virol. 1980 1980;6(3):259-65.

292. Riva N, Russo T, Falzone YM, et al. Post-infectious Guillain-Barré syndrome related to SARS-CoV-2 infection: a case report. J Neurol. 2020 2020:1-3.

293. Rogg J, Baker A, Tung G. Posterior reversible encephalopathy syndrome (PRES): Another imaging manifestation of COVID-19. Interdisciplinary Neurosurgery: Advanced Techniques and Case Management. 2020 2020;22.

294. Romero-Sánchez CM, Díaz-Maroto I, Fernández-Díaz E, et al. Neurologic manifestations in hospitalized patients with COVID-19: The ALBACOVID registry. Neurology. 2020 Jun 1.

295. Roy-Gash F, Marine M, Jean-Michel D, Herve V, Raphael B, Nicolas E. COVID-19-associated acute cerebral venous thrombosis: clinical, CT, MRI and EEG features. Crit Care. 2020 2020;24(1):419.

296. Rudilosso S, Esteller D, Urra X, Chamorro Á. Thalamic perforating artery stroke on computed tomography perfusion in a patient with coronavirus disease 2019. Journal of Stroke and Cerebrovascular Diseases. 2020 2020;29(8).

297. Saad M, Omrani AS, Baig K, et al. Clinical aspects and outcomes of 70 patients with Middle East respiratory syndrome coronavirus infection: a single-center experience in Saudi Arabia. International Journal of Infectious Diseases. 2014;29:301-6.

298. Sachs JR, Gibbs KW, Swor DE, et al. COVID-19-Associated Leukoencephalopathy. Radiology. 2020 2020:201753.

299. Saggese CE, Del Bianco C, Di Ruzza MR, Magarelli M, Gandini R, Plocco M. COVID-19 and Stroke: Casual or Causal Role? Cerebrovasc Dis. 2020 2020:1-4.

300. Salmi A, Ziola B, Hovi T, Reunanen M. Antibodies to coronaviruses OC43 and 229E in multiple sclerosis patients. Neurology. Mar;32(3):292-5.

301. Sancho-Saldaña A, Lambea-Gil Á, Liesa JLC, et al. Guillain-Barré syndrome associated with leptomeningeal enhancement following SARS-CoV-2 infection. Clinical medicine (London, England). 2020 Jun 9.

302. Sangalli D, Polonia V, Colombo D, et al. A single-centre experience of intravenous thrombolysis for stroke in COVID-19 patients. Neurol Sci. 2020 2020:1-5.

303. Scheidl E, Canseco DD, Hadji‐Naumov A, Bereznai B. Guillain‐Barre syndrome during SARS‐CoV‐2 pandemic: a case report and review of recent literature. Journal of the Peripheral Nervous System. 2020.

304. Schupper AJ, Yaeger KA, Morgenstern PF. Neurological manifestations of pediatric multi-system inflammatory syndrome potentially associated with COVID-19. Child's Nervous System. 2020 2020.

305. Scullen T, Keen J, Mathkour M, Dumont AS, Kahn L. Coronavirus 2019 (COVID-19)-Associated Encephalopathies and Cerebrovascular Disease: The New Orleans Experience. World Neurosurg. 2020 2020.

306. Sedaghat Z, Karimi N. Guillain Barre syndrome associated with COVID-19 infection: A case report. J Clin Neurosci. Apr-15.

307. Selvaraj V, Sacchetti D, Finn A, Dapaah-Afriyie K. Acute Vision Loss in a Patient with COVID-19. R I Med J (2013). 2020 2020;103(6):37-8.

308. Severance EG, Dickerson FB, Viscidi RP, et al. Coronavirus immunoreactivity in individuals with a recent onset of psychotic symptoms. Schizophr Bull. Jan;37(1):101-7.

309. Sharifi-Razavi A, Karimi N, Rouhani N. COVID-19 and intracerebral haemorrhage: causative or coincidental? New Microbes New Infect. May;35:100669.

310. Sharifi-Razavi A, Karimi N, Zarvani A, Cheraghmakani H, Baghbanian SM. Ischemic stroke associated with novel coronavirus 2019: a report of three cases. Int J Neurosci. 2020 2020:1-5.

311. Sharma K, Tengsupakul S, Sanchez O, Phaltas R, Maertens P. Guillain-Barré syndrome with unilateral peripheral facial and bulbar palsy in a child: A case report. SAGE Open Med Case Rep. 2019 2019;7:2050313x19838750.

312. Sheng B, Cheng SKW, Lau KK, Li HL, Chan ELY. The effects of disease severity, use of corticosteroids and social factors on neuropsychiatric complaints in severe acute respiratory syndrome (SARS) patients at acute and convalescent phases. European psychiatry. 2005;20(3):236-42.

313. Shoskes A, Migdady I, Fernandez A, Ruggieri P, Rae-Grant A. Cerebral Microhemorrhage and Purpuric Rash in COVID-19: The Case for a Secondary Microangiopathy. Journal of Stroke and Cerebrovascular Diseases. 2020 2020;29(10).

314. Sierpiński R, Pinkas J, Jankowski M, et al. Sex differences in the frequency of gastrointestinal symptoms and olfactory or taste disorders in 1942 nonhospitalized patients with coronavirus disease 2019 (COVID-19). Pol Arch Intern Med. 2020 2020;130(6):501-5.

315. Sierra-Hidalgo F, Muñoz-Rivas N, Torres Rubio P, et al. Large artery ischemic stroke in severe COVID-19. Journal of Neurology. 2020 2020.

316. Singh J, Ali A. Headache as the Presenting Symptom in 2 Patients with COVID-19 and a History of Migraine: 2 Case Reports. Headache. 2020 Jun 10.

317. Singh S, Govindarajan R. COVID-19 and generalized Myasthenia Gravis exacerbation: A case report. Clin Neurol Neurosurg. 2020 2020;196:106045.

318. Sohal S, Mansur M. COVID-19 Presenting with Seizures. IDCases. 2020 2020;20.

319. Soldatelli MD, Amaral LFD, Veiga VC, Rojas SSO, Omar S, Marussi VHR. Neurovascular and perfusion imaging findings in coronavirus disease 2019: Case report and literature review. Neuroradiol J. 2020 2020:1971400920941652.

320. Solomon IH, Normandin E, Bhattacharyya S, et al. Neuropathological Features of Covid-19. New England Journal of Medicine. 2020.

321. Somani S, Pati S, Gaston T, Chitlangia A, Agnihotri S. De Novo Status Epilepticus in patients with COVID-19. Ann Clin Transl Neurol. 2020 2020;7(7):1240-4.

322. Sorensen O, Collins A, Flintoff W, Ebers G, Dales S. Probing for the human coronavirus OC43 in multiple sclerosis. Neurology. Dec;36(12):1604-6.

323. Sotoca J, Rodríguez-Álvarez Y. COVID-19-associated acute necrotizing myelitis. Neurology(R) neuroimmunology & neuroinflammation. 2020 2020;7(5).

324. Speth MM, Singer-Cornelius T, Oberle M, Gengler I, Brockmeier SJ, Sedaghat AR. Mood, anxiety and olfactory dysfunction in COVID-19: evidence of central nervous system involvement? Laryngoscope. 2020 2020.

325. Speth MM, Singer-Cornelius T, Oberle M, Gengler I, Brockmeier SJ, Sedaghat AR. Olfactory Dysfunction and Sinonasal Symptomatology in COVID-19: Prevalence, Severity, Timing, and Associated Characteristics. Otolaryngol Head Neck Surg. 2020 2020;163(1):114-20.

326. Spoldi C, Castellani L, Pipolo C, et al. Isolated olfactory cleft involvement in SARS-CoV-2 infection: prevalence and clinical correlates. Eur Arch Otorhinolaryngol. 2020 2020:1-4.

327. Stainsby B, Howitt S, Porr J. Neuromusculoskeletal disorders following SARS: a case series. J Can Chiropr Assoc. Mar;55(1):32-9.

328. Stewart JN, Mounir S, Talbot PJ. Human coronavirus gene expression in the brains of multiple sclerosis patients. Virology. Nov;191(1):502-5.

329. Su XW, Palka SV, Rao RR, Chen FS, Brackney CR, Cambi F. SARS-CoV-2-associated Guillain-Barré syndrome with dysautonomia. Muscle Nerve. 2020 2020;62(2):E48-e9.

330. Sun D, Li H, Lu XX, et al. Clinical features of severe pediatric patients with coronavirus disease 2019 in Wuhan: a single center's observational study. World J Pediatr. Mar-19.

331. Sweid A, Hammoud B, Bekelis K, et al. Cerebral ischemic and hemorrhagic complications of coronavirus disease 2019. International Journal of Stroke.10.

332. Tang YM, Xing YG, Li JG, Ding YQ, Lei CL, Tang XP. Clinical analysis of neuropsychiatric involement in severe acute respiratory syndrome. Chinese Journal of Neurology. 2004 2004;37(3):228-30.

333. Tapé C, Byrd KM, Aung S, Lonks JR, Flanigan TP, Rybak NR. COVID-19 in a Patient Presenting with Syncope and a Normal Chest X-ray. R I Med J (2013). 2020 2020;103(3):50-1.

334. Tatu L, Nono S, Grácio S, Koçer S. Guillain–Barré syndrome in the COVID-19 era: another occasional cluster? Journal of Neurology. 2020 2020.

335. Torabi A, Mohammadbagheri E, Akbari Dilmaghani N, et al. Proinflammatory Cytokines in the Olfactory Mucosa Result in COVID-19 Induced Anosmia. ACS Chem Neurosci. 2020 2020;11(13):1909-13.

336. Toscano G, Palmerini F, Ravaglia S, et al. Guillain-Barré Syndrome Associated with SARS-CoV-2. N Engl J Med. 2020 2020;382(26):2574-6.

337. Tsai LK, Hsieh ST, Chao CC, et al. Neuromuscular disorders in severe acute respiratory syndrome. Arch Neurol. Nov;61(11):1669-73.

338. Tsivgoulis G, Fragkou PC, Delides A, et al. Quantitative evaluation of olfactory dysfunction in hospitalized patients with Coronavirus [2] (COVID-19). J Neurol. 2020 2020;267(8):2193-5.

339. TunÇ A, ÜNlÜBaŞ Y, Alemdar M, AkyÜ ZE. Coexistence of COVID-19 and acute ischemic stroke report of four cases. Journal of Clinical Neuroscience. 2020 2020.

340. Turbin RE, Wawrzusin PJ, Sakla NM, et al. Orbital cellulitis, sinusitis and intracranial abnormalities in two adolescents with COVID-19. Orbit. 2020 2020;39(4):305-10.

341. Turgay C, Emine T, Ozlem K, Muhammet SP, Haydar AT. A rare cause of acute flaccid paralysis: Human coronaviruses. J Pediatr Neurosci. Jul-Sep;10(3):280-1.

342. Umapathi T, Kor AC, Venketasubramanian N, et al. Large artery ischaemic stroke in severe acute respiratory syndrome (SARS). J Neurol. Oct;251(10):1227-31.

343. Utukuri PS, Bautista A, Lignelli A, Moonis G. Possible Acute Disseminated Encephalomyelitis Related to Severe Acute Respiratory Syndrome Coronavirus 2 Infection. AJNR Am J Neuroradiol. 2020 2020.

344. Vacchiano V, Riguzzi P, Volpi L, et al. Early neurological manifestations of hospitalized COVID-19 patients. Neurol Sci. 2020 2020:1-3.

345. Vaira LA, Deiana G, Fois AG, et al. Objective evaluation of anosmia and ageusia in COVID-19 patients: Single-center experience on 72 cases. Head Neck. 2020 2020;42(6):1252-8.

346. Vaira LA, Salzano G, Deiana G, De Riu G. Anosmia and Ageusia: Common Findings in COVID-19 Patients. Laryngoscope. 2020 2020;130(7):1787.

347. Valderrama EV, Humbert K, Lord A, Frontera J, Yaghi S. Severe Acute Respiratory Syndrome Coronavirus 2 Infection and Ischemic Stroke. Stroke. 2020:STROKEAHA. 120.030153.

348. Varatharaj A, Thomas N, Ellul MA, et al. Neurological and neuropsychiatric complications of COVID-19 in 153 patients: a UK-wide surveillance study. The Lancet Psychiatry. 2020 2020.

349. Vargas-Gandica J, Winter D, Schnippe R, et al. Ageusia and anosmia, a common sign of COVID-19? A case series from four countries. J Neurovirol. 2020 2020:1-5.

350. Velayos Galán A, del Saz Saucedo P, Peinado Postigo F, Botia Paniagua E. Guillain-Barré syndrome associated with SARS-CoV-2 infection. Neurologia. 2020 2020.

351. Vellieux G, Rouvel-Tallec A, Jaquet P, Grinea A, Sonneville R, d'Ortho MP. COVID-19 associated encephalopathy: Is there a specific EEG pattern? Clinical Neurophysiology. 2020 2020;131(8):1928-30.

352. Vespignani H, Colas D, Lavin BS, et al. Report on Electroencephalographic Findings in Critically Ill Patients with COVID-19. Ann Neurol. 2020 2020.

353. Viguier A, Delamarre L, Duplantier J, Olivot JM, Bonneville F. Acute ischemic stroke complicating common carotid artery thrombosis during a severe COVID-19 infection. Journal of neuroradiology. 2020.

354. Virhammar J, Kumlien E, Fällmar D, et al. Acute necrotizing encephalopathy with SARS-CoV-2 RNA confirmed in cerebrospinal fluid. Neurology. 2020 2020.

355. Vollono C, Rollo E, Romozzi M, et al. Focal status epilepticus as unique clinical feature of COVID-19: A case report. Seizure. 2020.

356. von Weyhern CH, Kaufmann I, Neff F, Kremer M. Early evidence of pronounced brain involvement in fatal COVID-19 outcomes. Lancet. 2020 Jun 20;395(10241):e109.

357. Vu D, Ruggiero M, Choi WS, et al. Three unsuspected CT diagnoses of COVID-19. Emergency radiology. 2020:1-4.

358. Wang L, Cai J, Luo H, et al. A case of COVID-19 with tuberculous meningitis. Chinese Journal of Neurology. 2020 2020;53(5):361-4.

359. Webb S, Wallace VC, Martin-Lopez D, Yogarajah M. Guillain-Barré syndrome following COVID-19: a newly emerging post-infectious complication. BMJ Case Rep. 2020 2020;13(6).

360. Wei H, Yin H, Huang M, Guo Z. The 2019 novel cornoavirus pneumonia with onset of oculomotor nerve palsy: a case study. Journal of Neurology. 2020:1-4.

361. Wei L, Sun S, Zhang J, et al. Endocrine cells of the adenohypophysis in severe acute respiratory syndrome (SARS). Biochem Cell Biol. Aug;88(4):723-30.

362. Williams OH, Mohideen S, Sen A, et al. Multiple internal border zone infarcts in a patient with COVID-19 and CADASIL. J Neurol Sci. 2020 2020;416:116980.

363. Wong PF, Craik S, Newman P, et al. Lessons of the month 1: A case of rhombencephalitis as a rare complication of acute COVID-19 infection. Clinical Medicine. 2020;20(3):293-4.

364. Xiong W, Mu J, Guo J, et al. New onset neurologic events in people with COVID-19 infection in three regions in China. Neurology. 2020 2020.

365. Xu J, Zhong S, Liu J, et al. Detection of severe acute respiratory syndrome coronavirus in the brain: potential role of the chemokine mig in pathogenesis. Clin Infect Dis. Oct-15;41(8):1089-96.

366. Yaghi S, Ishida K, Torres J, et al. SARS-CoV-2 and Stroke in a New York Healthcare System. Stroke. 2020 2020;51(7):2002-11.

367. Yeh EA, Collins A, Cohen ME, Duffner PK, Faden H. Detection of coronavirus in the central nervous system of a child with acute disseminated encephalomyelitis. Pediatrics. Jan;113(1):e73-6.

368. Yin R, Feng W, Wang T, et al. Concomitant neurological symptoms observed in a patient diagnosed with coronavirus disease 2019. J Med Virol. Apr-15.

369. Zachariadis A, Tulbu A, Strambo D, Dumoulin A, Di Virgilio G. Transverse myelitis related to COVID-19 infection. Journal of Neurology. 2020 2020.

370. Zahid MJ, Baig A, Galvez-Jimenez N, Martinez N. Hemorrhagic Stroke in Setting of Severe COVID-19 Infection Requiring Extracorporeal Membrane Oxygenation (ECMO). Journal of Stroke and Cerebrovascular Diseases. 2020 2020;29(9).

371. Zanin L, Saraceno G, Panciani PP, et al. SARS-CoV-2 can induce brain and spine demyelinating lesions. Acta Neurochirurgica. 2020:1-4.

372. Zayet S, Ben Abdallah Y, Royer PY, Toko-Tchiundzie L, Gendrin V, Klopfenstein T. Encephalopathy in patients with COVID-19: 'Causality or coincidence?'. J Med Virol. 2020 2020.

373. Zayet S, Klopfenstein T, Kovẚcs R, Stancescu S, Hagenkötter B. Acute Cerebral Stroke with Multiple Infarctions and COVID-19, France, 2020. Emerg Infect Dis. 2020 2020;26(9).

374. Zhai P, Ding Y, Li Y. The impact of COVID-19 on ischemic stroke. Diagn Pathol. 2020 2020;15(1):78.

375. Zhang H, Charmchi Z, Seidman RJ, Anziska Y, Velayudhan V, Perk J. COVID-19 associated myositis with severe proximal and bulbar weakness. Muscle and Nerve. 2020 2020.

376. Zhao H, Shen D, Zhou H, Liu J, Chen S. Guillain-Barré syndrome associated with SARS-CoV-2 infection: causality or coincidence? Lancet Neurol. May;19(5):383-4.

377. Zhou B, She J, Wang Y, Ma X. A case of coronavirus disease 2019 with concomitant acute cerebral infarction and deep vein thrombosis. Frontiers in Neurology. 2020;11.

378. Zoghi A, Ramezani M, Roozbeh M, Darazam IA, Sahraian MA. A case of possible atypical demyelinating event of the central nervous system following COVID-19. Mult Scler Relat Disord. 2020 2020;44:102324.
