# Supplementary material for: Neurological manifestations of coronavirus infections – a systematic review
Source: Ann Clin Transl Neurol. 2020 Aug 27;7(10):2057–71. doi: 10.1002/acn3.51166 (PMC7461163; doi:10.1002/acn3.51166)
Supplement: Supplementary file 3 — Data S2. Search string for Medline, Embase, and Web of Science. [file ACN3-7-2057-s003.docx]

**Neurological manifestations and neuroimaging of coronavirus infections – A systematic review**

Search date: 26^th^ of July,.2020

Pubmed via Medline:

(coronavirus OR HCoV OR SARS-CoV-1 OR SARS-CoV-2 OR SARS-CoV1 OR SARS-CoV2 OR SARS) AND (neuroimaging OR magnetic resonance imaging OR MRI OR Neurology OR neurological complications OR neuroimmunology OR neurotropism OR neurologic OR central nervous system OR CNS OR head-CT OR brain-CT OR Positron emission tomography OR PET OR Nervous System Diseases [MeSH])

References: 2966

Embase:

("coronavirus" OR "HCoV" OR "SARS-CoV-1" OR "SARS-CoV-2" OR "SARS-CoV1" OR "SARS-CoV2" OR "SARS") AND ("neuroimaging" OR "magnetic resonance imaging" OR "MRI" OR "Neurolog*" OR "neurological complications" OR "neuroimmunology" OR "neurotropism" OR "central nervous system" OR "CNS" OR "head-CT" OR "brain-CT" OR "Positron emission tomography" OR "PET" OR "neurologic disease")

References: 5416

Web of Science:

TS=((coronavirus OR HCoV OR SARS-CoV-1 OR SARS-CoV-2 OR SARS-CoV1 OR SARS-CoV2 OR SARS) AND (neuroimaging OR magnetic resonance imaging OR MRI OR Neurology OR neurological complications OR neuroimmunology OR neurotropism OR neurologic OR central nervous system OR CNS OR head-CT OR brain-CT OR Positron emission tomography OR PET OR Nervous System Diseases))

References: 3813
